# Supplementary material for: Evolution of Barrett’s esophagus through space and time at single-crypt and whole-biopsy levels
Source: Nat Commun. 2018 Feb 23;9:794. doi: 10.1038/s41467-017-02621-x (PMC5824808; doi:10.1038/s41467-017-02621-x)
Supplement: Supplementary file 1 — Supplementary Information [file 41467_2017_2621_MOESM1_ESM.pdf]

SUPPLEMENTARY FIGURES

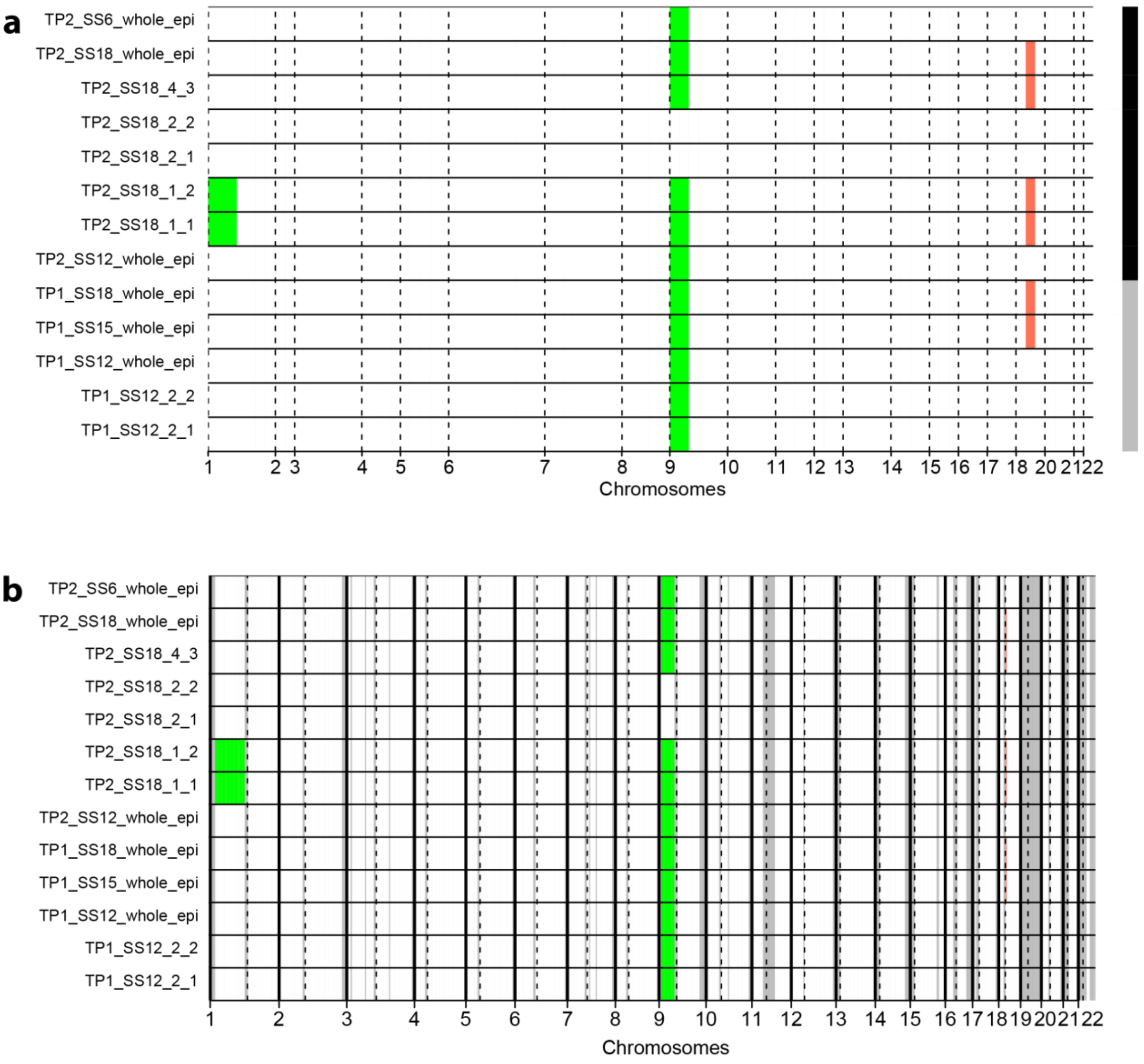

Supplementary Figure 1: Patient 256-NP allele-specific relative copy number profile. a) Scale-free segmented map. The copy number state of each segment is represented by equal sized bars in each sample. Yellow bars on the right side indicate whether a segment underwent genome doubling. Grey bars indicate samples from the first time point, black bars those from the second time point. b) Cytoband map. The dominant state of each minor cytoband in the genome is displayed by a single equal-sized bar in each sample. States are indicated by: blue = loss; red = gain; green = copy neutral LOH. Paler / darker colours indicate the different alleles impacted by each copy number change. Segmentation is shared across all samples and copy number states are relative to the sample's reported ploidy.

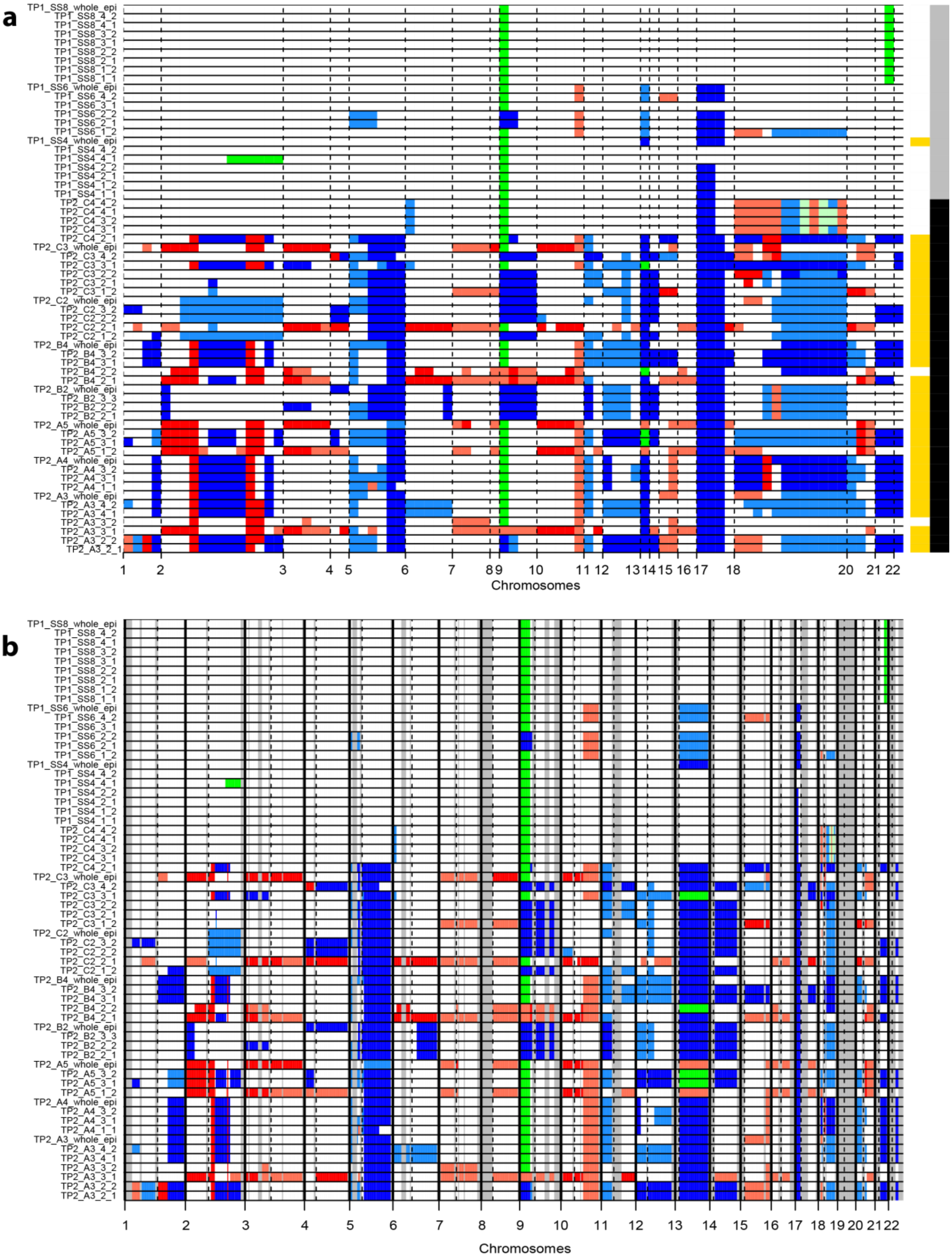

Supplementary Figure 2: Patient 391-P allele-specific relative copy number profile. a) Scale-free segmented map. The copy number state of each segment is represented by equal sized bars in each sample. Yellow bars on the right side indicate whether a segment underwent genome doubling. Grey bars indicate samples from the first time point, black bars those from the second time point. b) Cytoband map. The dominant state of each minor cytoband in the genome is displayed by a single equal-sized bar in each sample. States are indicated by: blue = loss; red = gain; green = copy neutral LOH. Paler / darker colours indicate the different alleles impacted by each copy number change. Segmentation is shared across all samples and copy number states are relative to the sample's reported ploidy.

a

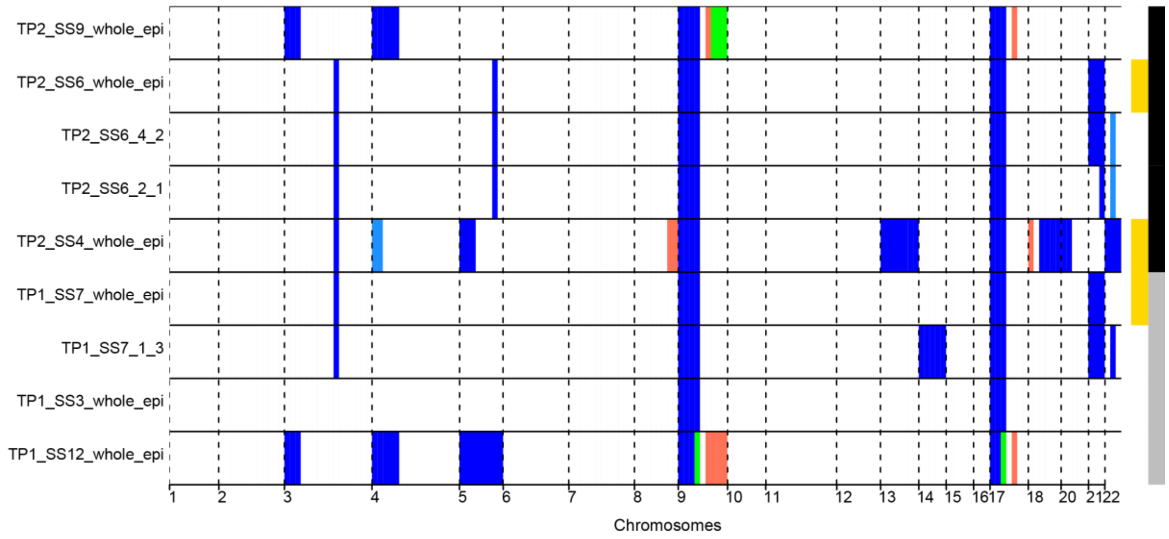

b

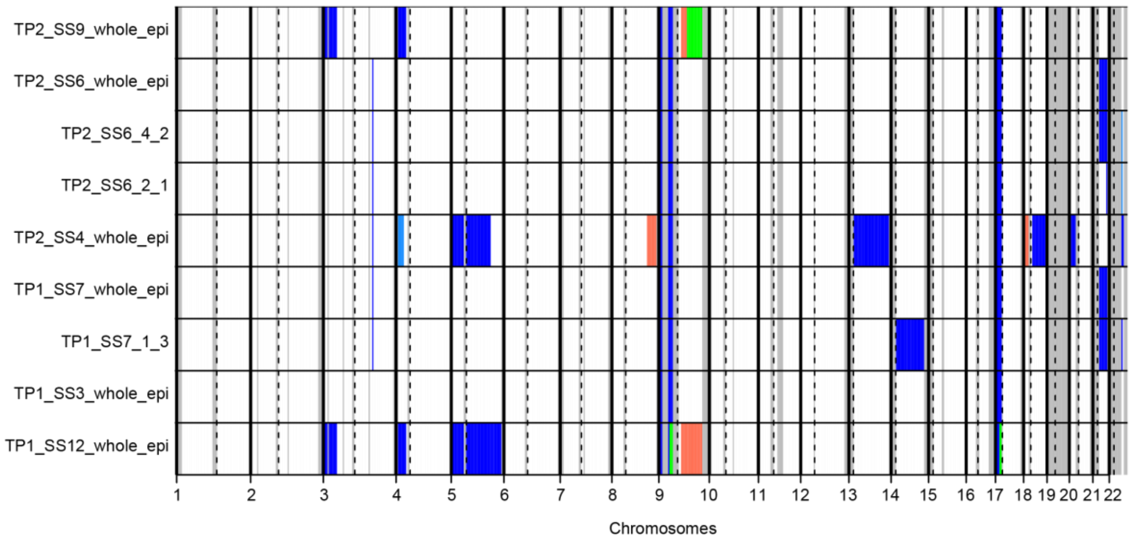

Supplementary Figure 3: Patient 437-NP allele-specific relative copy number profile. a) Scale-free segmented map. The copy number state of each segment is represented by equal sized bars in each sample. Yellow bars on the right side indicate whether a segment underwent genome doubling. Grey bars indicate samples from the first time point, black bars those from the second time point. b) Cytoband map. The dominant state of each minor cytoband in the genome is displayed by a single equal-sized bar in each sample. States are indicated by: blue = loss; red = gain; green = copy neutral LOH. Paler / darker colours indicate the different alleles impacted by each copy number change. Segmentation is shared across all samples and copy number states are relative to the sample's reported ploidy.

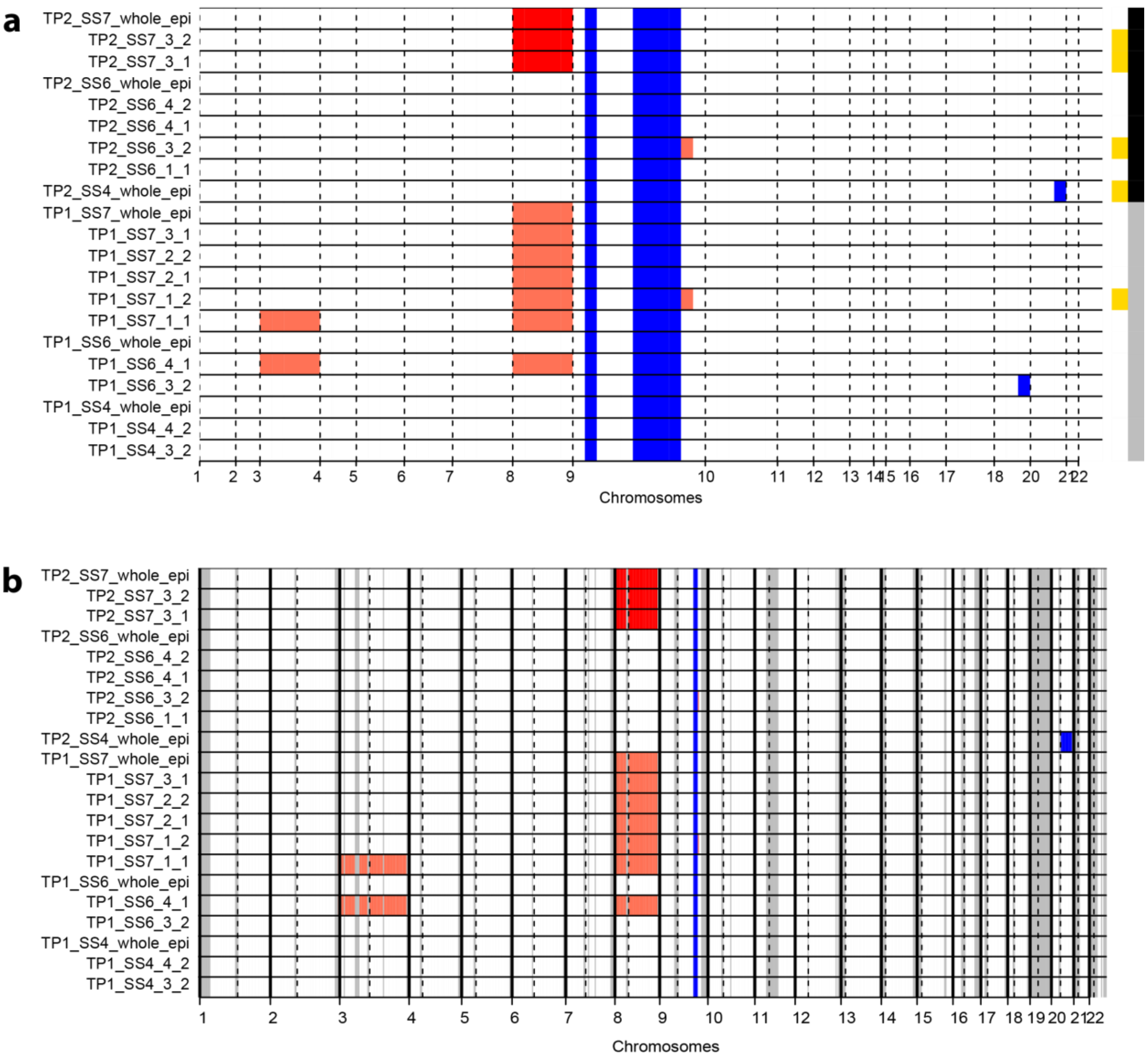

Supplementary Figure 4: Patient 451-NP allele-specific relative copy number profile. a) Scale-free segmented map. The copy number state of each segment is represented by equal sized bars in each sample. Yellow bars on the right side indicate whether a segment underwent genome doubling. Grey bars indicate samples from the first time point, black bars those from the second time point. b) Cytoband map. The dominant state of each minor cytoband in the genome is displayed by a single equal-sized bar in each sample. States are indicated by: blue = loss; red = gain; green = copy neutral LOH. Paler / darker colours indicate the different alleles impacted by each copy number change. Segmentation is shared across all samples and copy number states are relative to the sample's reported ploidy.

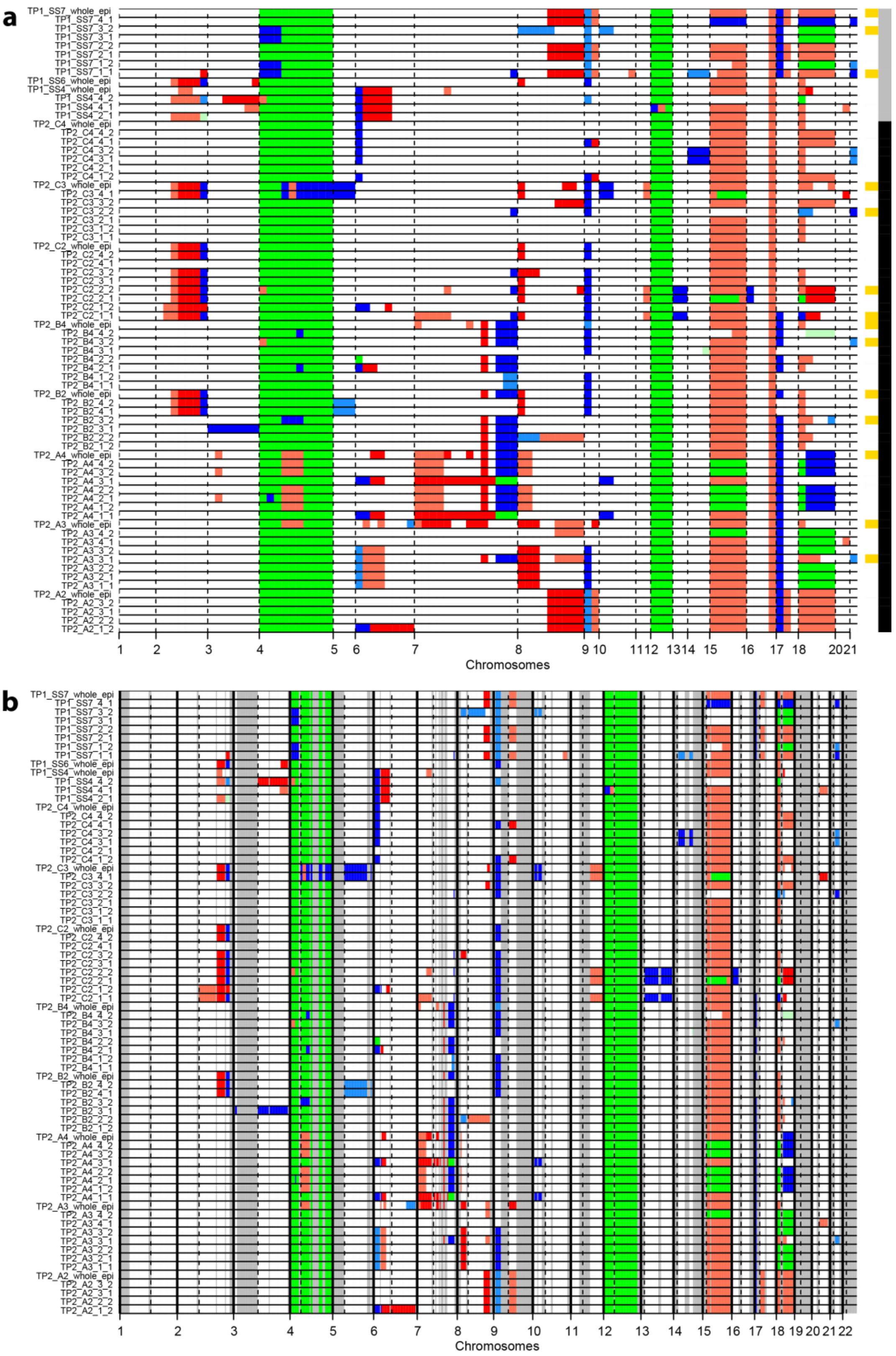

Supplementary Figure 5: Patient 740-P allele-specific relative copy number profile. a) Scale-free segmented map. The copy number state of each segment is represented by equal sized bars in each sample. Yellow bars on the right side indicate whether a segment underwent genome doubling. Grey bars indicate samples from the first time point, black bars those from the second time point. b) Cytoband map. The dominant state of each minor cytoband in the genome is displayed by a single equal-sized bar in each sample. States are indicated by: blue = loss; red = gain; green = copy neutral LOH. Paler / darker colours indicate the different alleles impacted by each copy number change. Segmentation is shared across all samples and copy number states are relative to the sample's reported ploidy.

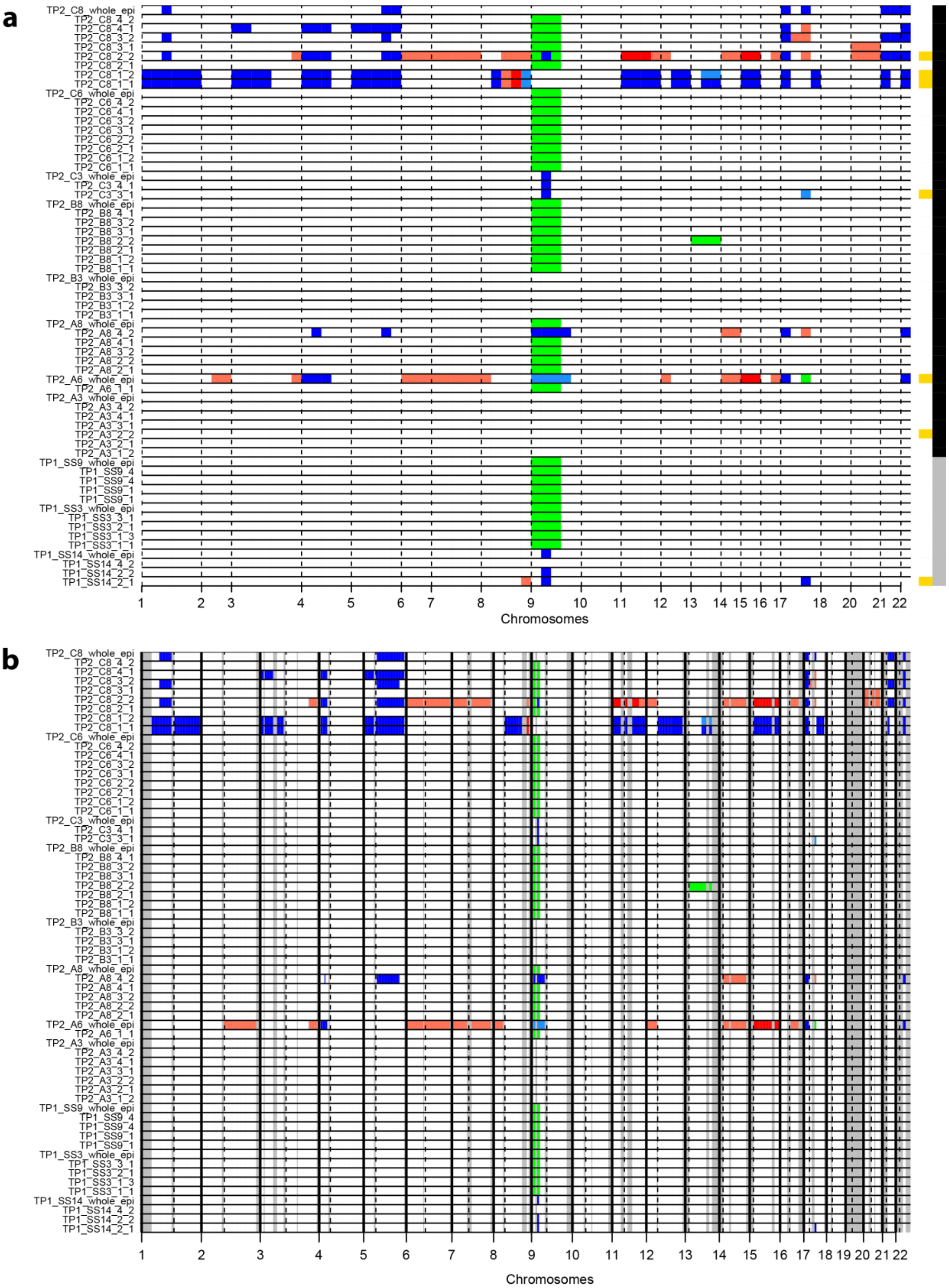

Supplementary Figure 6: Patient 848-P allele-specific relative copy number profile. a) Scale-free segmented map. The copy number state of each segment is represented by equal sized bars in each sample. Yellow bars on the right side indicate whether a segment underwent genome doubling. Grey bars indicate samples from the first time point, black bars those from the second time point. b) Cytoband map. The dominant state of each minor cytoband in the genome is displayed by a single equal-sized bar in each sample. States are indicated by: blue = loss; red = gain; green = copy neutral LOH. Paler / darker colours indicate the different alleles impacted by each copy number change. Segmentation is shared across all samples and copy number states are relative to the sample's reported ploidy.

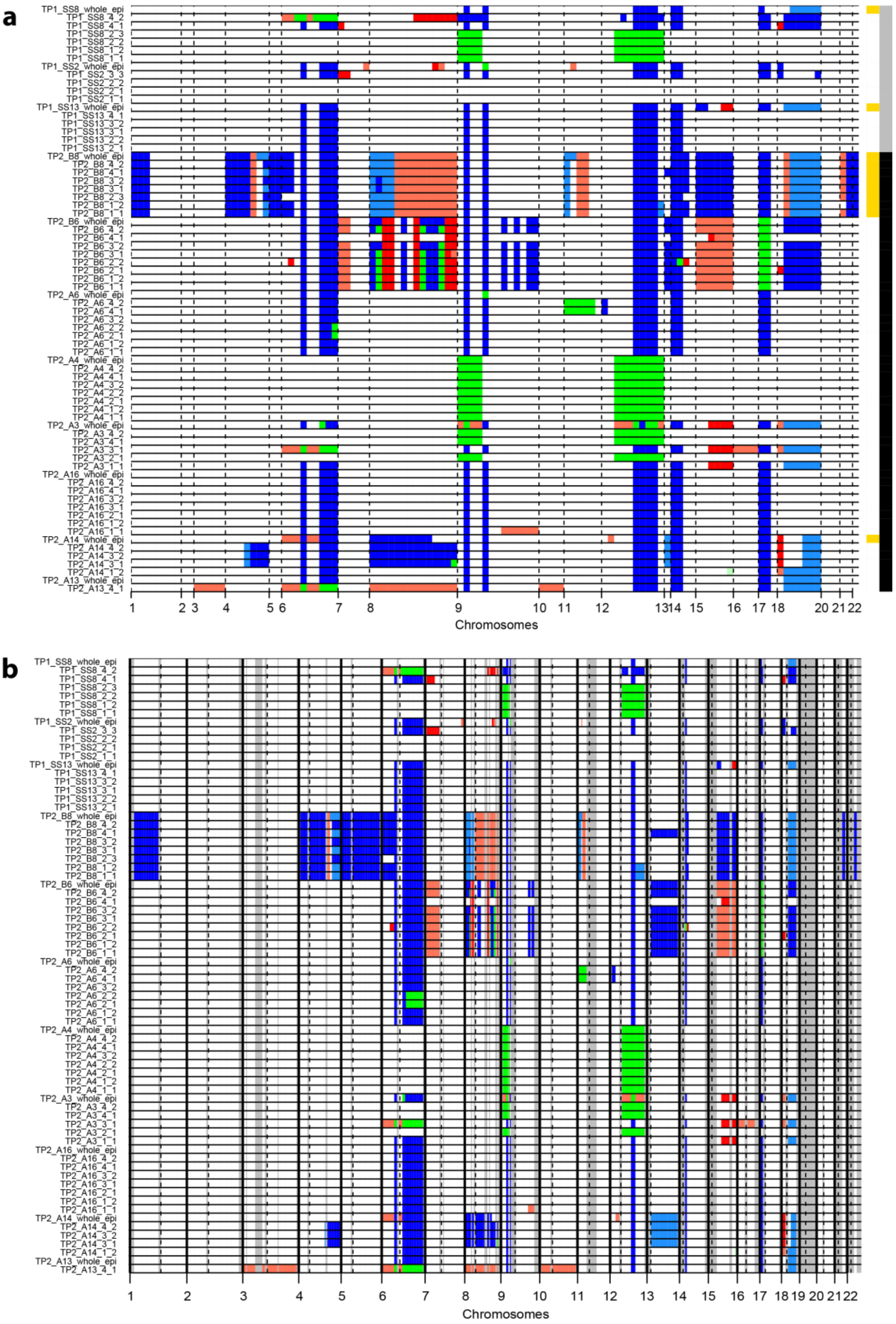

Supplementary Figure 7: Patient 852-P allele-specific relative copy number profile. a Scale-free segmented map. The copy number state of each segment is represented by equal sized bars in each sample. Yellow bars on the right side indicate whether a segment underwent genome doubling. Grey bars indicate samples from the first time point, black bars those from the second time point. b) Cytoband map. The dominant state of each minor cytoband in the genome is displayed by a single equal-sized bar in each sample. States are indicated by: blue = loss; red = gain; green = copy neutral LOH. Paler / darker colours indicate the different alleles impacted by each copy number change. Segmentation is shared across all samples and copy number states are relative to the sample's reported ploidy.

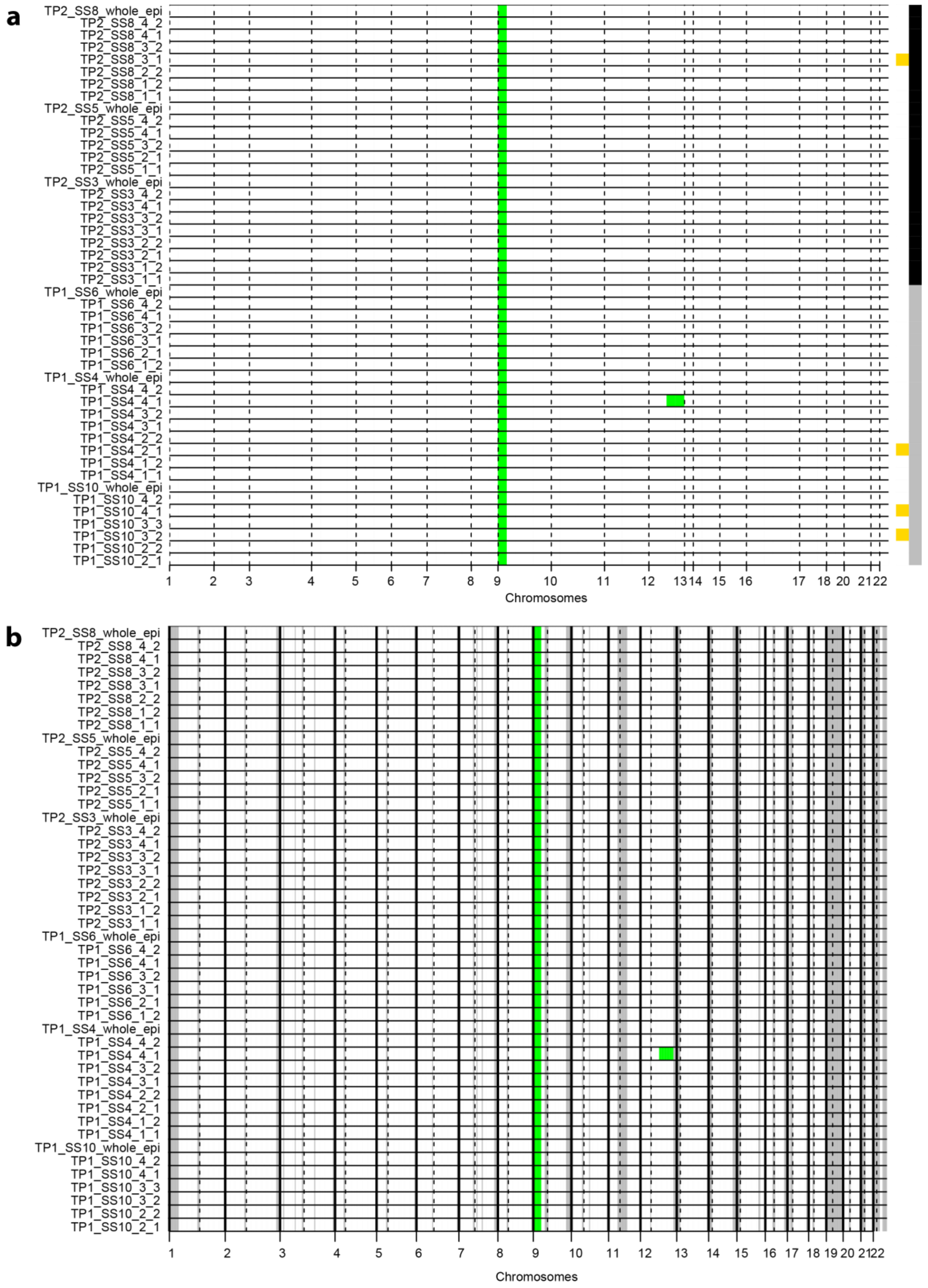

Supplementary Figure 8: Patient 911-NP allele-specific relative copy number profile. a) Scale-free segmented map. The copy number state of each segment is represented by equal sized bars in each sample. Yellow bars on the right side indicate whether a segment underwent genome doubling. Grey bars indicate samples from the first time point, black bars those from the second time point. b) Cytoband map. The dominant state of each minor cytoband in the genome is displayed by a single equal-sized bar in each sample. States are indicated by: blue = loss; red = gain; green = copy neutral LOH. Paler / darker colours indicate the different alleles impacted by each copy number change. Segmentation is shared across all samples and copy number states are relative to the sample's reported ploidy.

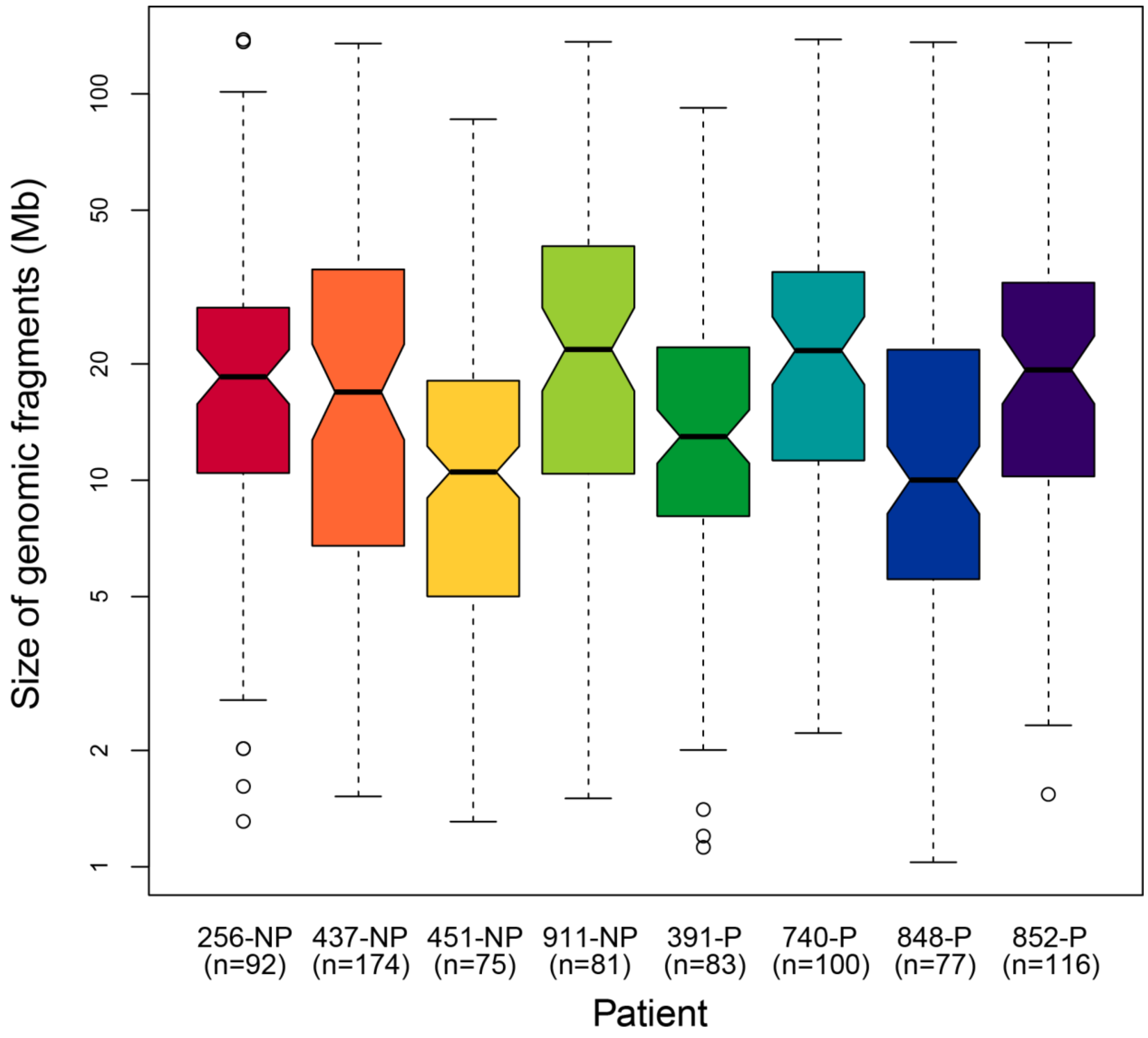

Supplementary Figure 9: Genomic segment sizes per patient. Number of segments and their sizes in megabases (Mb) in each patient after joint segmentation and quality control. Y-axis is in log10 scale. Colored boxes indicate the interquartile range (2nd and 3rd quartiles), thick horizontal black lines indicate medians. Whiskers extend to 1.5 times the interquartile range. Circles indicate outliers from the interquartile range.

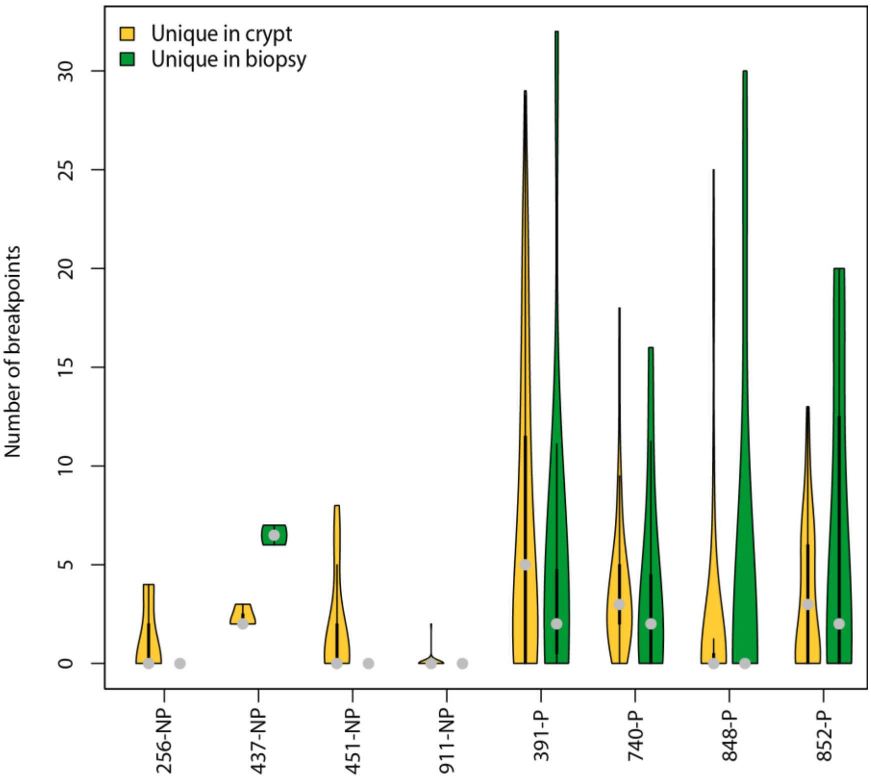

Supplementary Figure 10: Breakpoints unique to either a crypt or a whole biopsy sample. The breakpoints in all crypts are compared to the breakpoints in the whole epithelium of the biopsy they originate from. Boxplots indicate the distributions of breakpoints that are unique either to the crypt (yellow) or the whole biopsy sample (green). Boxplots inside the violin plots: boxes indicate the middle quartiles, whiskers indicate the confidence intervals and the grey dot indicates the median.

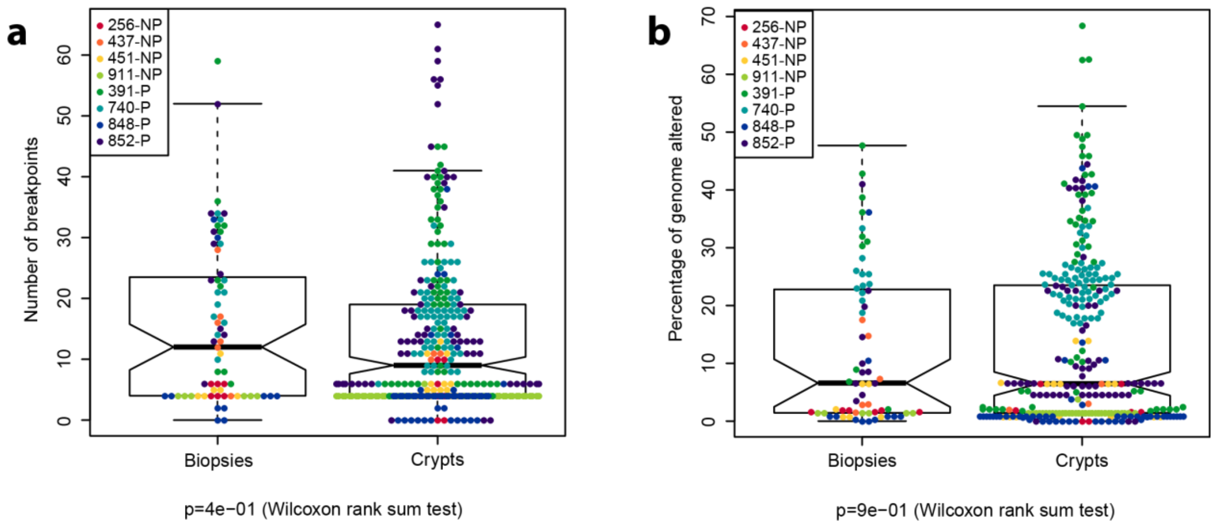

Supplementary Figure 11: Number of breakpoints and abnormal genome percentage in whole biopsy and individual crypt samples. a) Number of breakpoints. b) Abnormal genome percentage. Boxes indicate the middle quartiles, whiskers extend to 1.5 times the interquartile range and the horizontal bar indicates the median. Each sample is represented by a dot, whose colour indicates the patient of origin. Notches indicate confidence interval around the median, defined by the median ± 1.57 times the interquartile range divided by the square root of the number of observations.

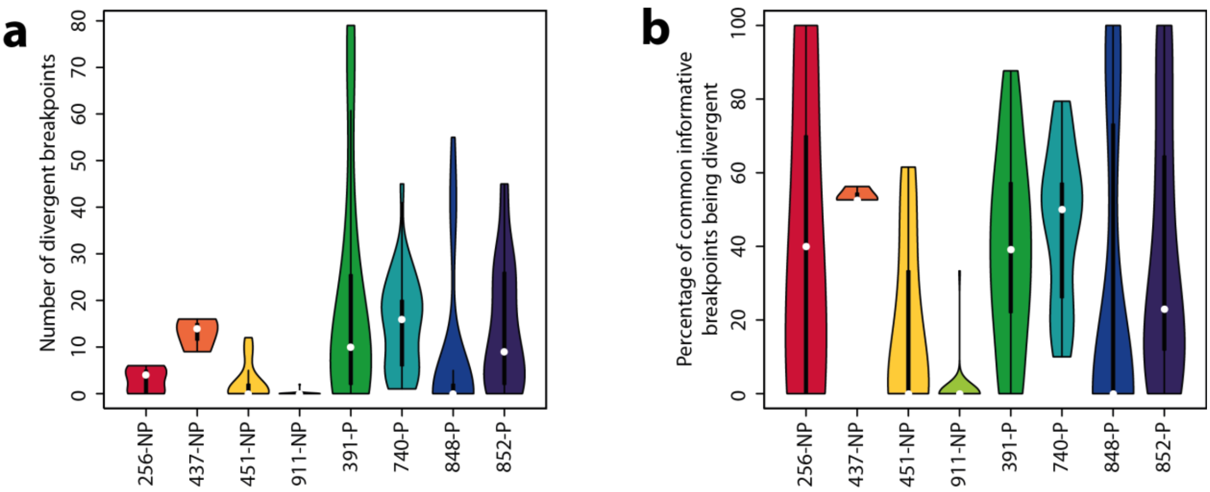

Supplementary Figure 12: Divergent breakpoints between crypt samples and the biopsy they originate from. a) Number of divergent breakpoints. b) Percentage of divergent breakpoints. Each crypt sample was compared to the biopsy sample it originates from to identify the divergent breakpoints. Thick black bars delimitate medium quartiles, thin black lines delimitate 95% confidence intervals. White squares indicate the median.

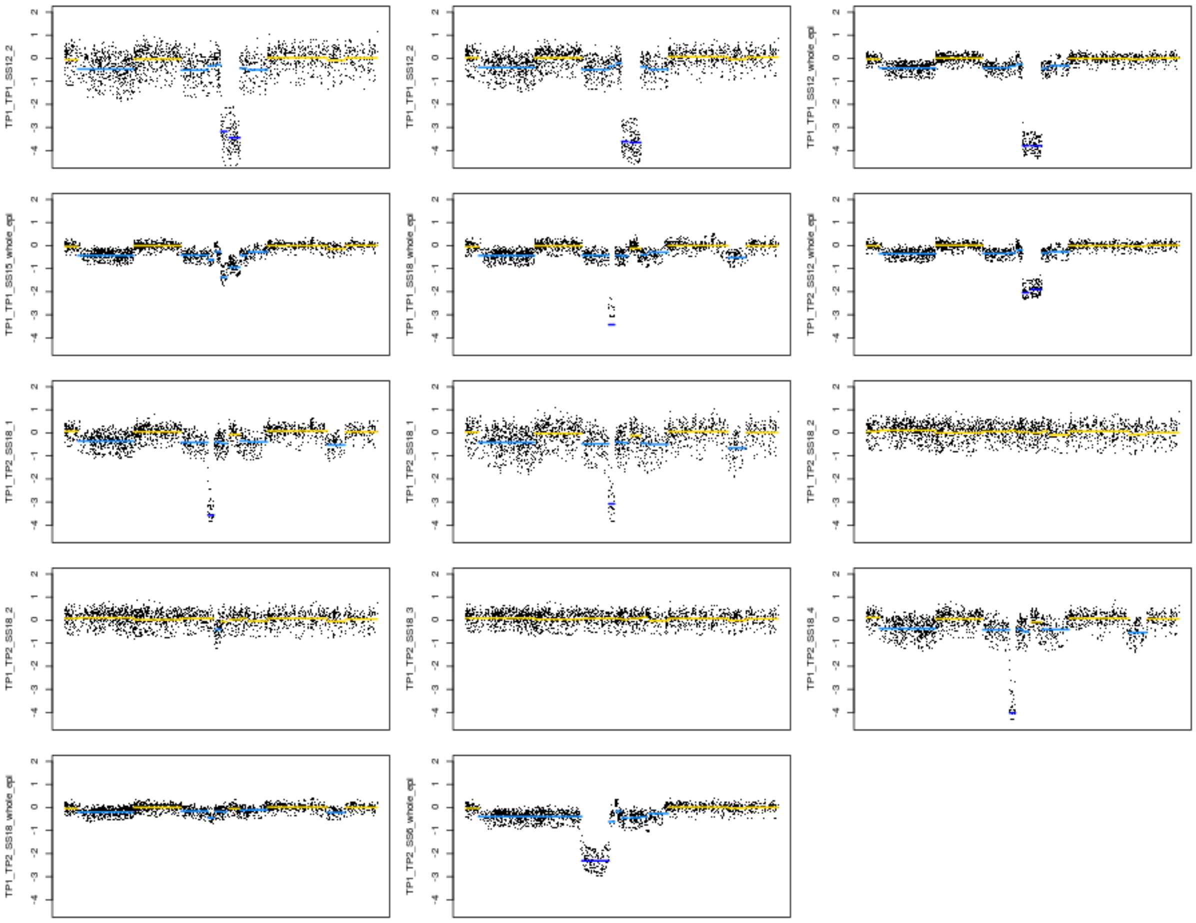

Supplementary Figure 13: Precision segmentation of logR values at the FHIT locus in patient 256-NP. Each plot represents the logR value of the locus in a sample. Each dot in a plot is the logR value for a probe on the array and is located at the related chromosomal position on the X axis. Yellow segments indicate a normal copy number, light blue ones a single copy loss, dark blue ones a double loss and red ones a gain.

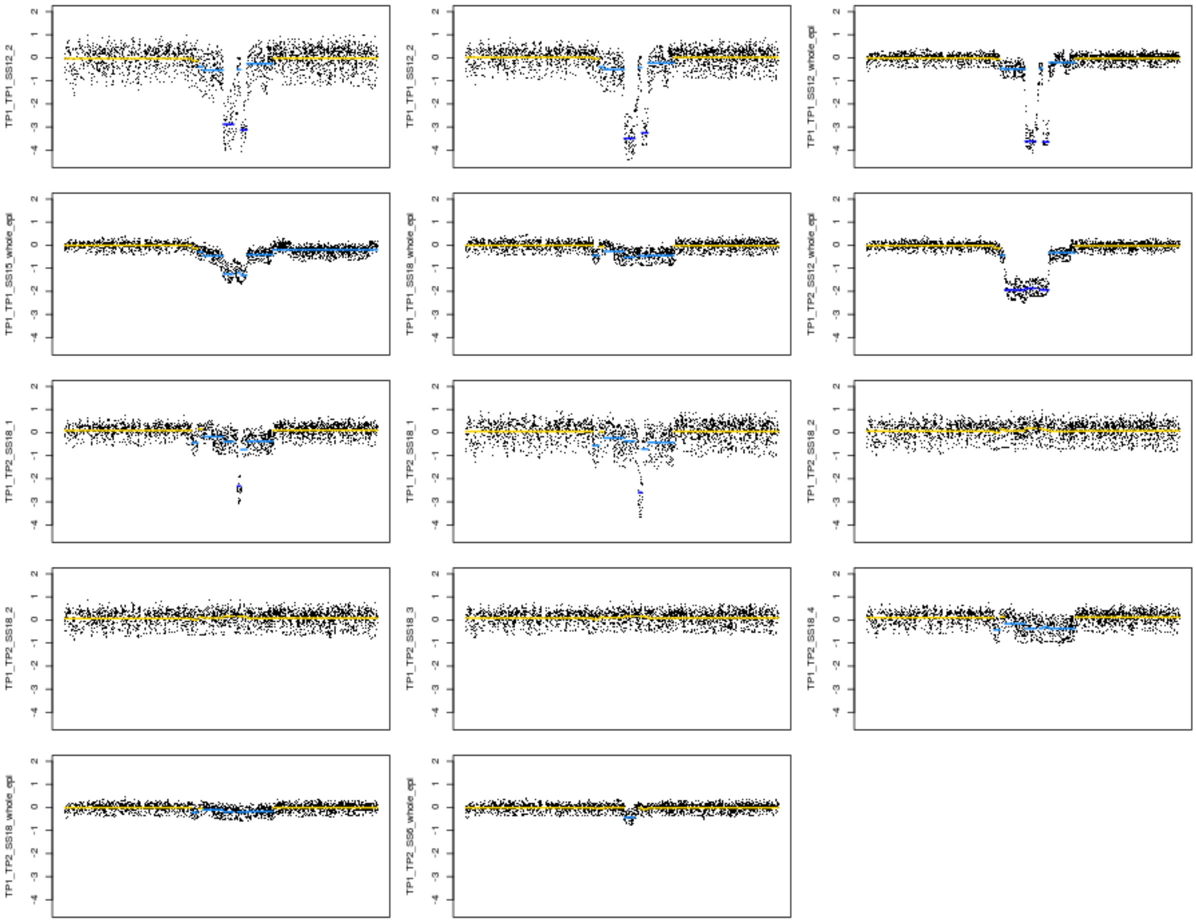

Supplementary Figure 14: Precision segmentation of logR values at the WWOX locus in patient 256-NP. Each plot represents the logR value of the locus in a sample. Each dot in a plot is the logR value for a probe on the array and is located at the related chromosomal position on the X axis. Yellow segments indicate a normal copy number, light blue ones a single copy loss, dark blue ones a double loss and red ones a gain.

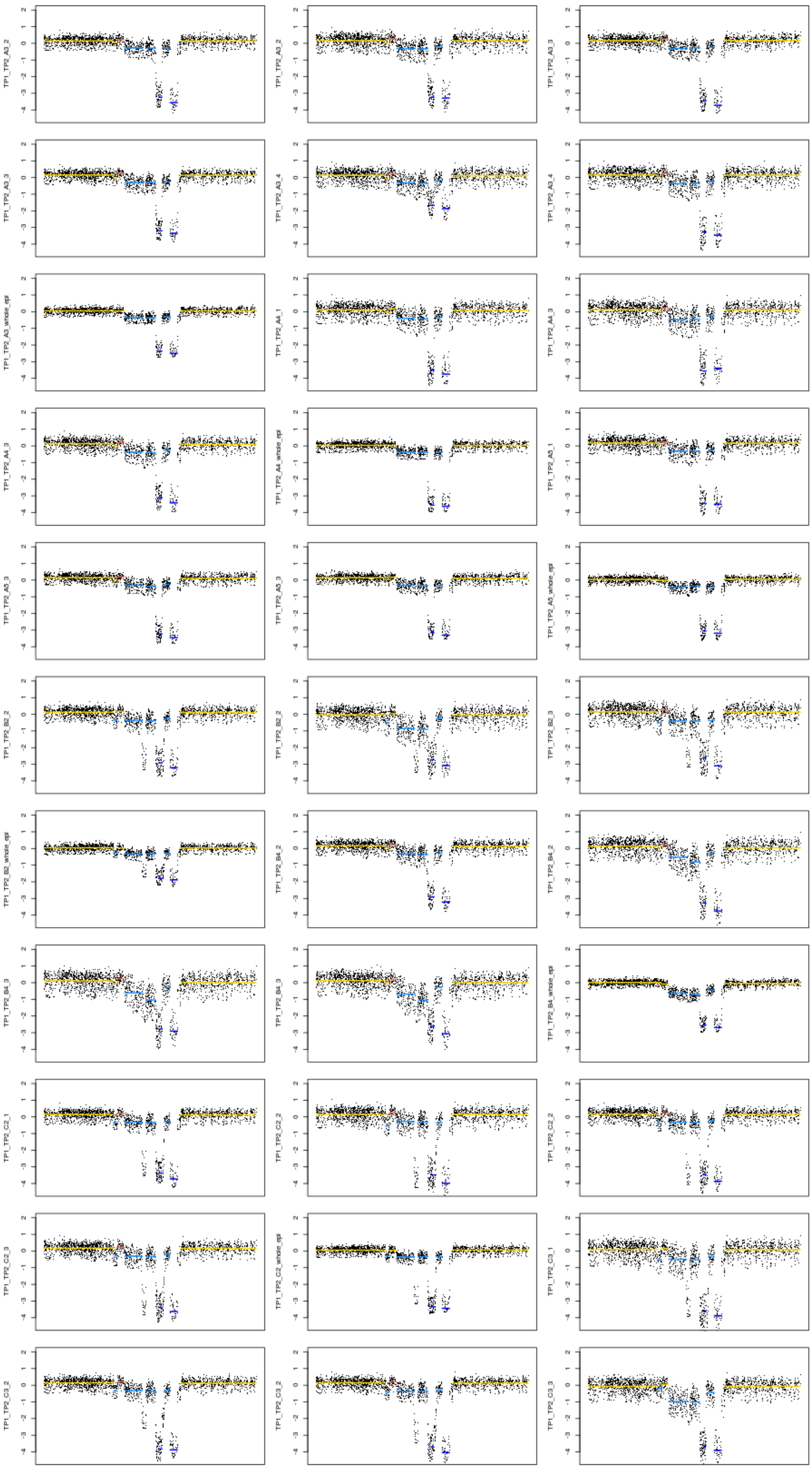

Supplementary Figure 15: Precision segmentation of logR values at the FHIT locus in patient 391-P.

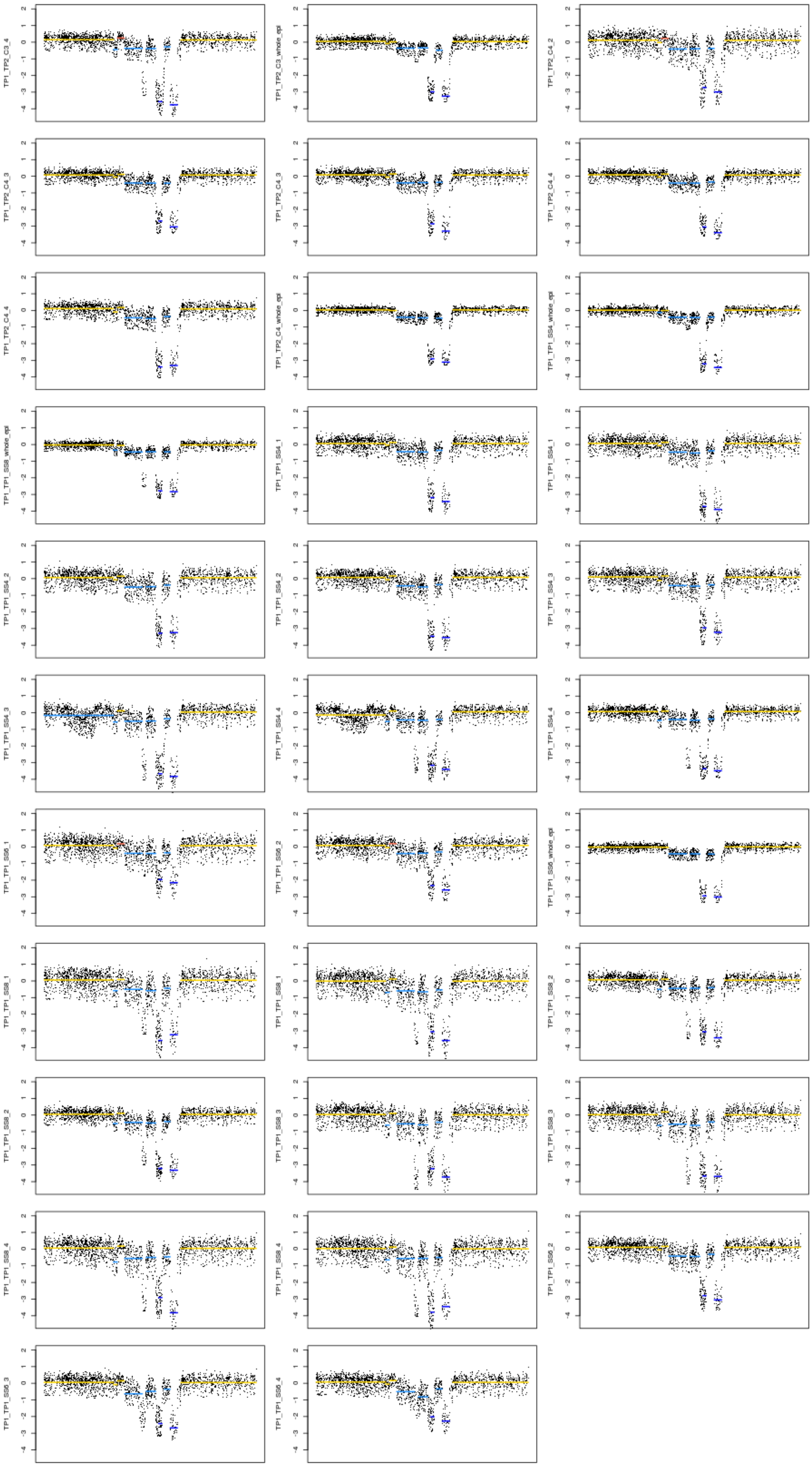

Supplementary Figure 15 (continued).

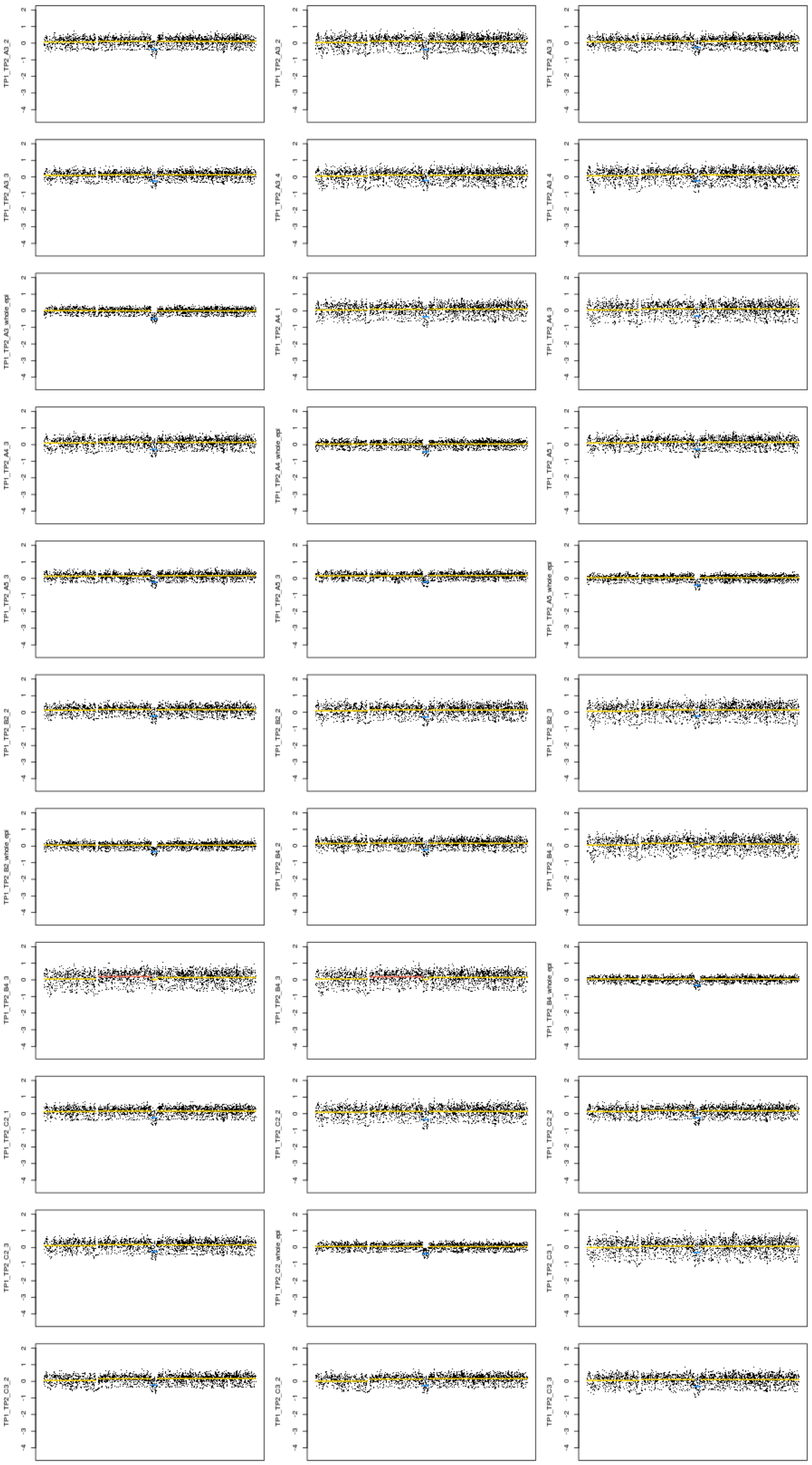

Supplementary Figure 16: Precision segmentation of logR values at the WWOX locus in patient 391-P.

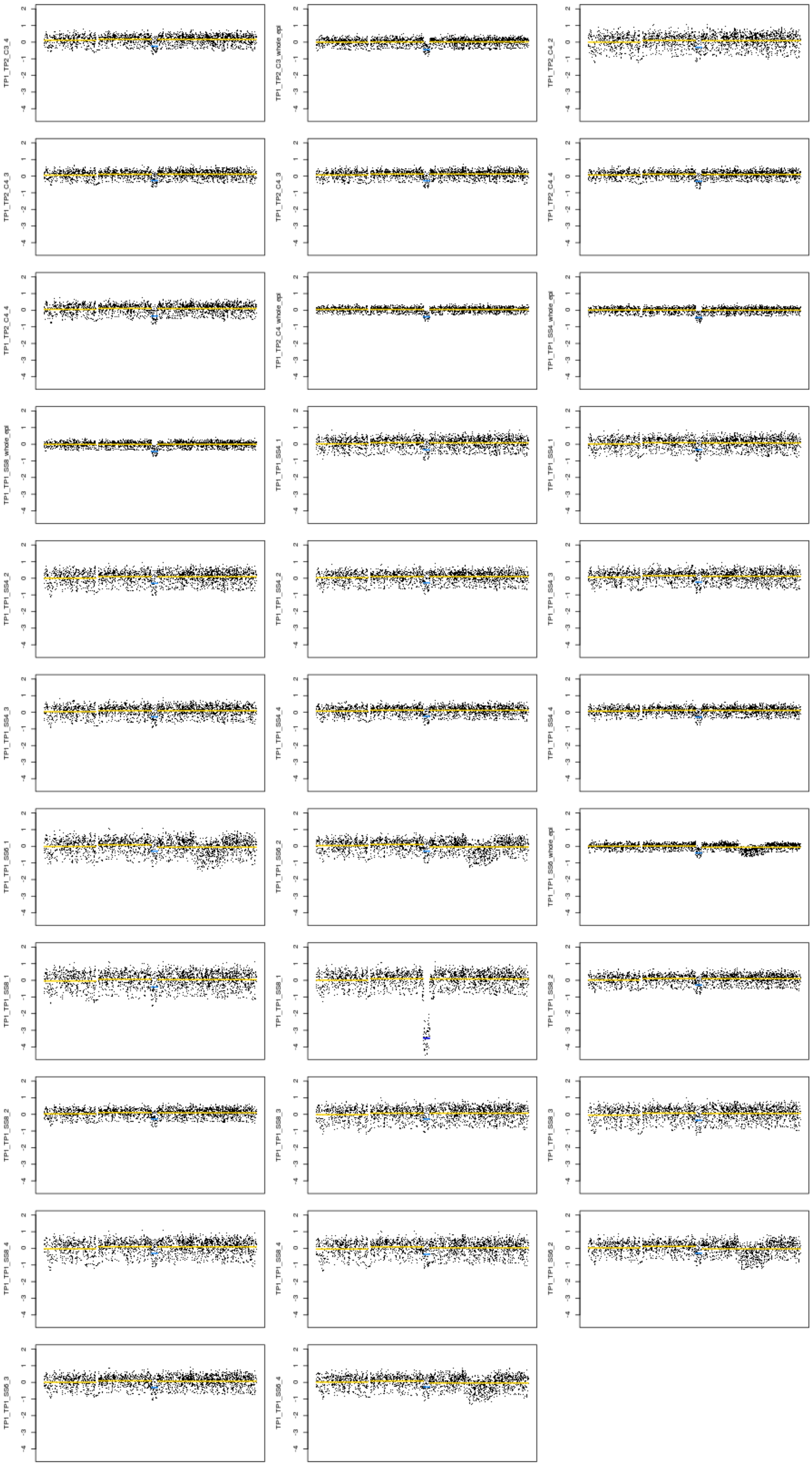

Supplementary Figure 16 (continued).

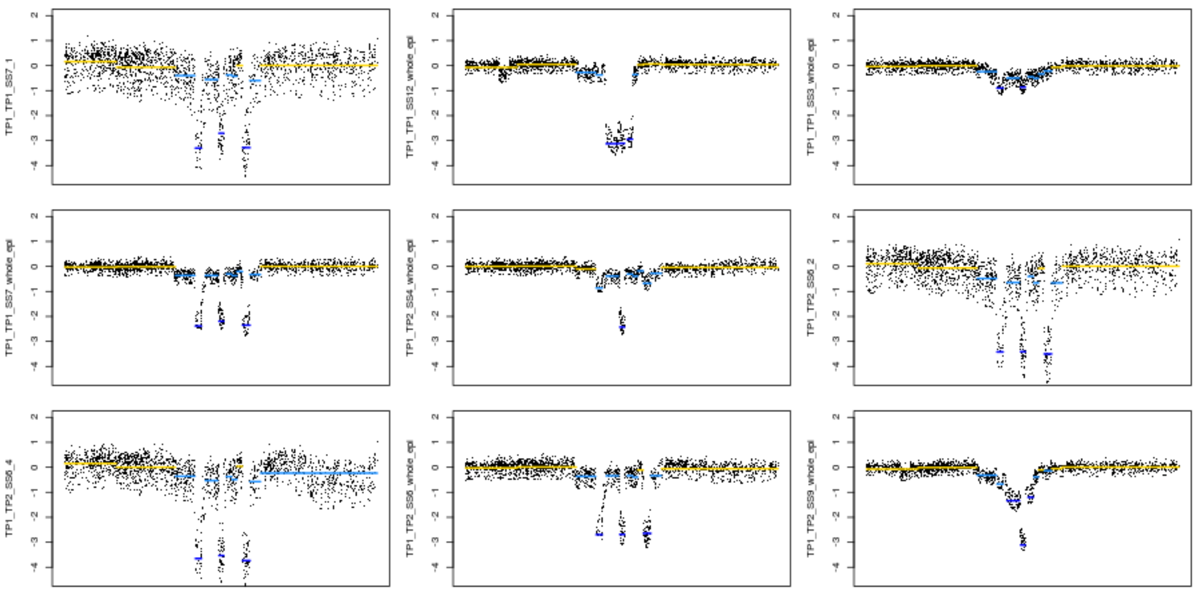

Supplementary Figure 17: Precision segmentation of logR values at the FHIT locus in patient 437-NP.

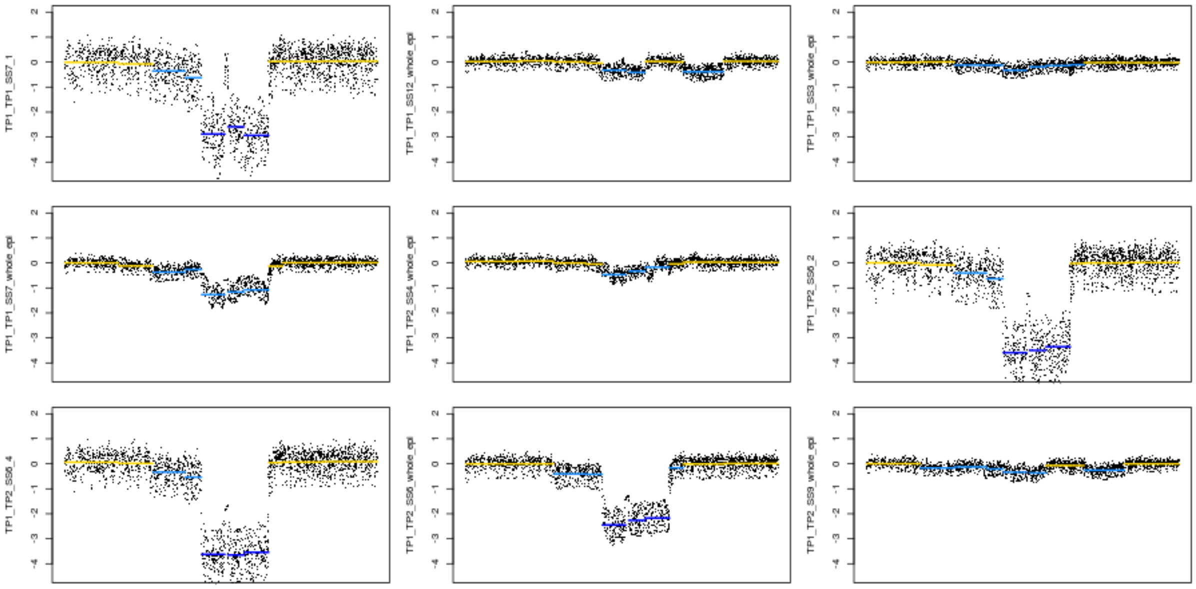

Supplementary Figure 18: Precision segmentation of logR values at the WWOX locus in patient 437-NP.

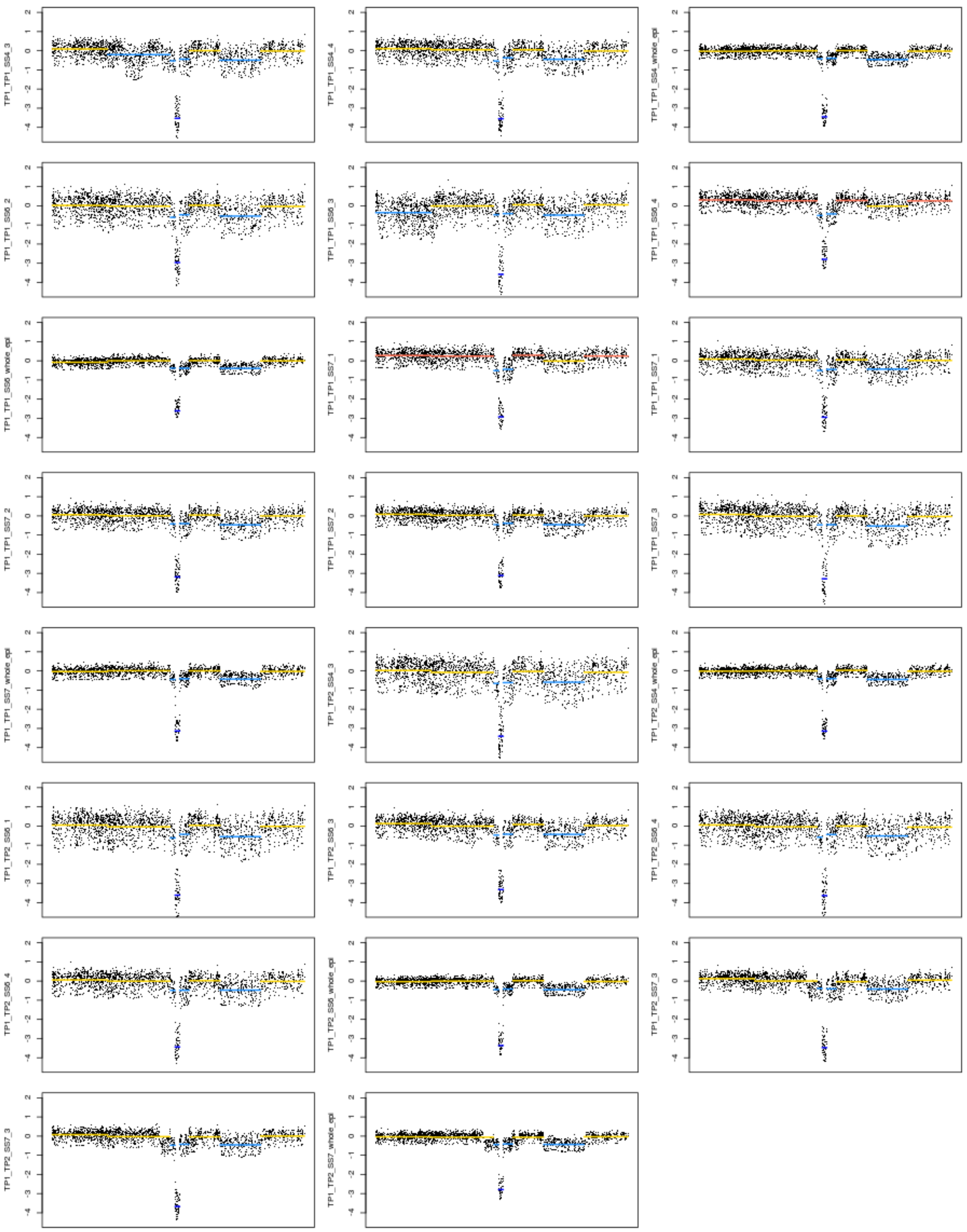

Supplementary Figure 19: Precision segmentation of logR values at the FHIT locus in patient 451-NP.

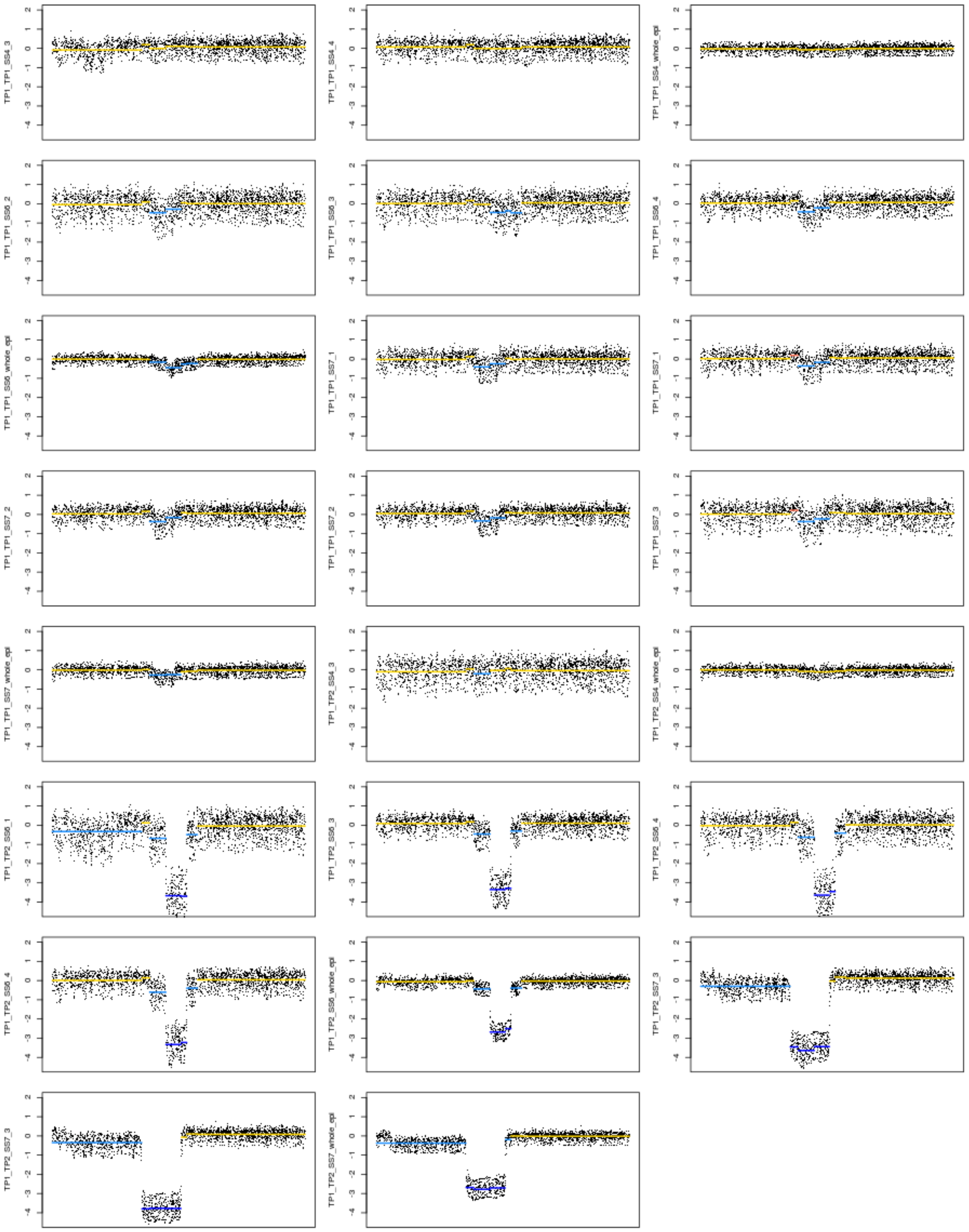

Supplementary Figure 20: Precision segmentation of logR values at the WWOX locus in patient 451-NP.

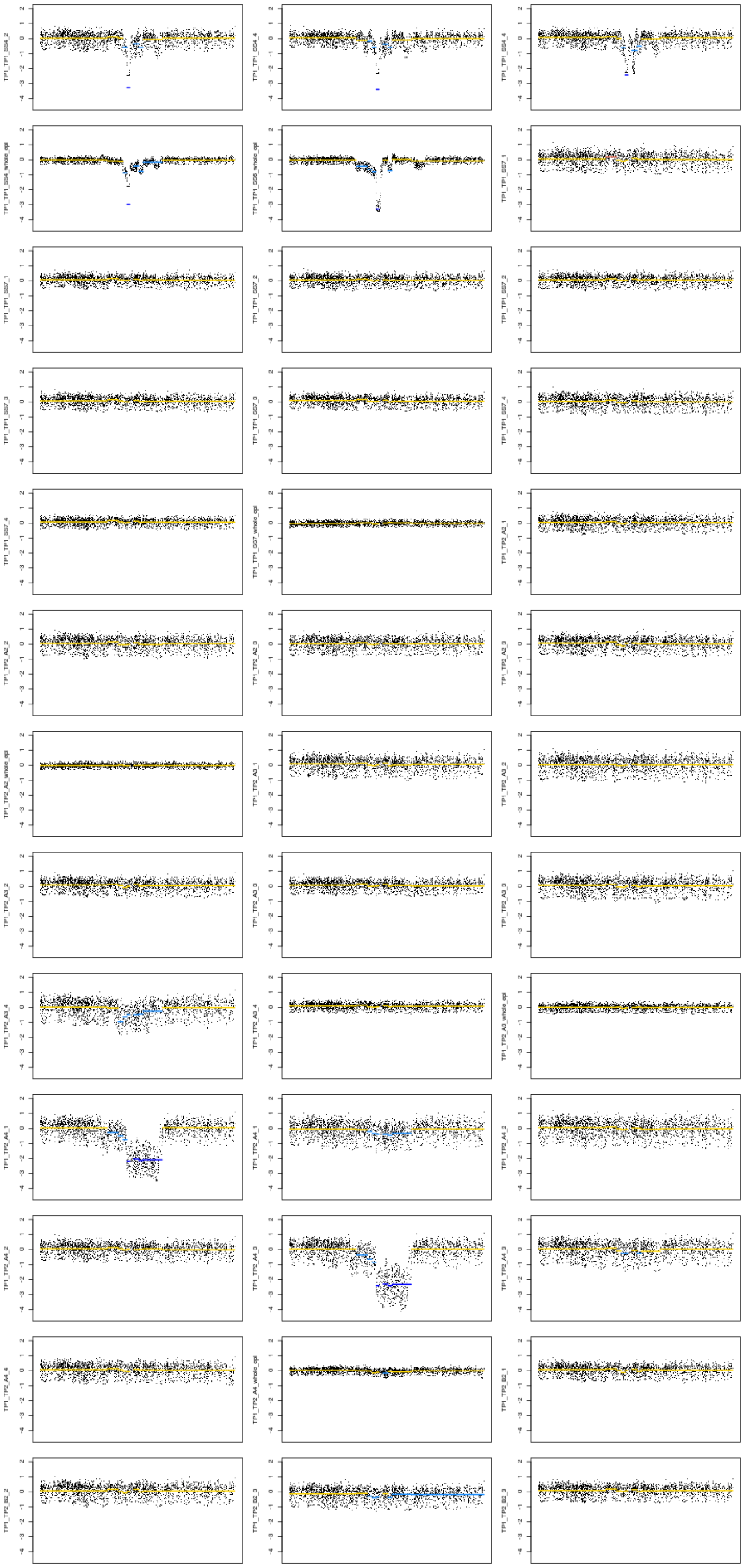

Supplementary Figure 21: Precision segmentation of logR values at the FHIT locus in patient 740-P.

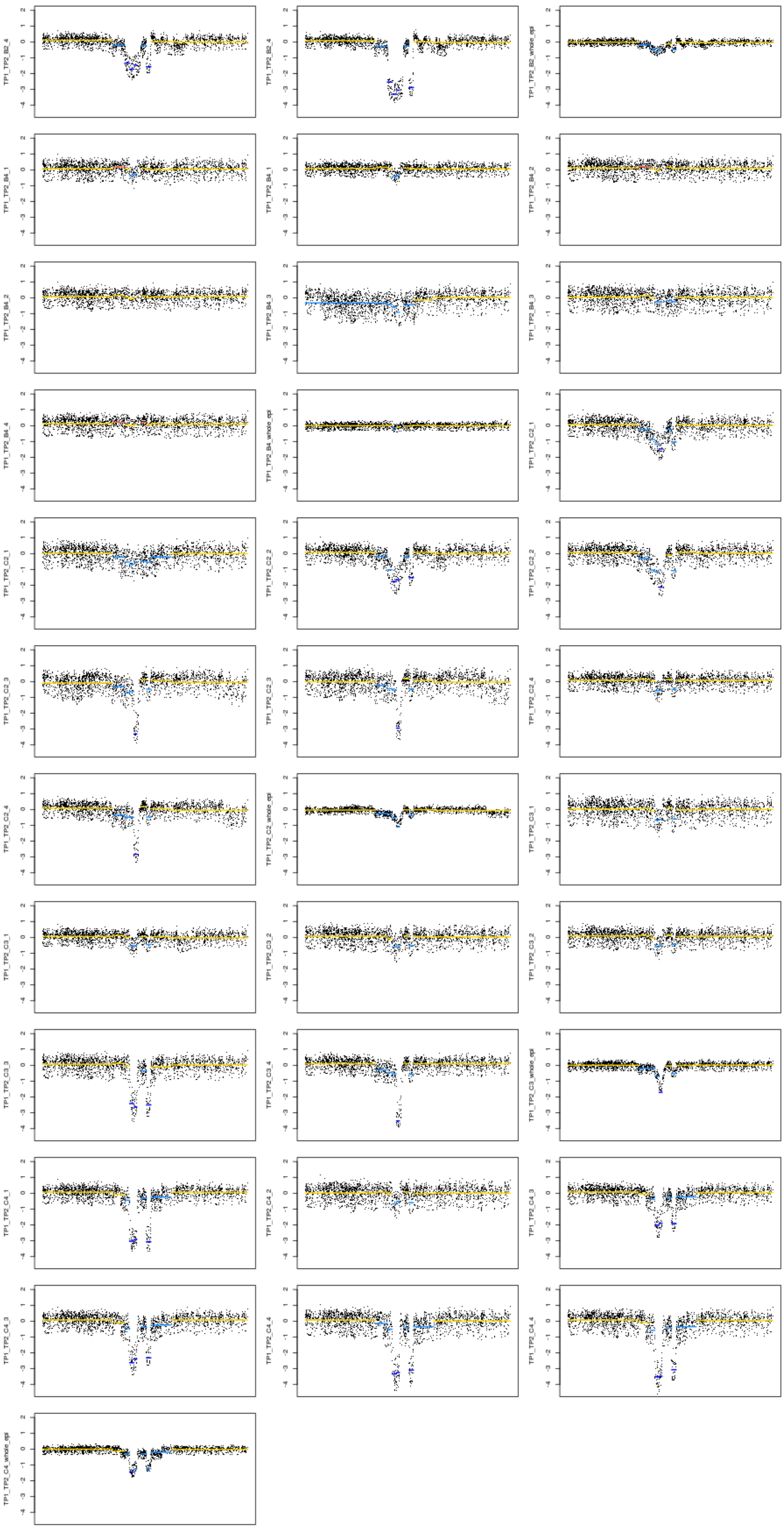

Supplementary Figure 21 (continued).

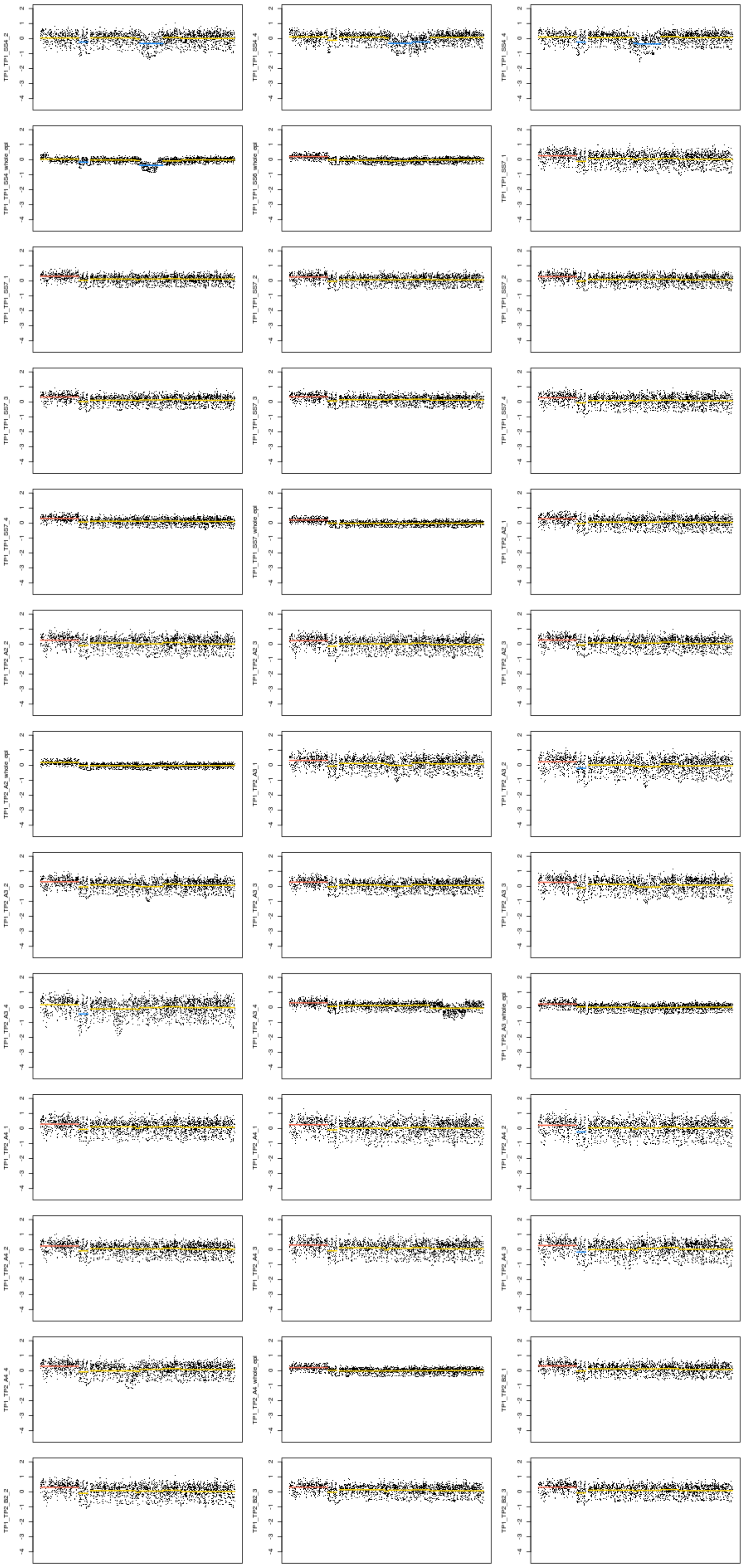

Supplementary Figure 22: Precision segmentation of logR values at the WWOX locus in patient 740-P.

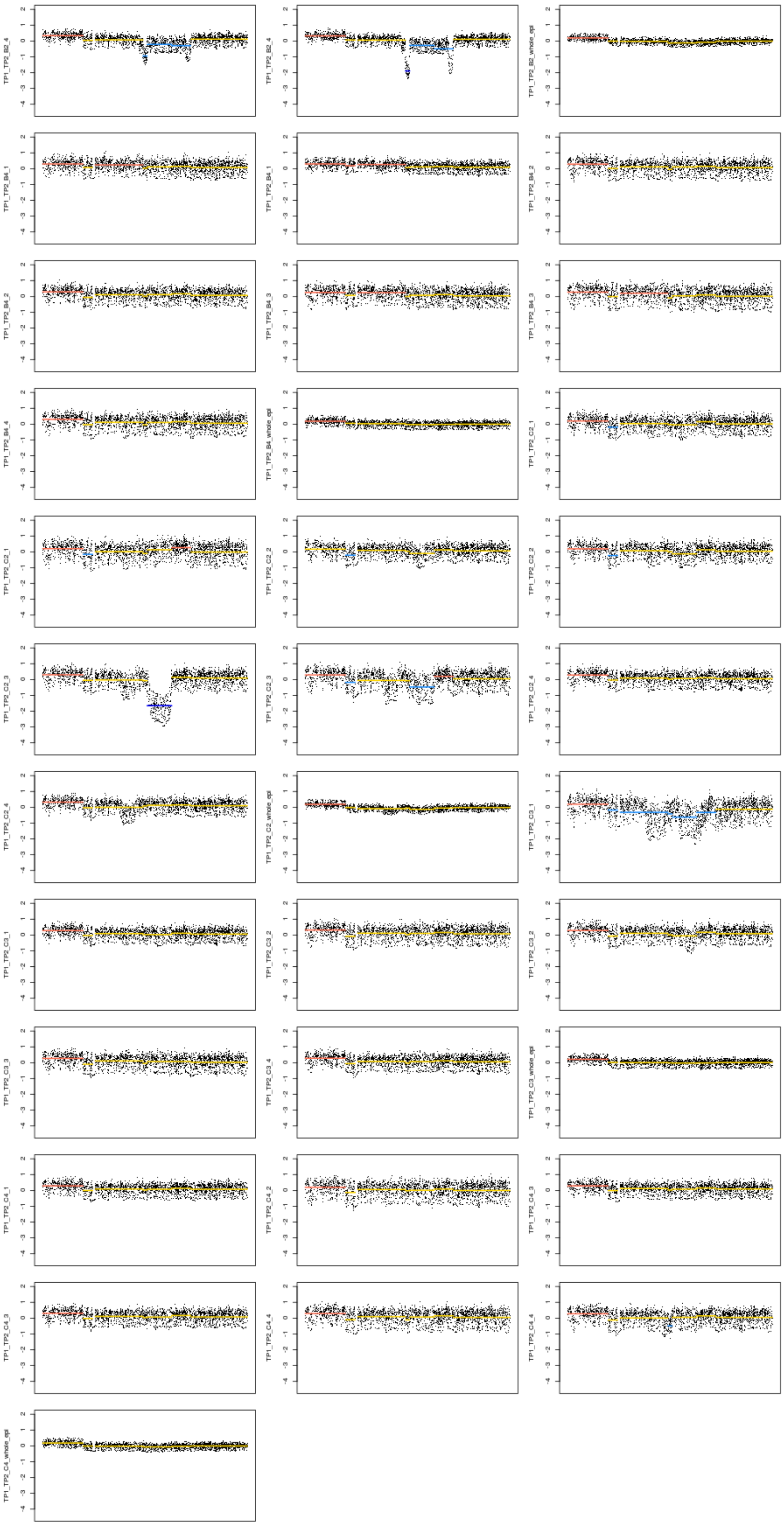

Supplementary Figure 22 (continued).

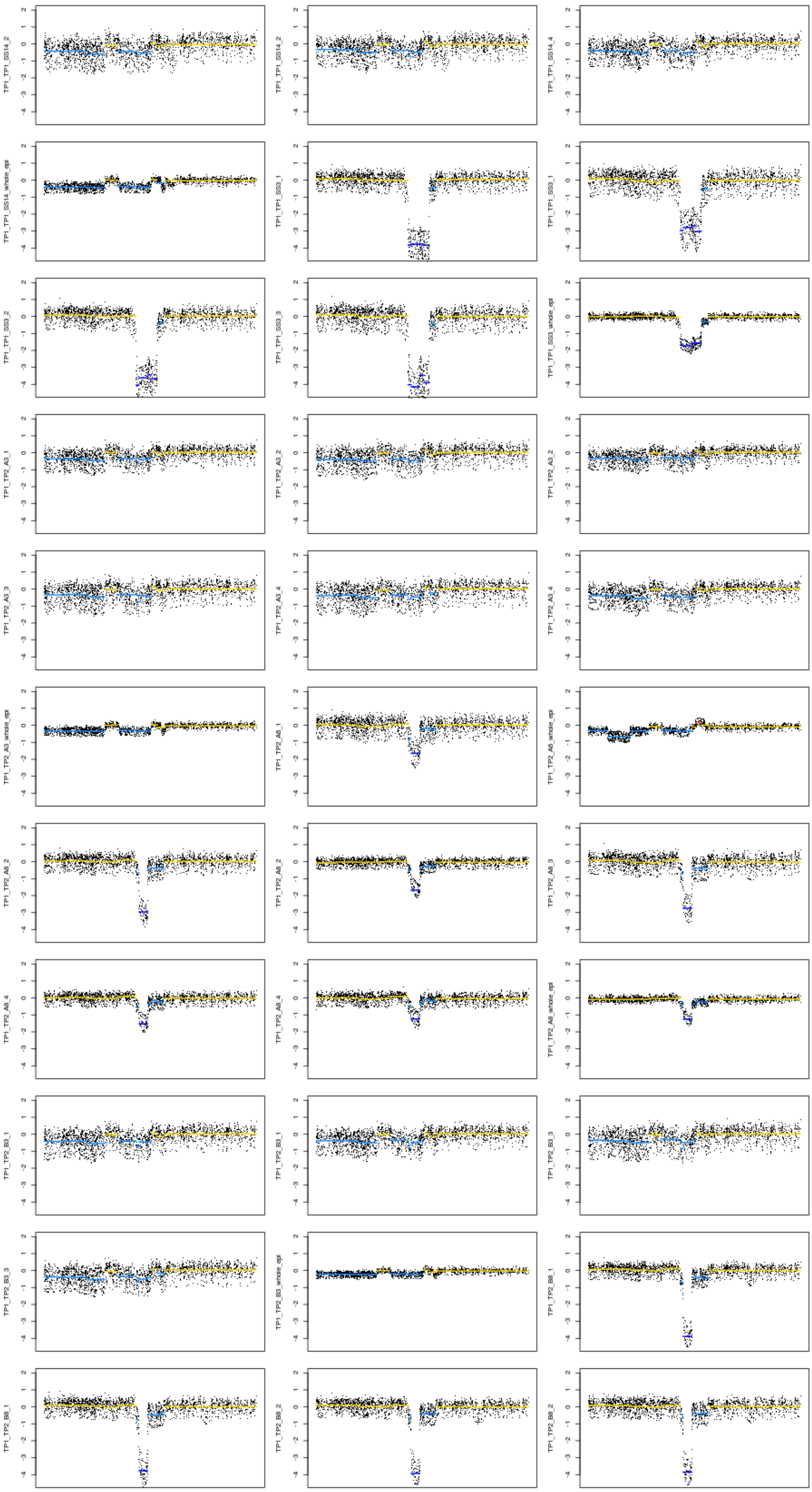

Supplementary Figure 23: Precision segmentation of logR values at the FHIT locus in patient 848-P.

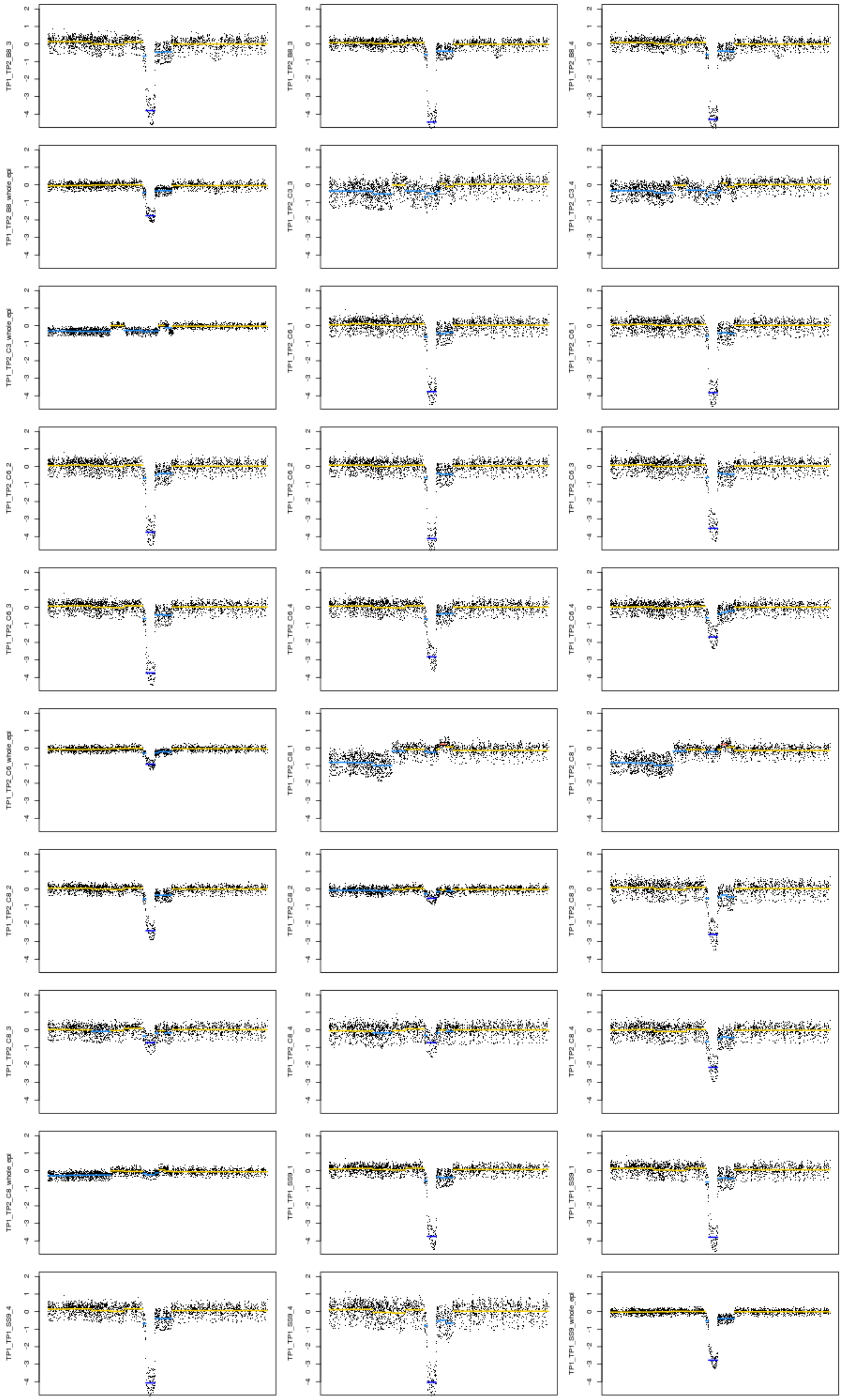

Supplementary Figure 23 (continued).

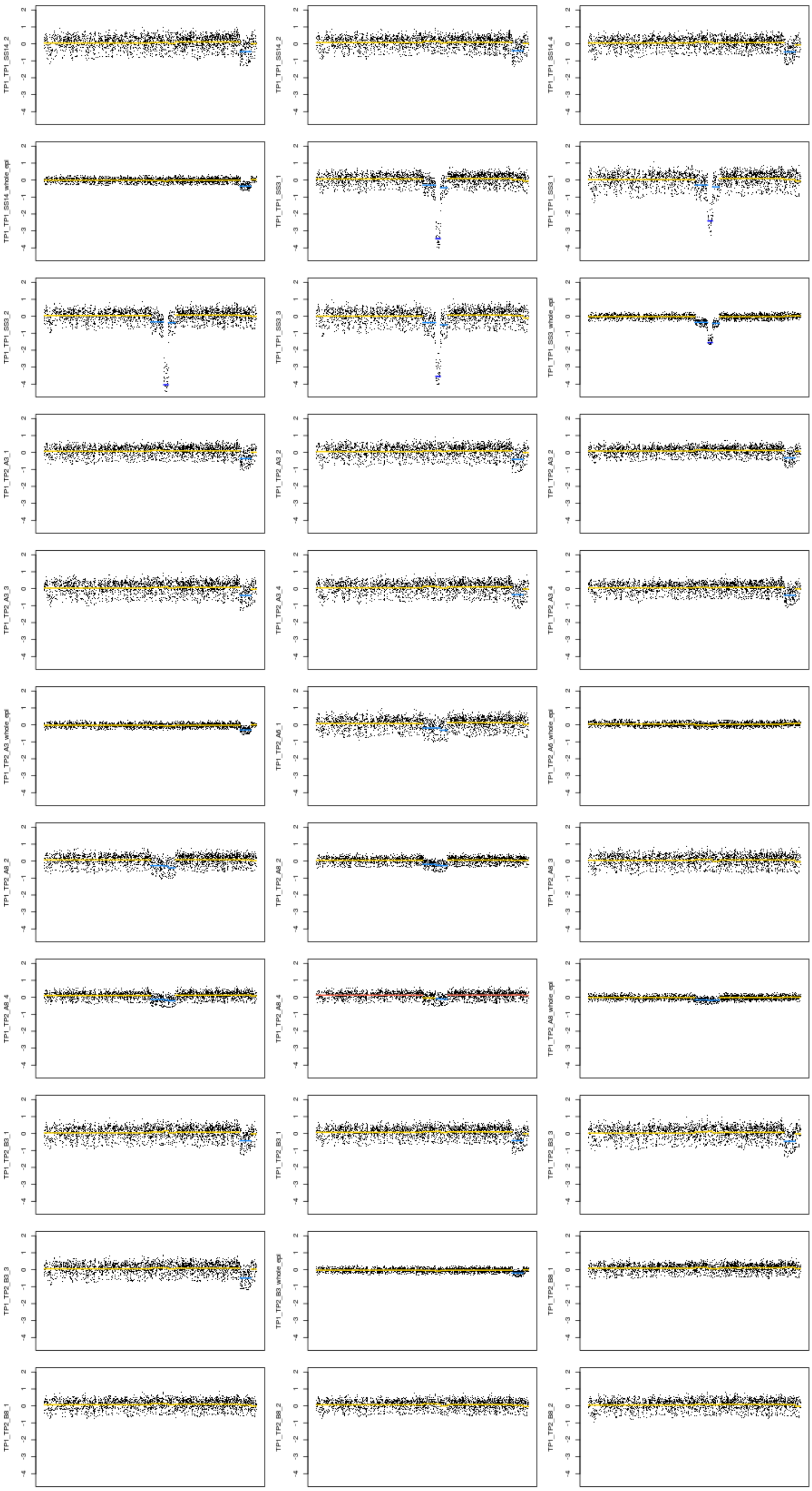

Supplementary Figure 24: Precision segmentation of logR values at the WWOX locus in patient 848-P.

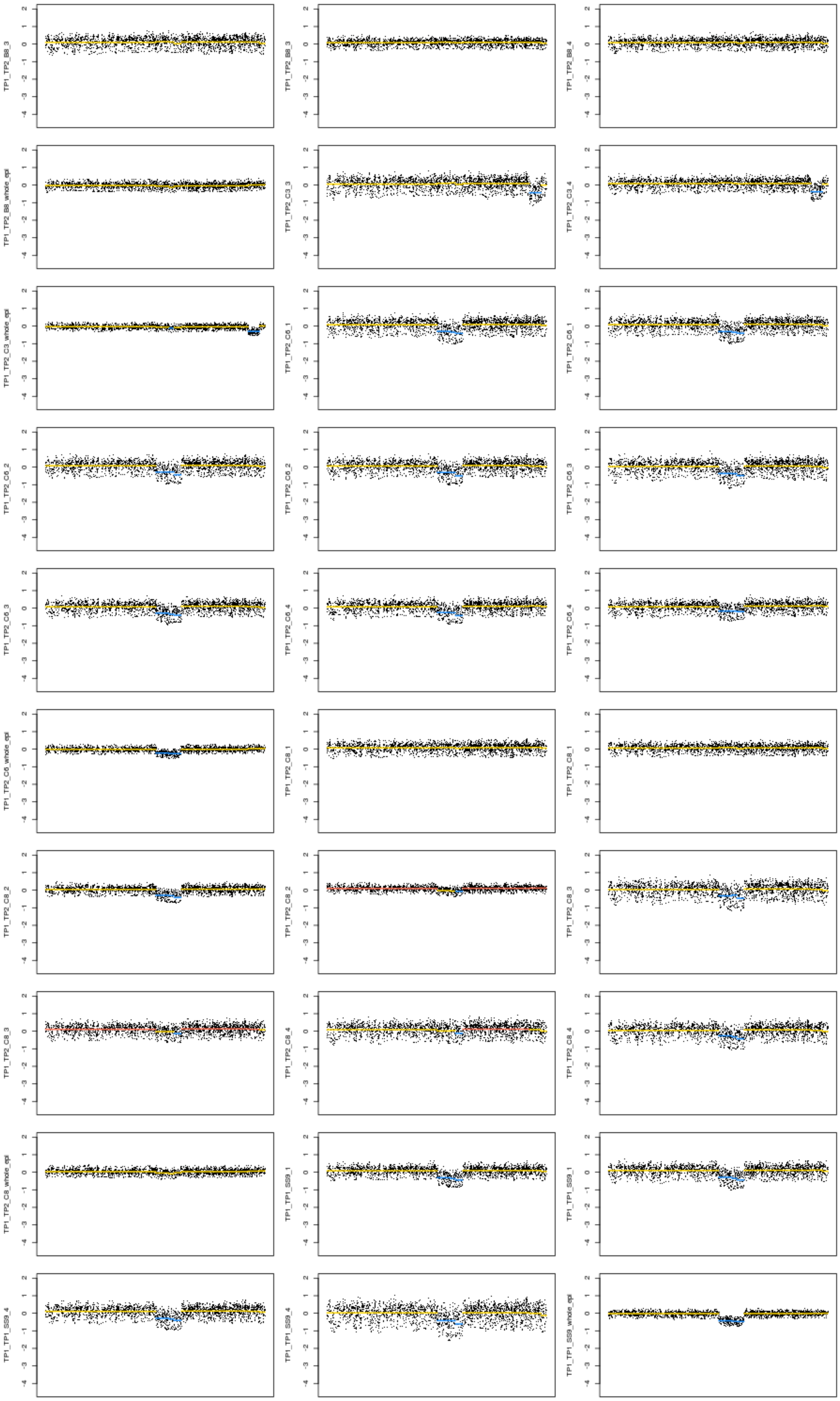

Supplementary Figure 24 (continued).

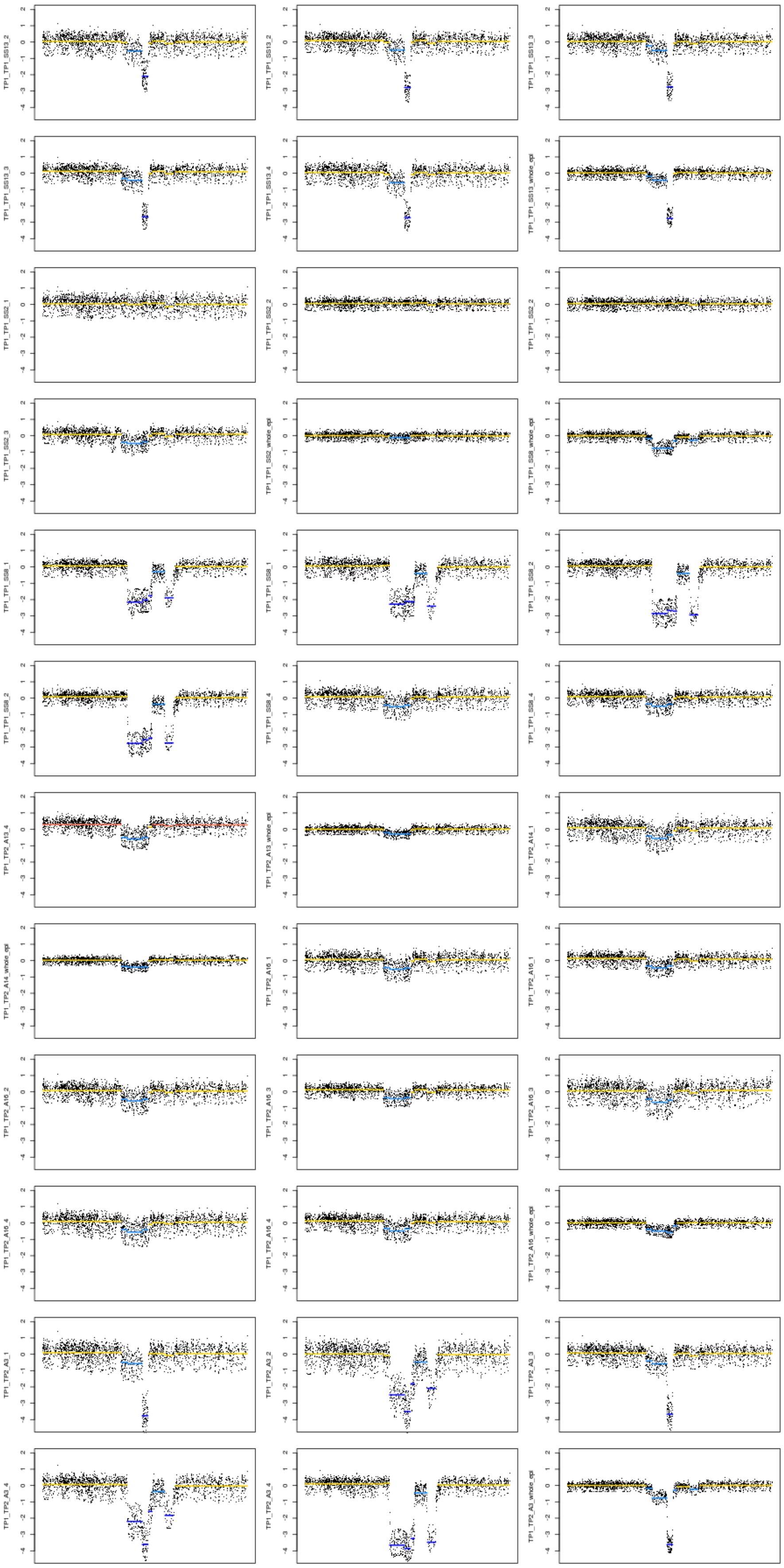

Supplementary Figure 25: Precision segmentation of logR values at the FHIT locus in patient 852-P.

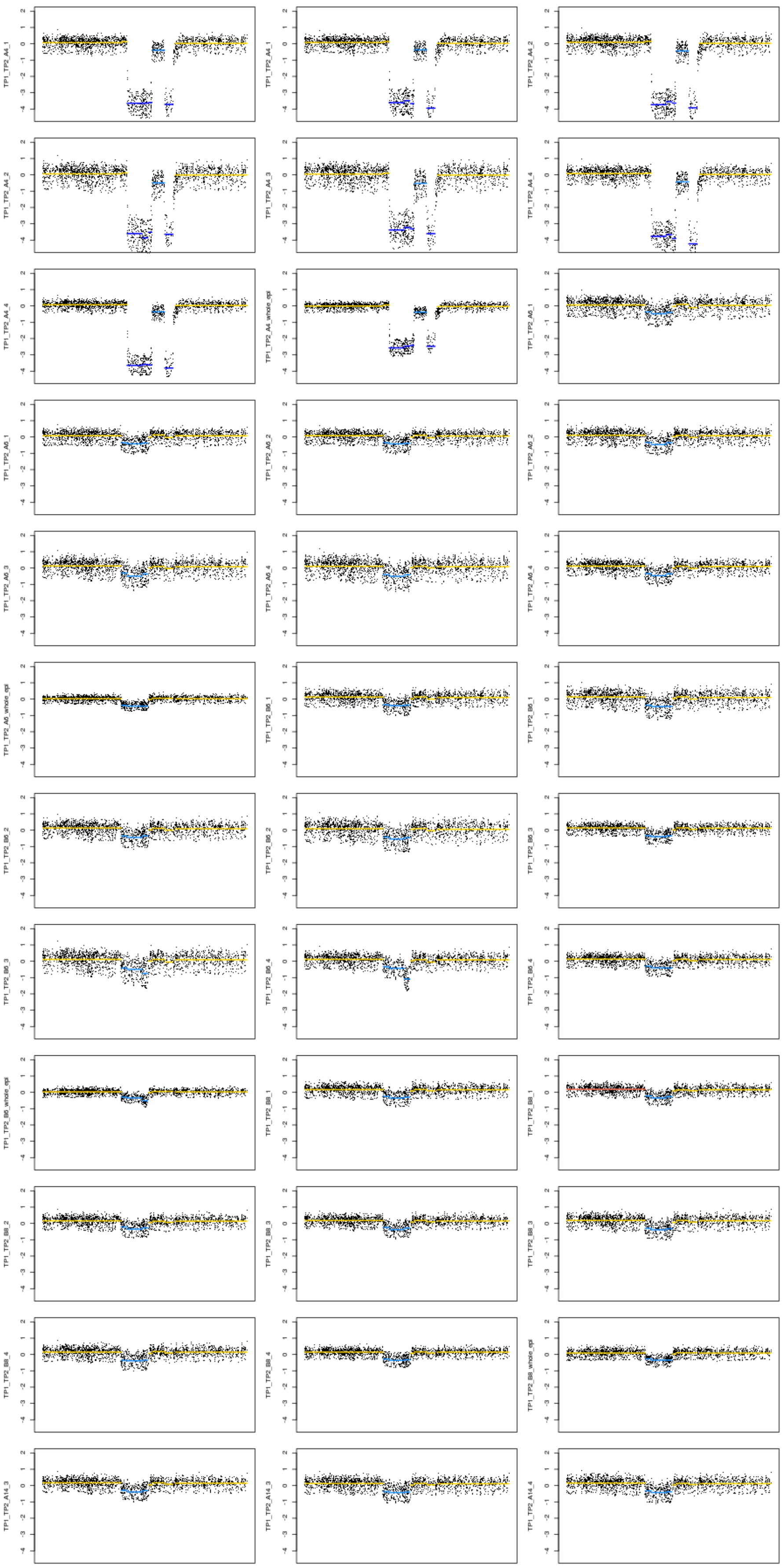

Supplementary Figure 25 (continued)

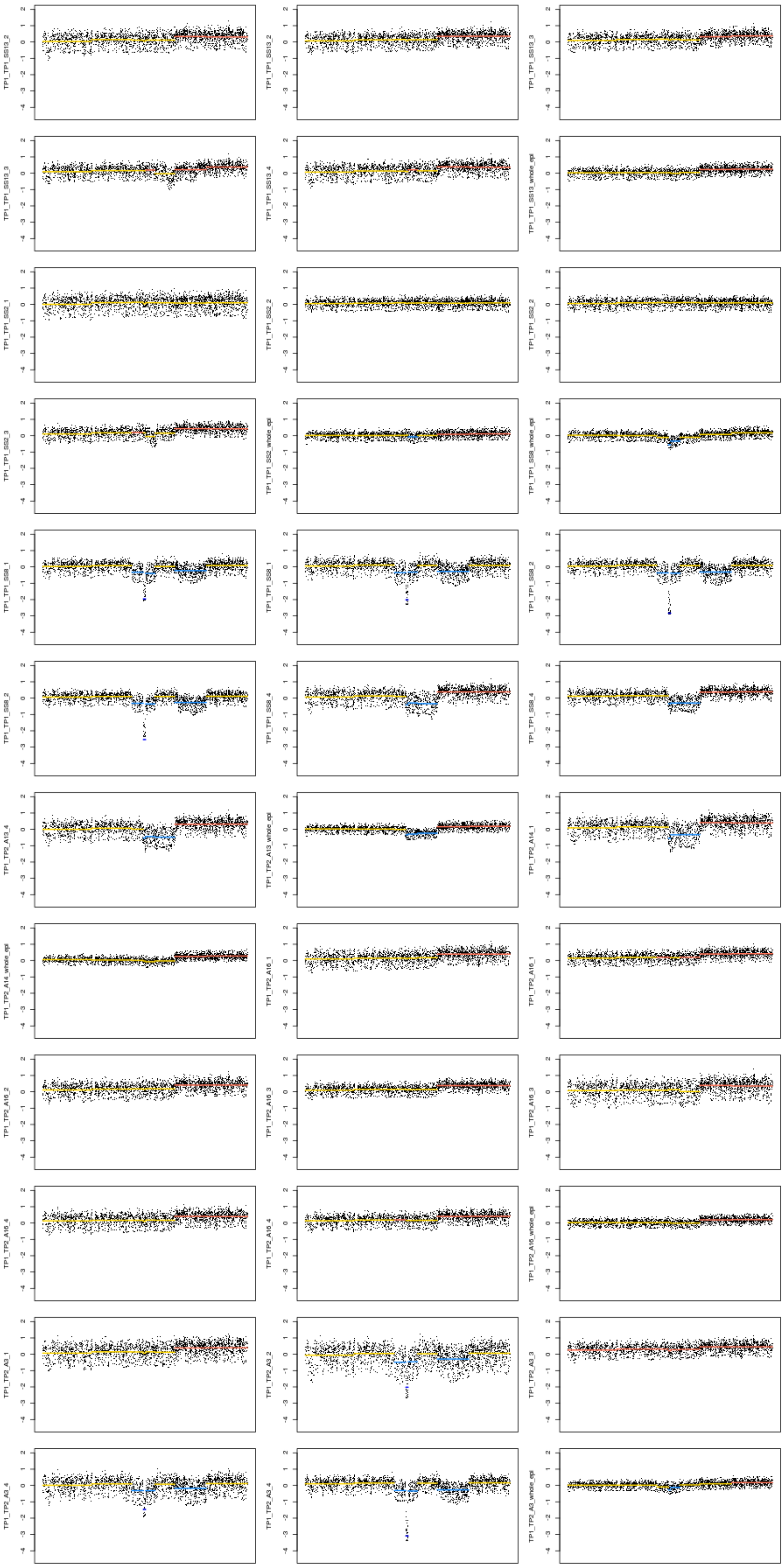

Supplementary Figure 26: Precision segmentation of logR values at the WWOX locus in patient 852-P.

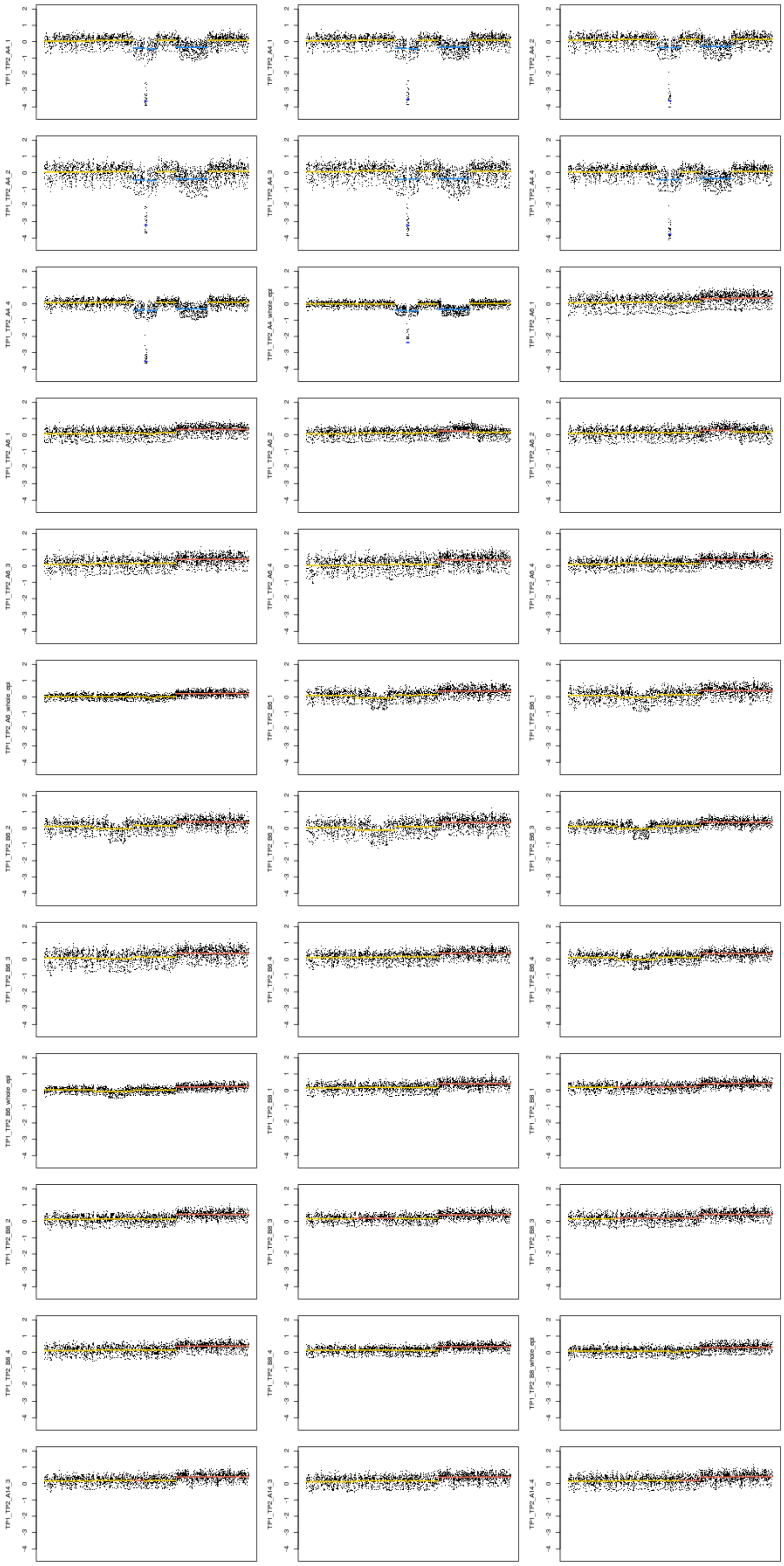

Supplementary Figure 26 (continued).

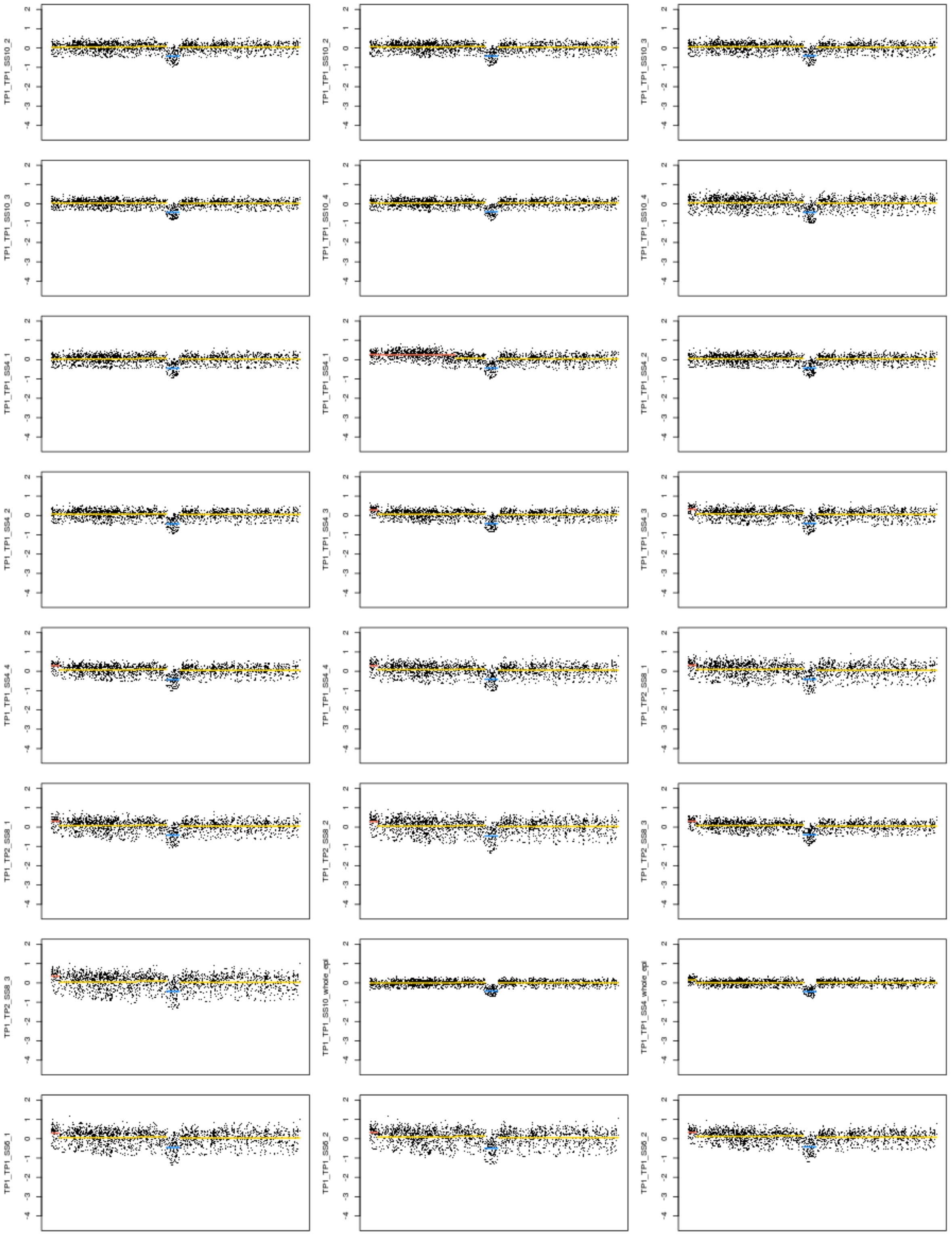

Supplementary Figure 27: Precision segmentation of logR values at the FHIT locus in patient 911-NP.

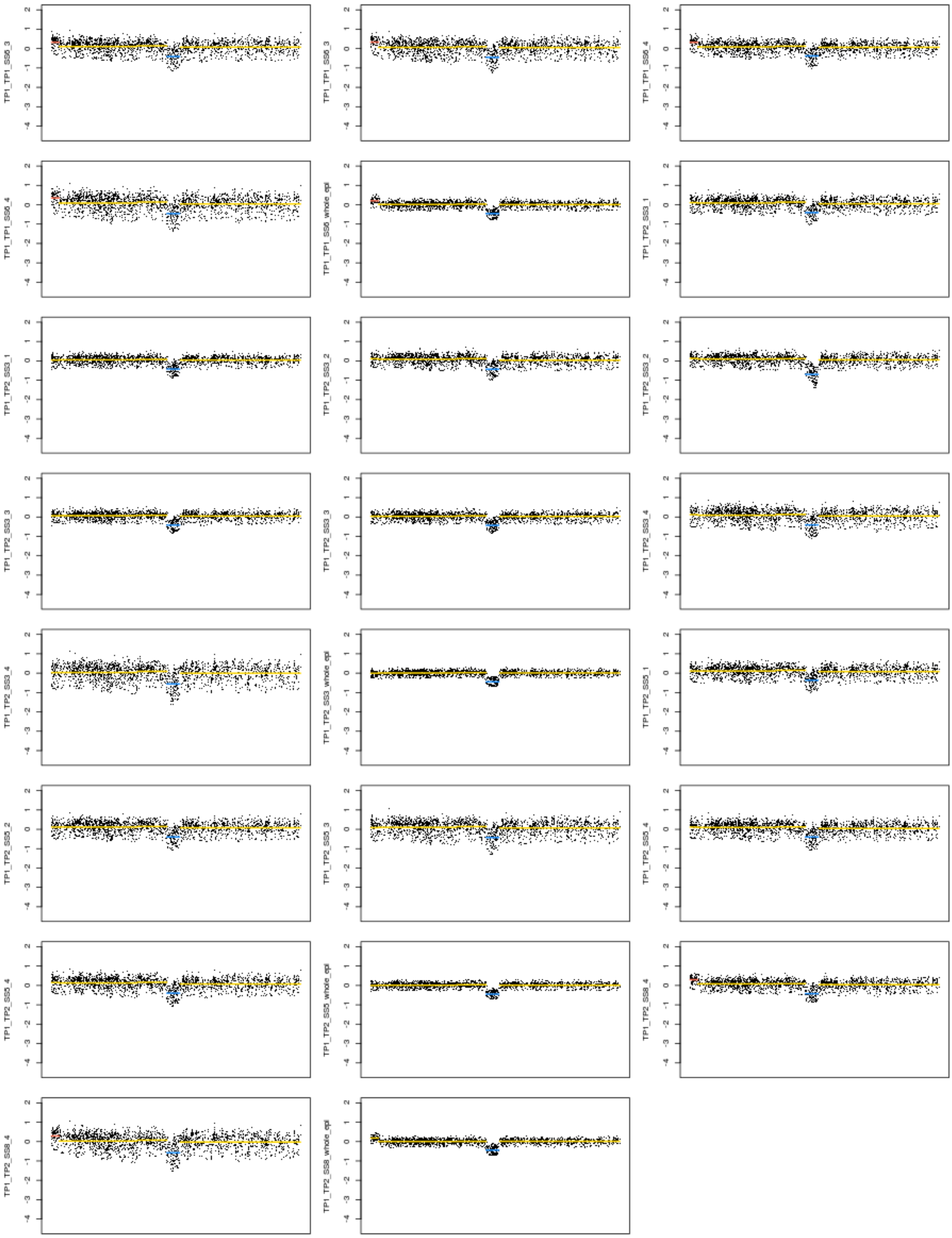

Supplementary Figure 27 (continued).

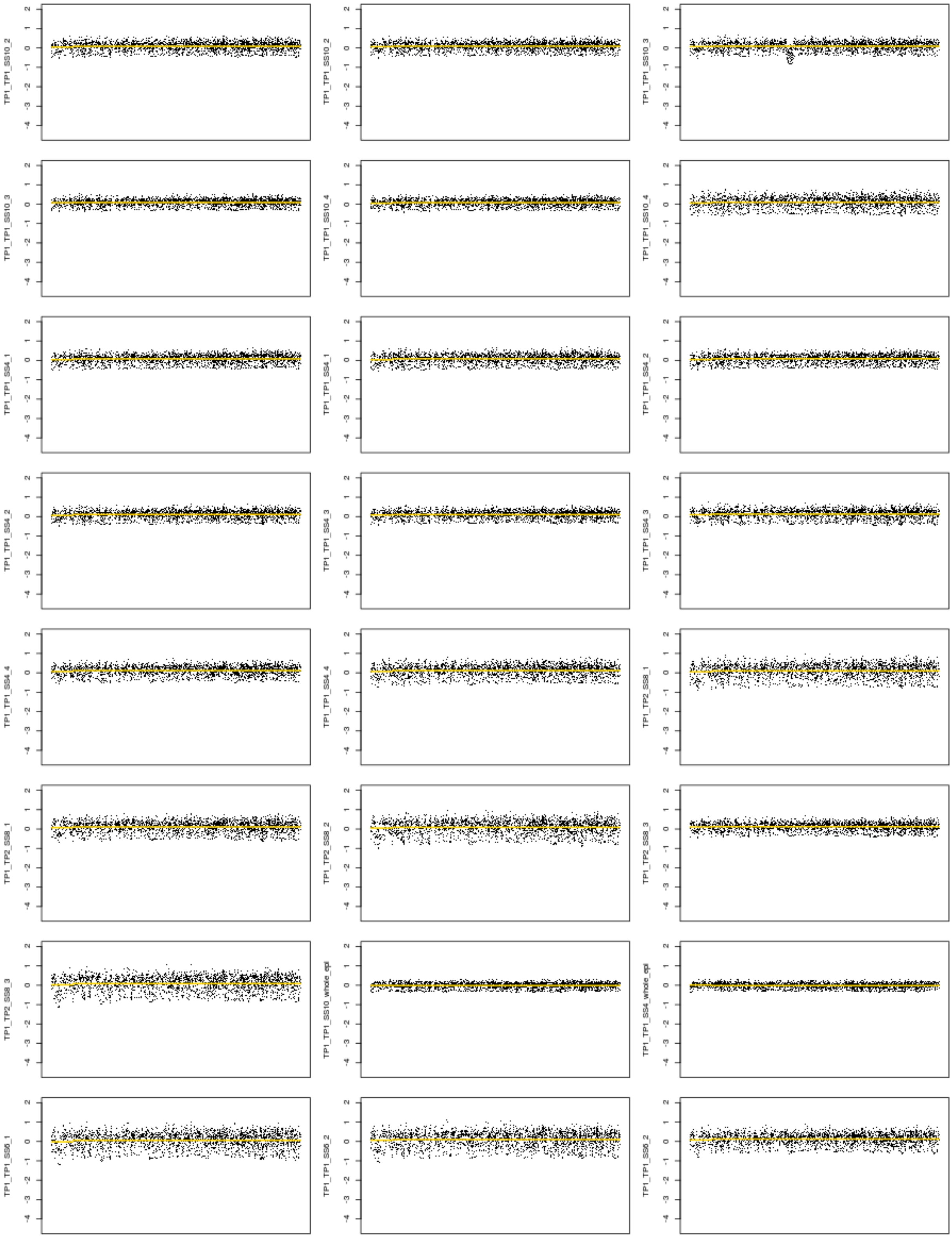

Supplementary Figure 28: Precision segmentation of logR values at the WWOX locus in patient 911-NP.

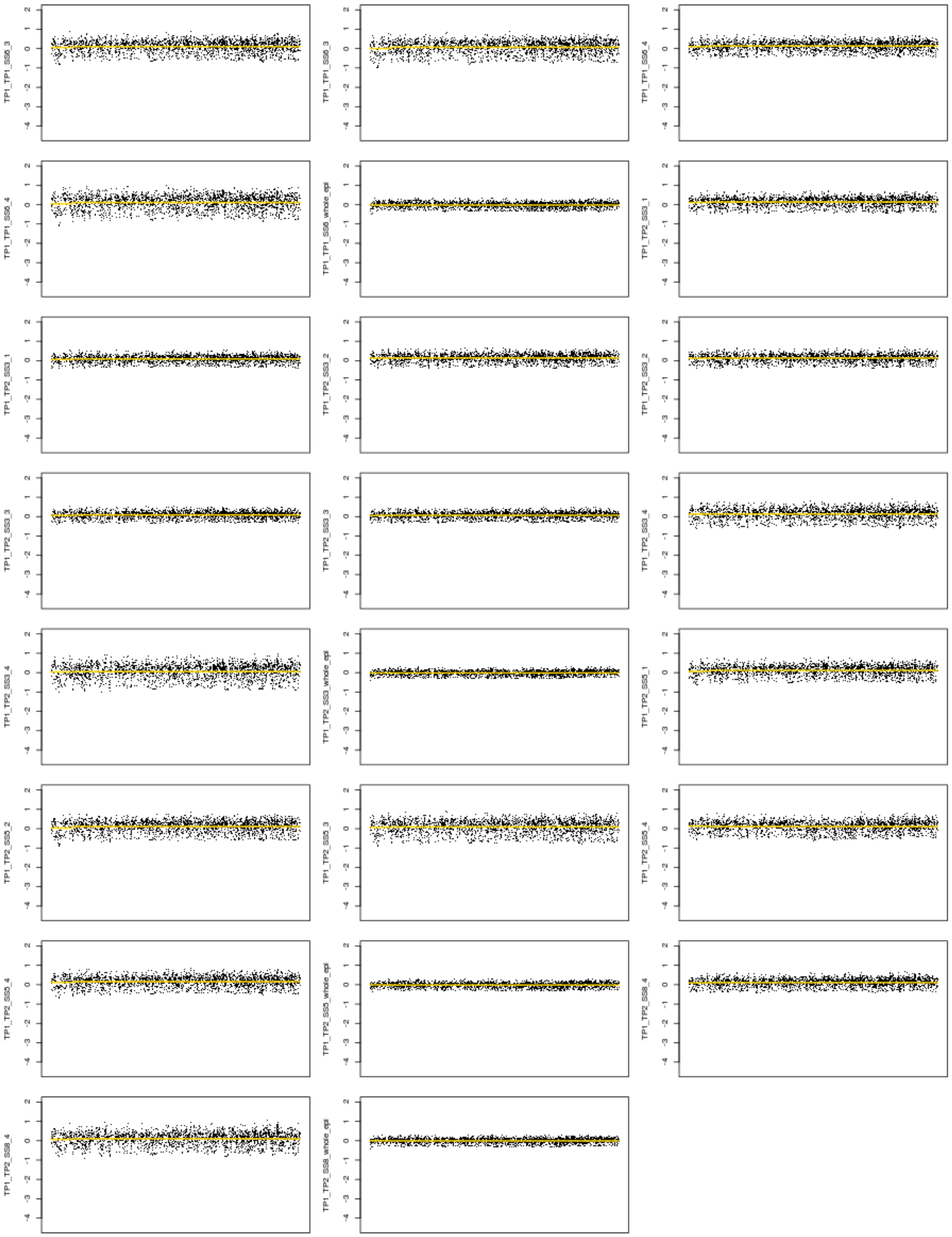

Supplementary Figure 28 (continued).

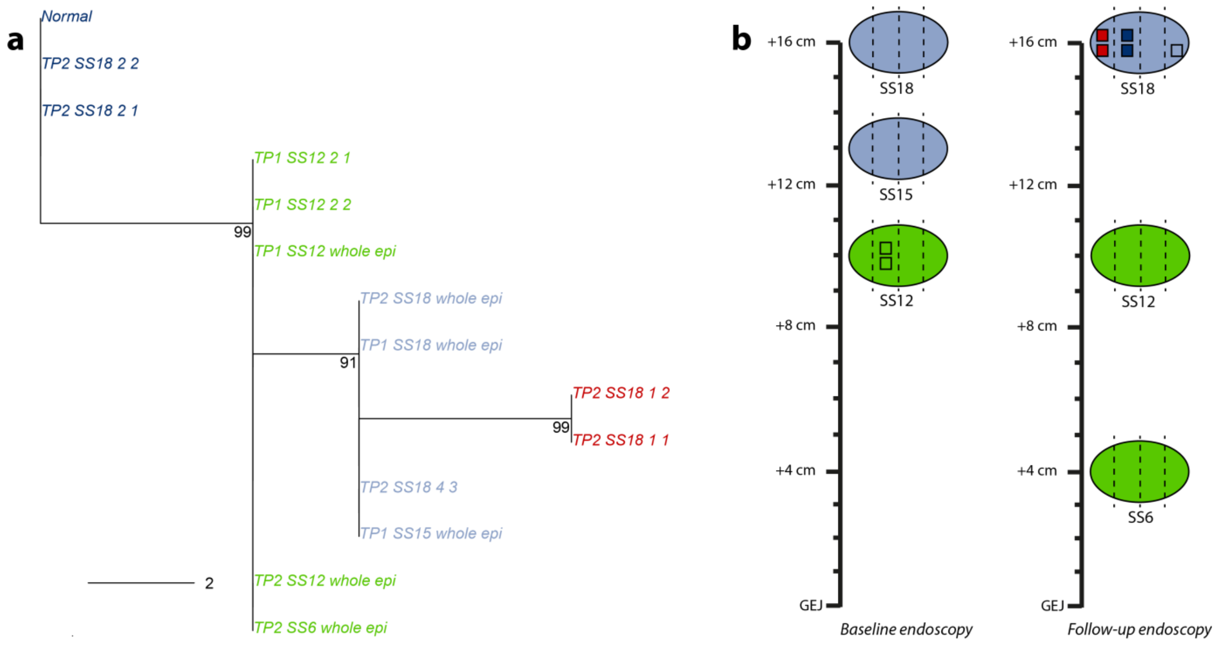

Supplementary Figure 29: Patient 256-NP phylogeographic map. a) Phylogenetic tree. Presence or absence of breakpoints are the basic genetic events used to reconstruct the tree using a parsimony algorithm. b) Baseline and follow-up endoscopy maps. Biopsies are indicated at sampling location by ovals, split into 4 baguette section labelled 1 to 4 from left to right. Individual crypts for which we could produce copy number profiles are indicated by squares in the relevant baguette section of the biopsy from which they originate. Colours were attributed via principal component analysis based on the presence/absence matrix of breakpoints per sample, mapping the first three components to red, green and blue, respectively. Patient 256 is a non-progressor.

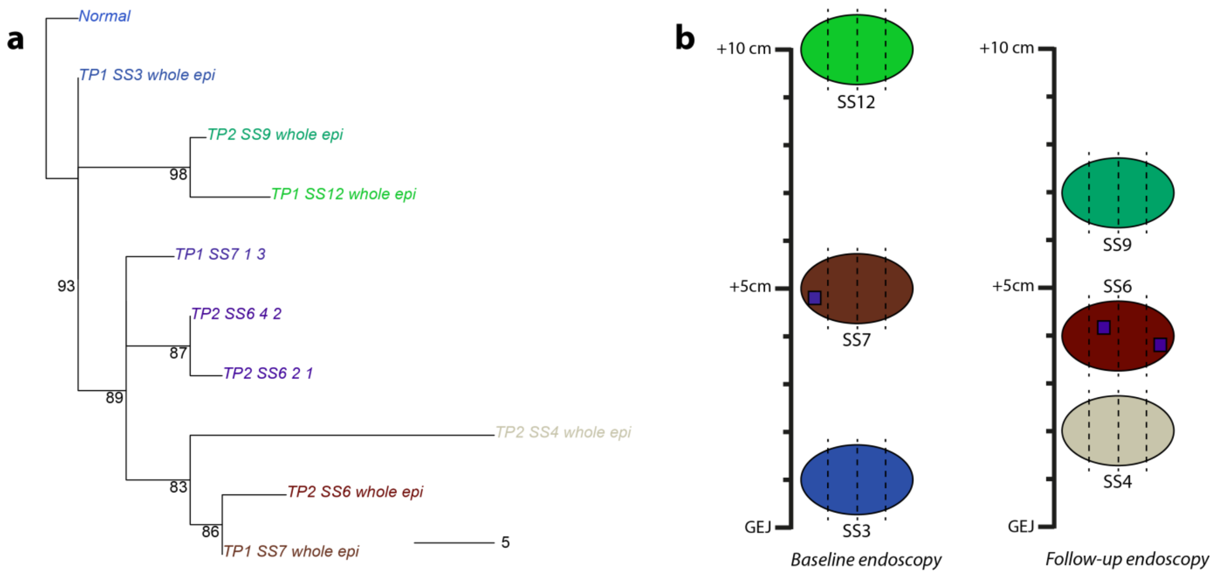

Supplementary Figure 30: Patient 437-NP phylogeographic map. a) Phylogenetic tree. b) Baseline and follow-up endoscopy maps. Patient 437 is a non-progressor.

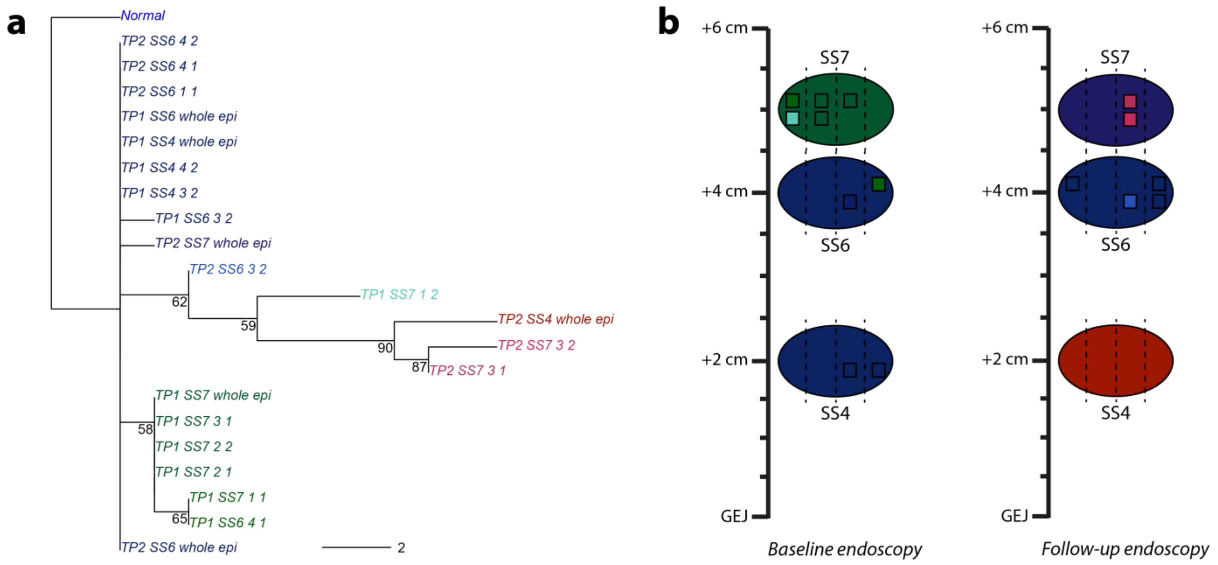

Supplementary Figure 31: Patient 451-NP phylogeographic map. a) Phylogenetic tree. b) Baseline and follow-up endoscopy maps. Patient 451 is a non-progressor.

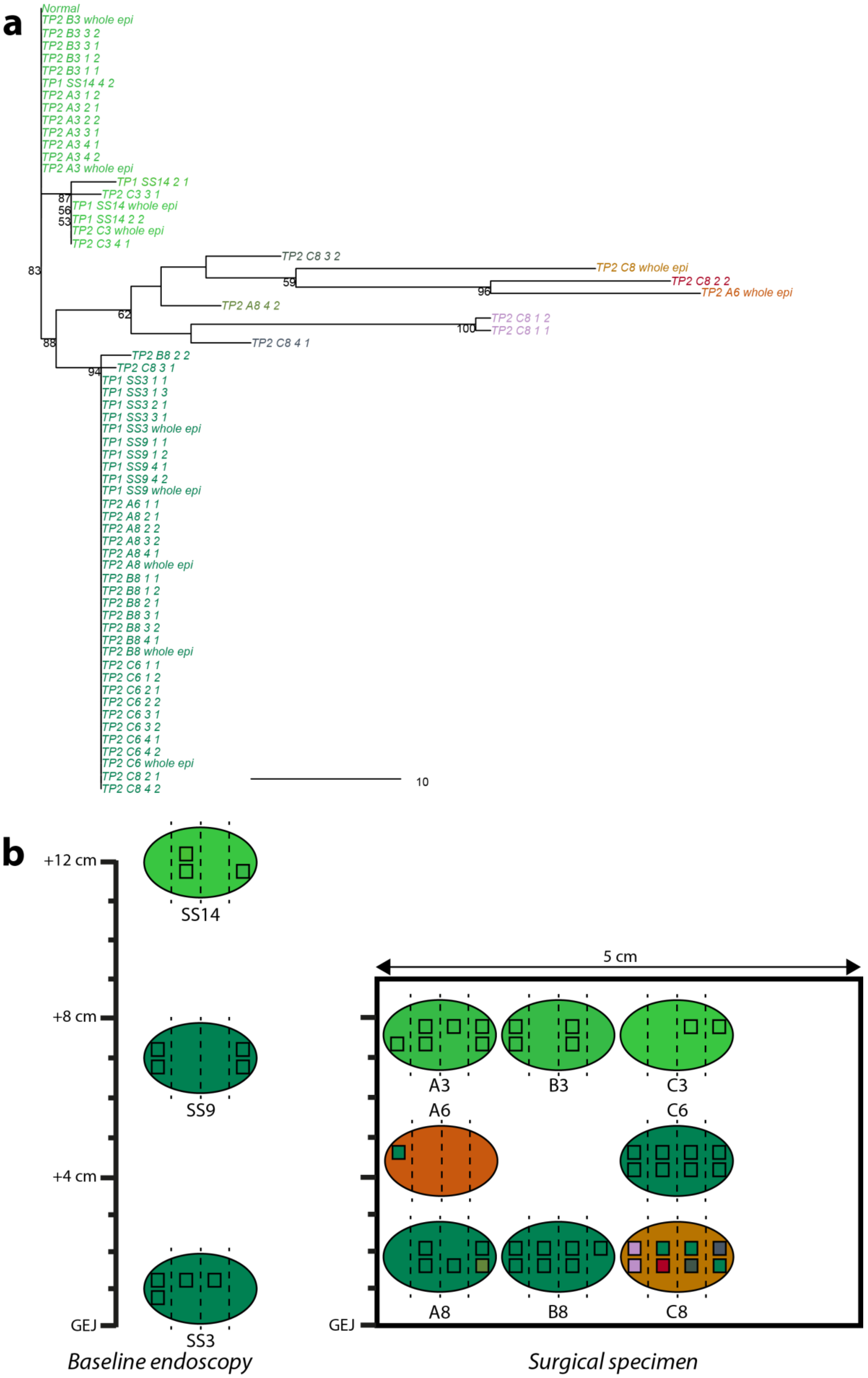

Supplementary Figure 32: Patient 848-P phylogeographic map. a) Phylogenetic tree. b) Baseline endoscopy and surgical resection maps. Patient 848 is a progressor.

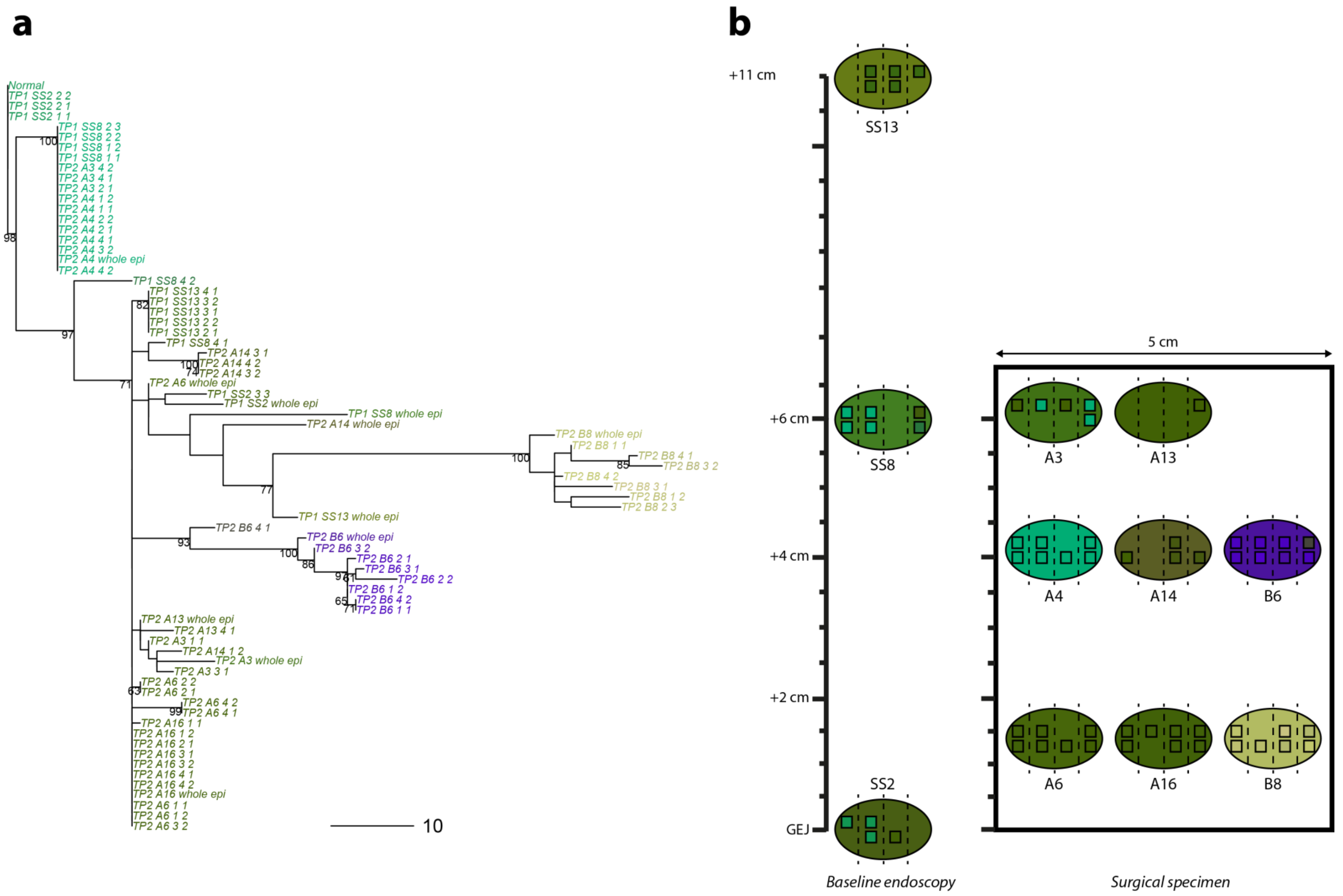

Supplementary Figure 33: Patient 852-P phylogeographic map. a) Phylogenetic tree. b) Baseline endoscopy and surgical resection maps. Patient 852 is a progressor.

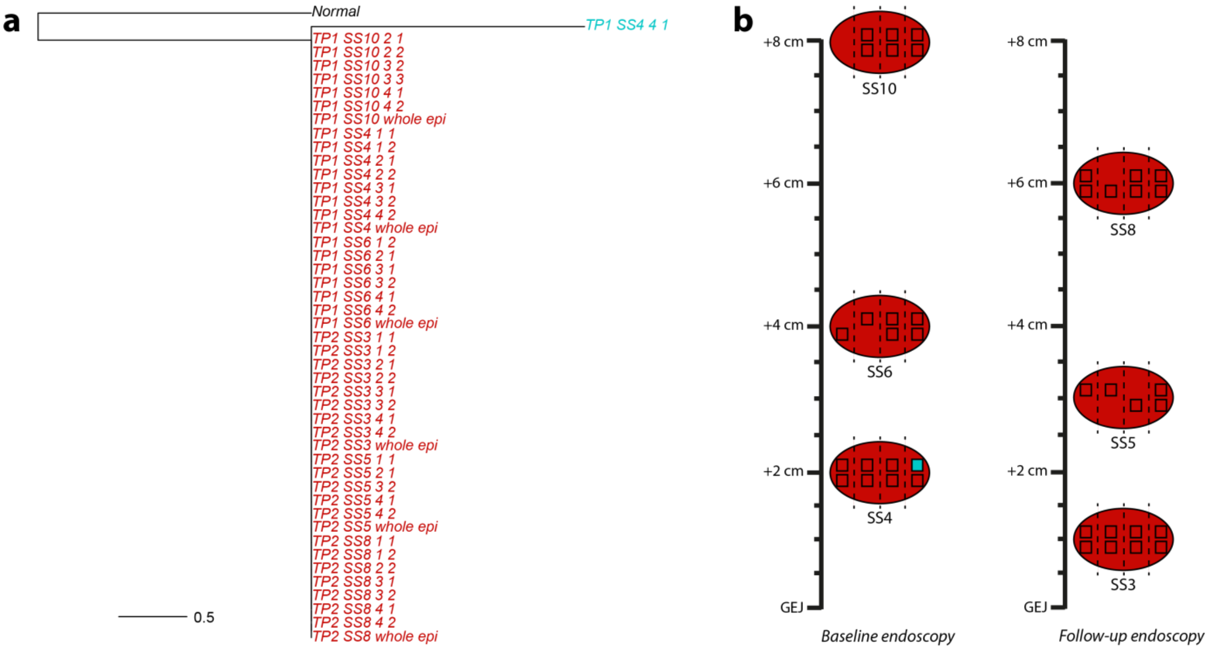

Supplementary Figure 34: Patient 911-NP phylogeographic map. a) Phylogenetic tree. b) Baseline and follow-up endoscopy maps. Patient 911 is a non-progressor.

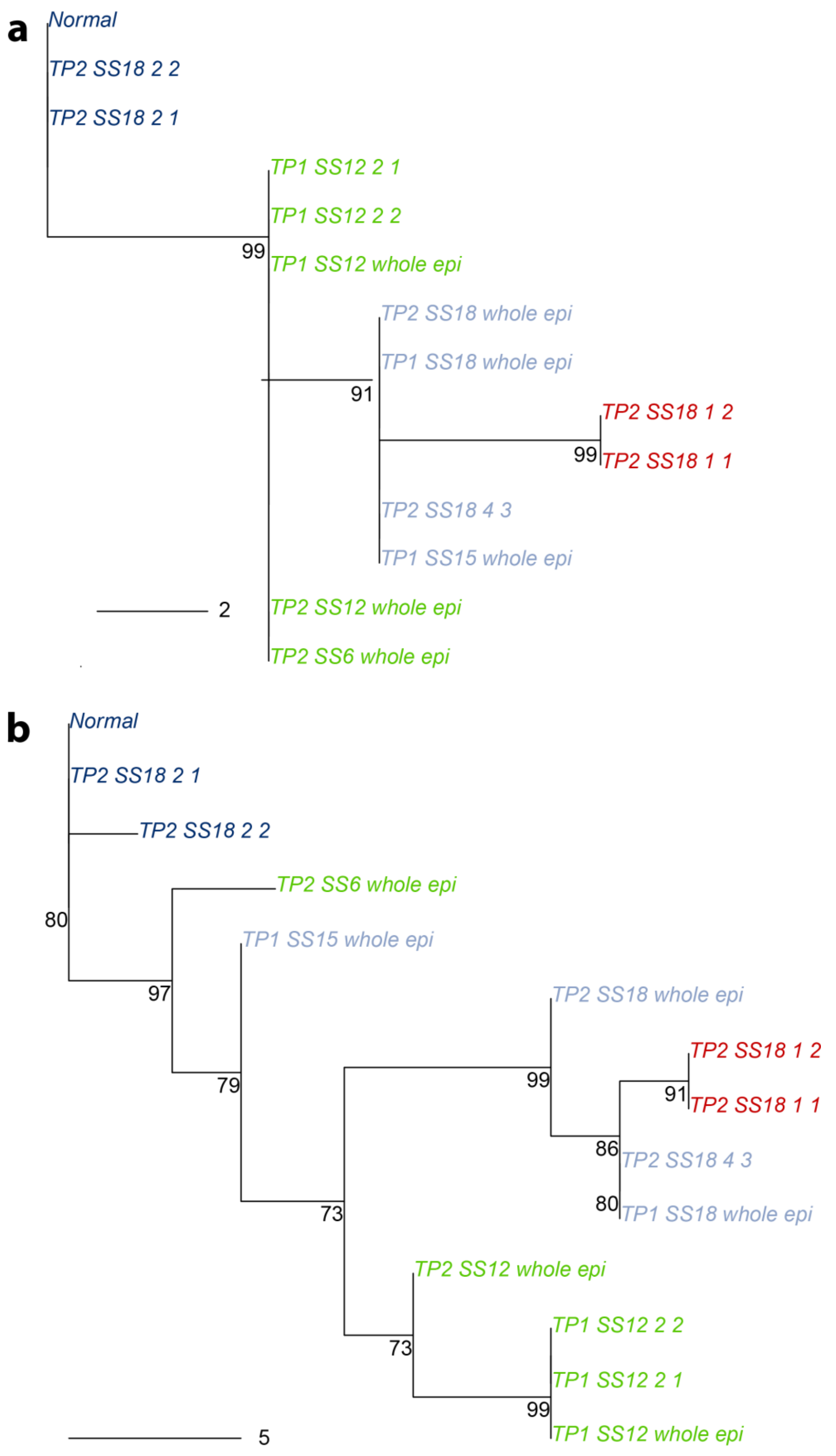

Supplementary Figure 35: Whole genome and fragile site trees for patient 256-NP. a) Whole genome tree. b) Fragile site tree. The fragile site tree was obtained similarly to the whole-genome tree based on fragile site breakpoints. Tip colours are the same as in the whole genome tree.

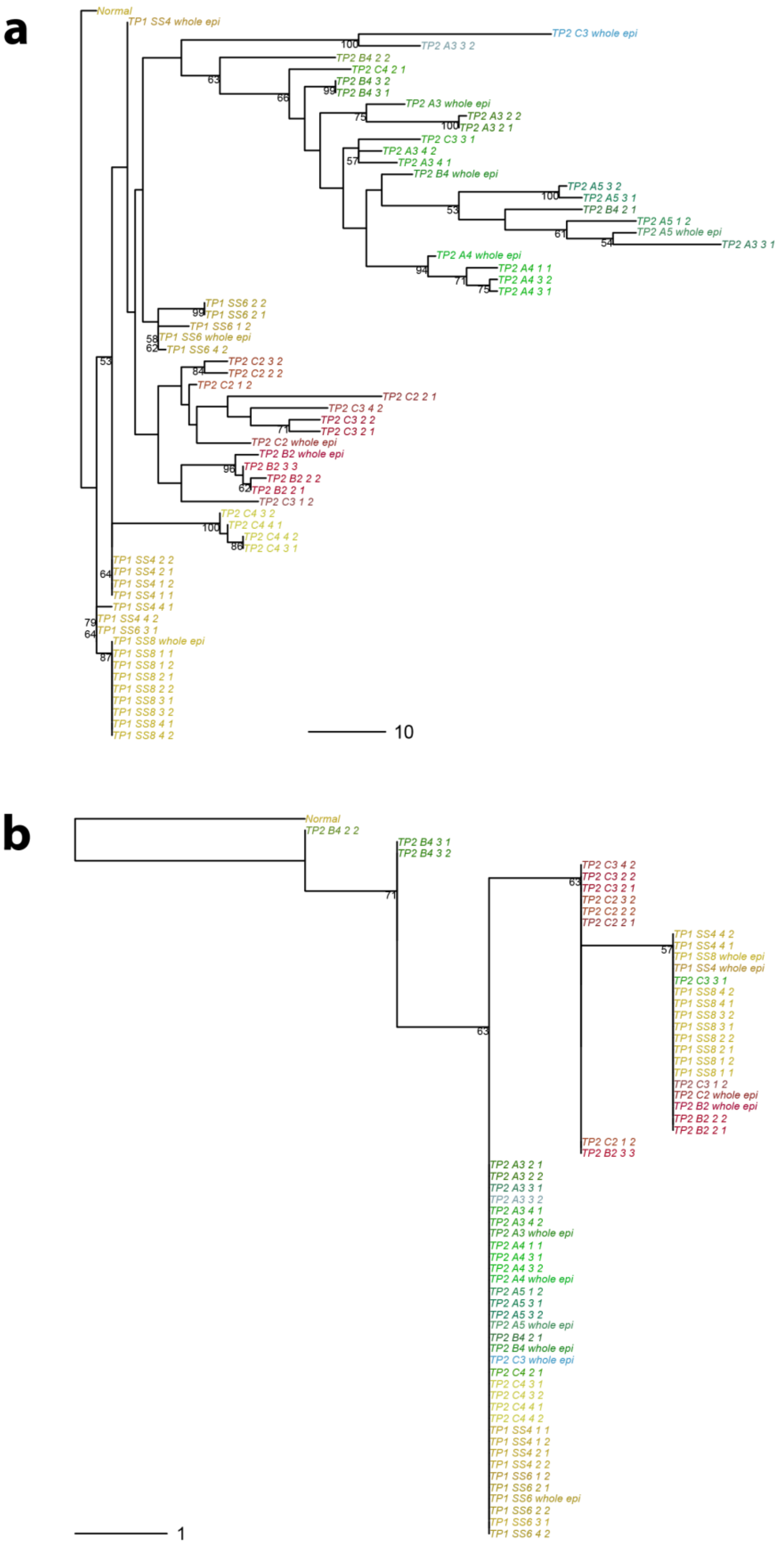

Supplementary Figure 36: Whole genome and fragile site trees for patient 391-P. a) Whole genome tree. b) Fragile site tree. The fragile site tree was obtained similarly to the whole-genome tree based on fragile site breakpoints. Tip colours are the same as in the whole genome tree.

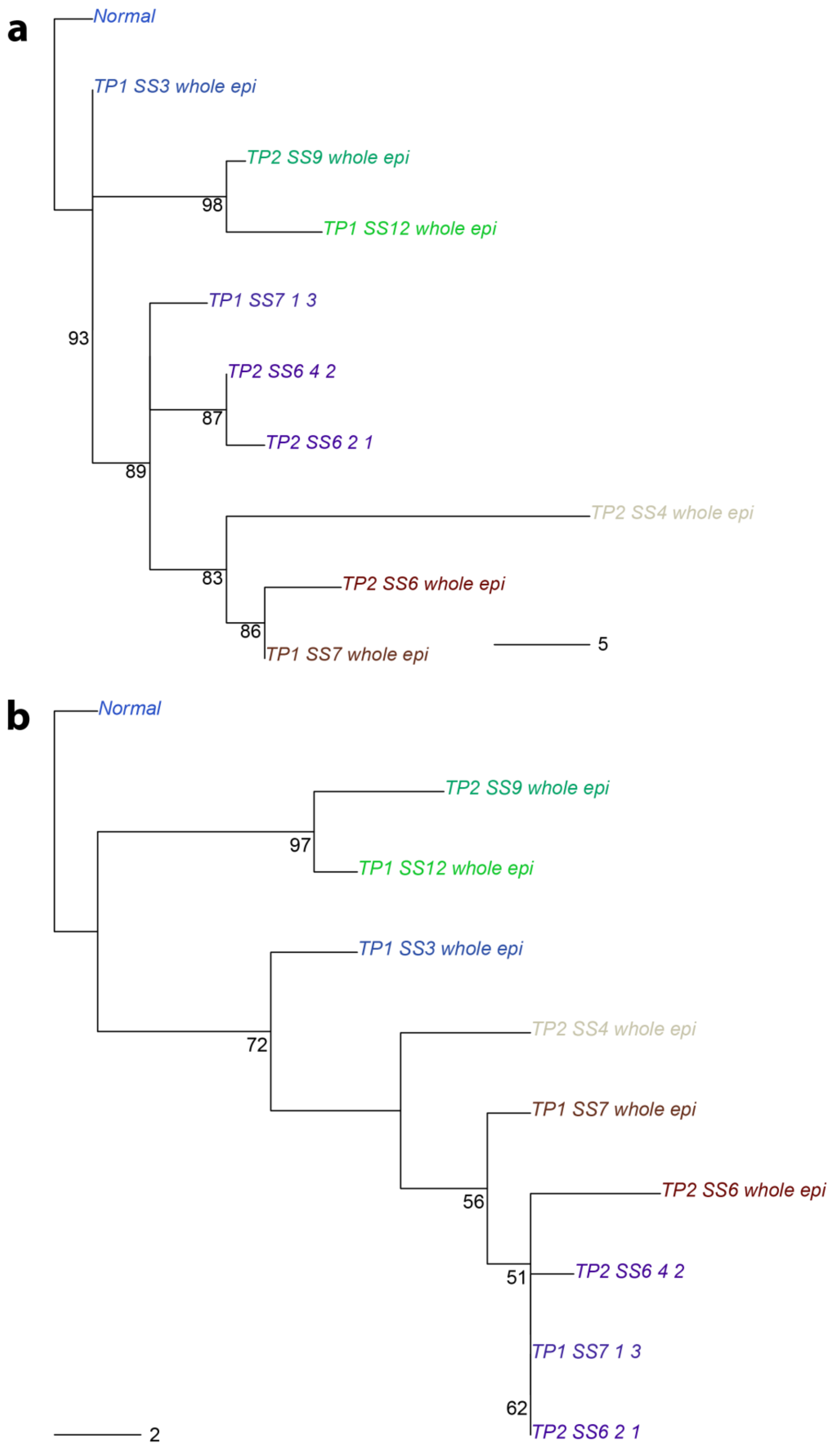

Supplementary Figure 37: Whole genome and fragile site trees for patient 437-NP. a) Whole genome tree. b) Fragile site tree. The fragile site tree was obtained similarly to the whole-genome tree based on fragile site breakpoints. Tip colours are the same as in the whole genome tree.

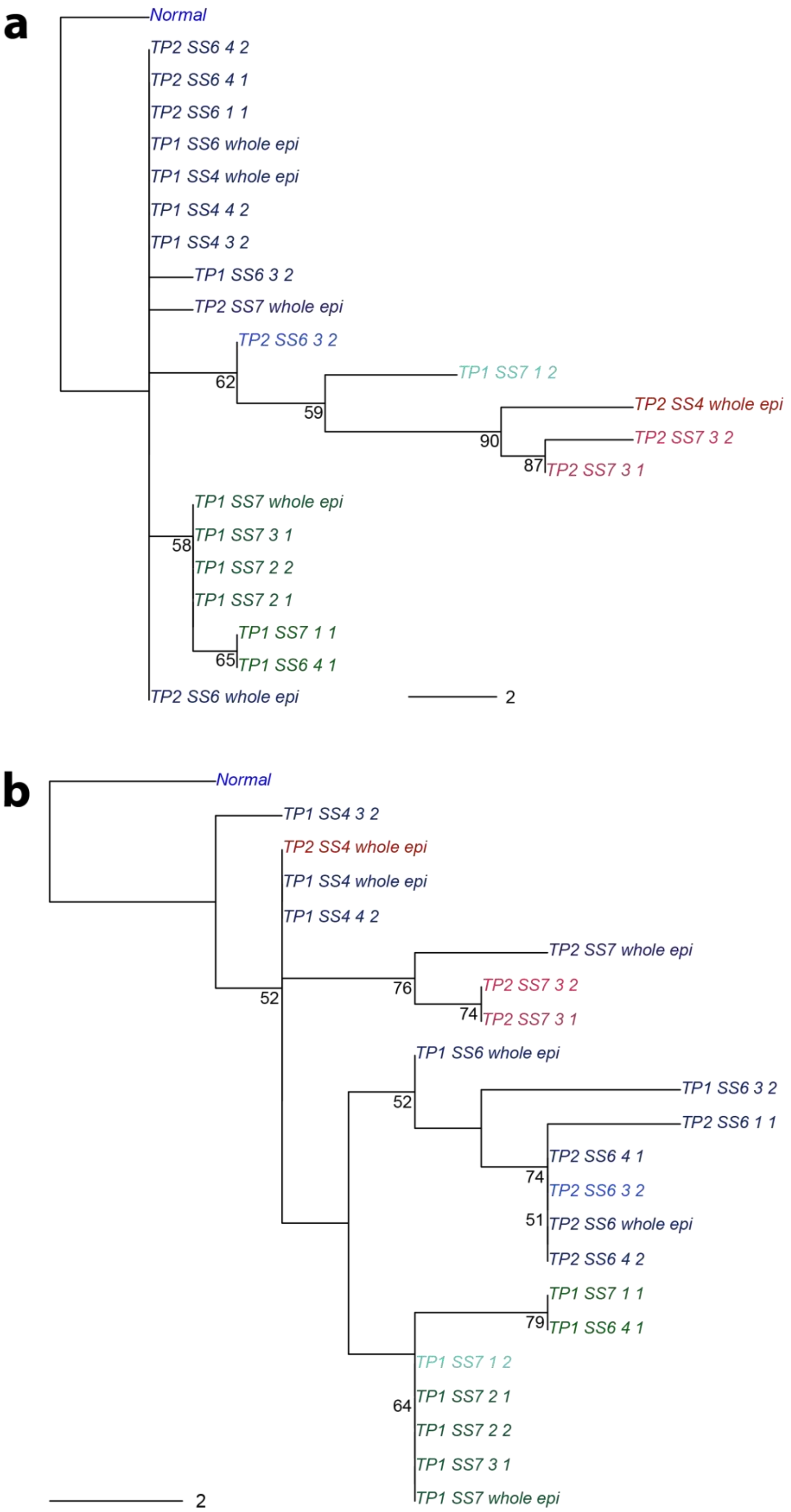

Supplementary Figure 38: Whole genome and fragile site trees for patient 451-NP. a) Whole genome tree. b) Fragile site tree. The fragile site tree was obtained similarly to the whole-genome tree based on fragile site breakpoints. Tip colours are the same as in the whole genome tree.

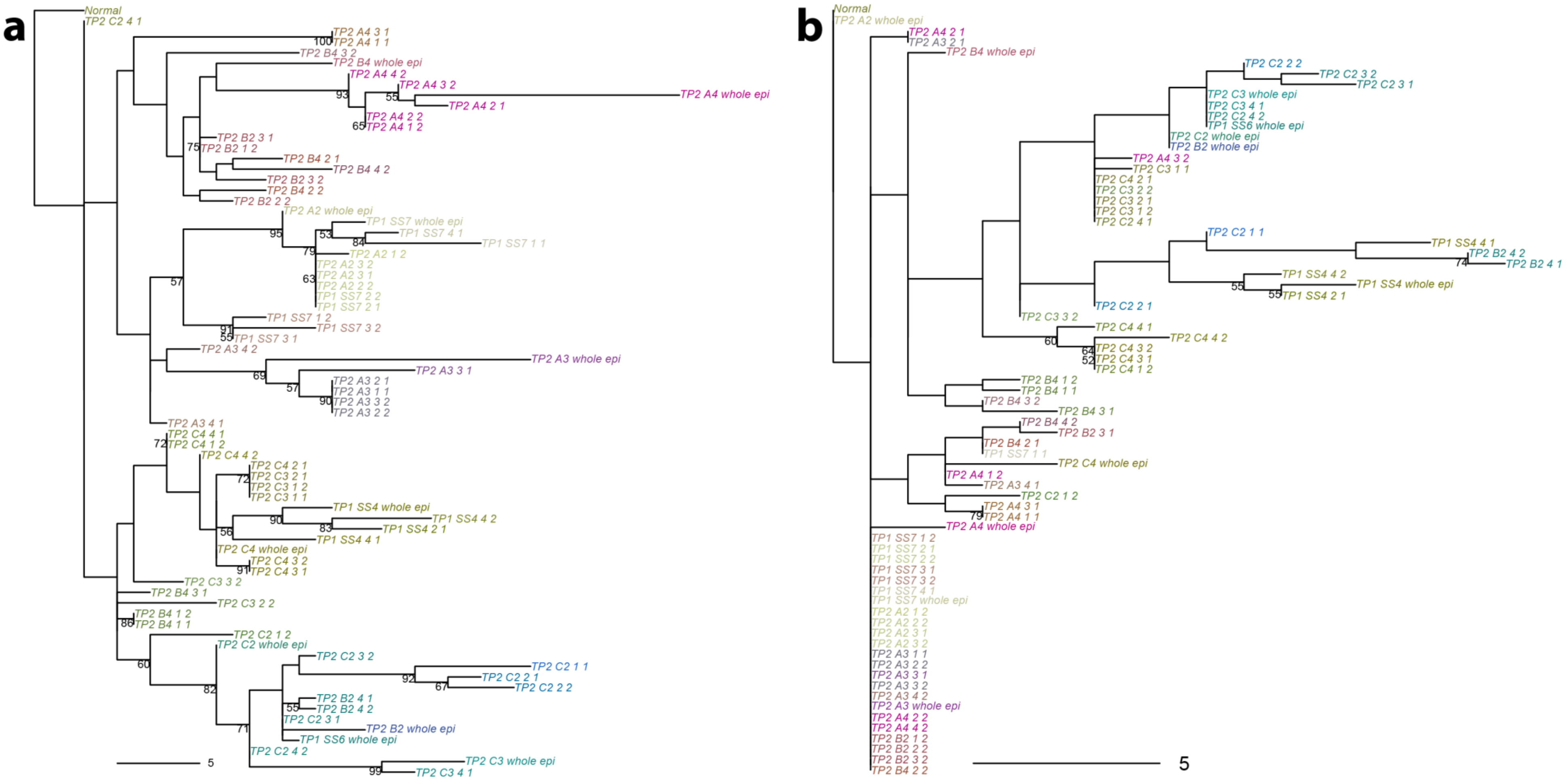

Supplementary Figure 39: Whole genome and fragile site trees for patient 740-P. a) Whole genome tree. b) Fragile site tree. The fragile site tree was obtained similarly to the whole-genome tree based on fragile site breakpoints. Tip colours are the same as in the whole genome tree.

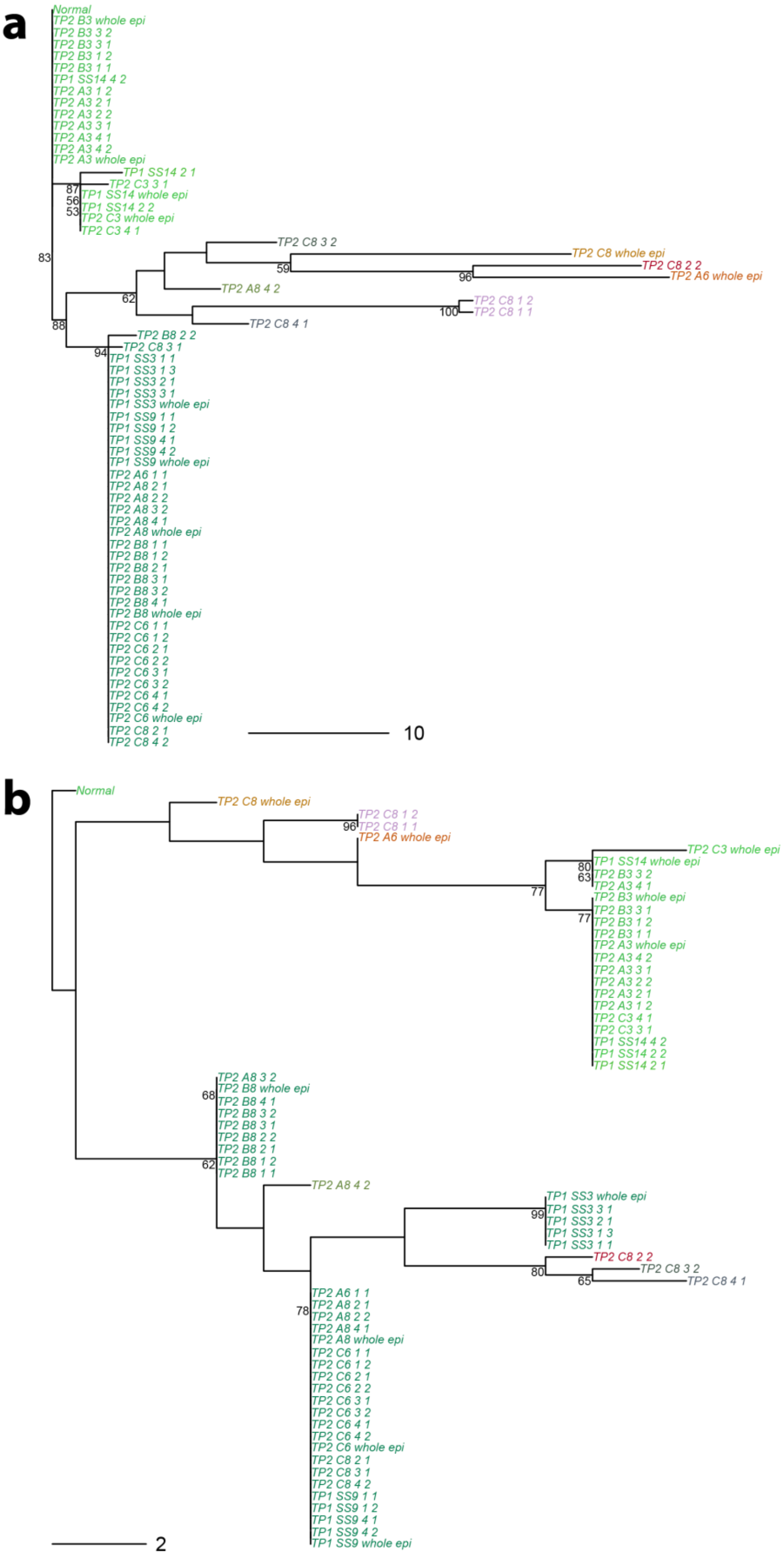

Supplementary Figure 40: Whole genome and fragile site trees for patient 848-P. a) Whole genome tree. b) Fragile site tree. The fragile site tree was obtained similarly to the whole-genome tree based on fragile site breakpoints. Tip colours are the same as in the whole genome tree.

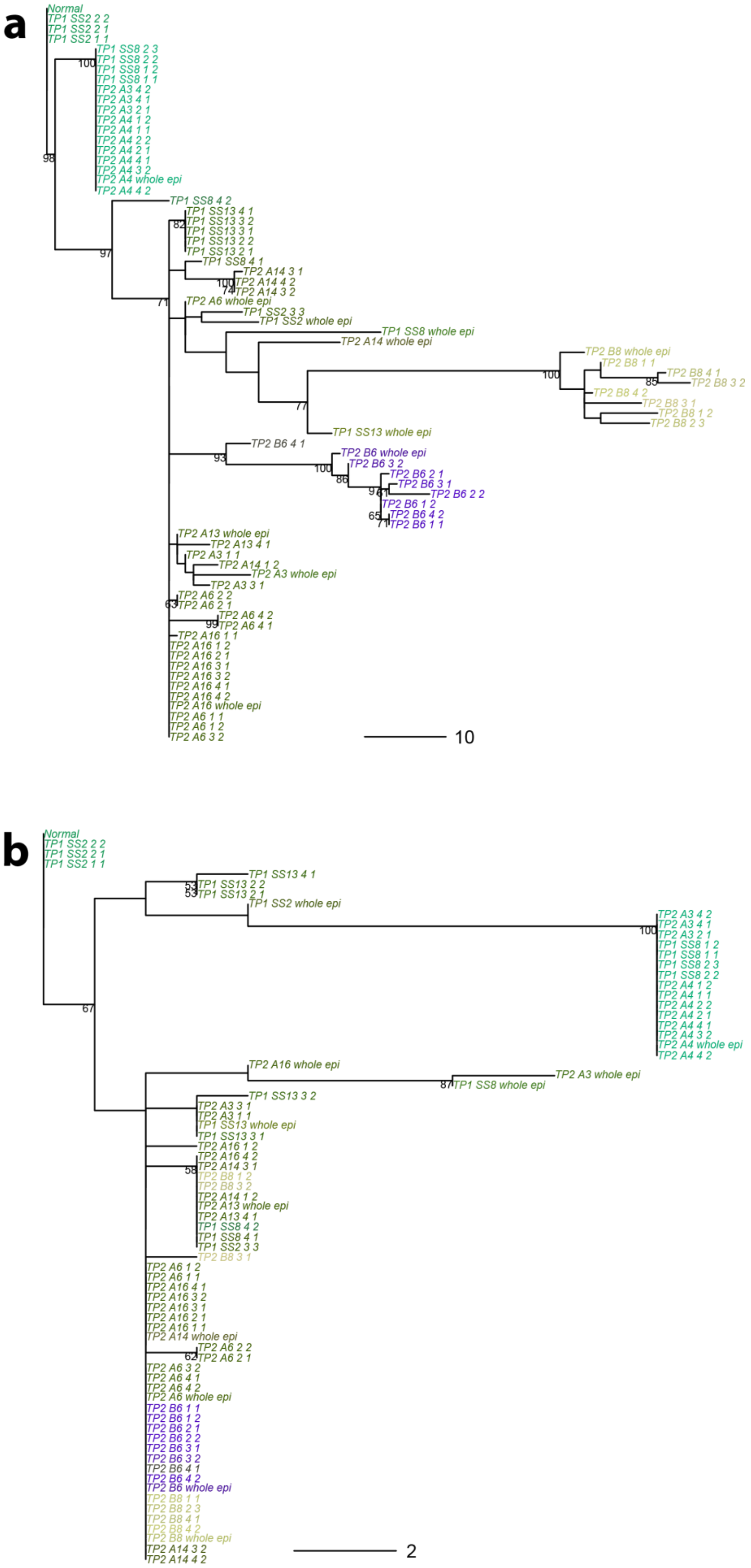

Supplementary Figure 41: Whole genome and fragile site trees for patient 852-P. a) Whole genome tree. b) Fragile site tree. The fragile site tree was obtained similarly to the whole-genome tree based on fragile site breakpoints. Tip colours are the same as in the whole genome tree.

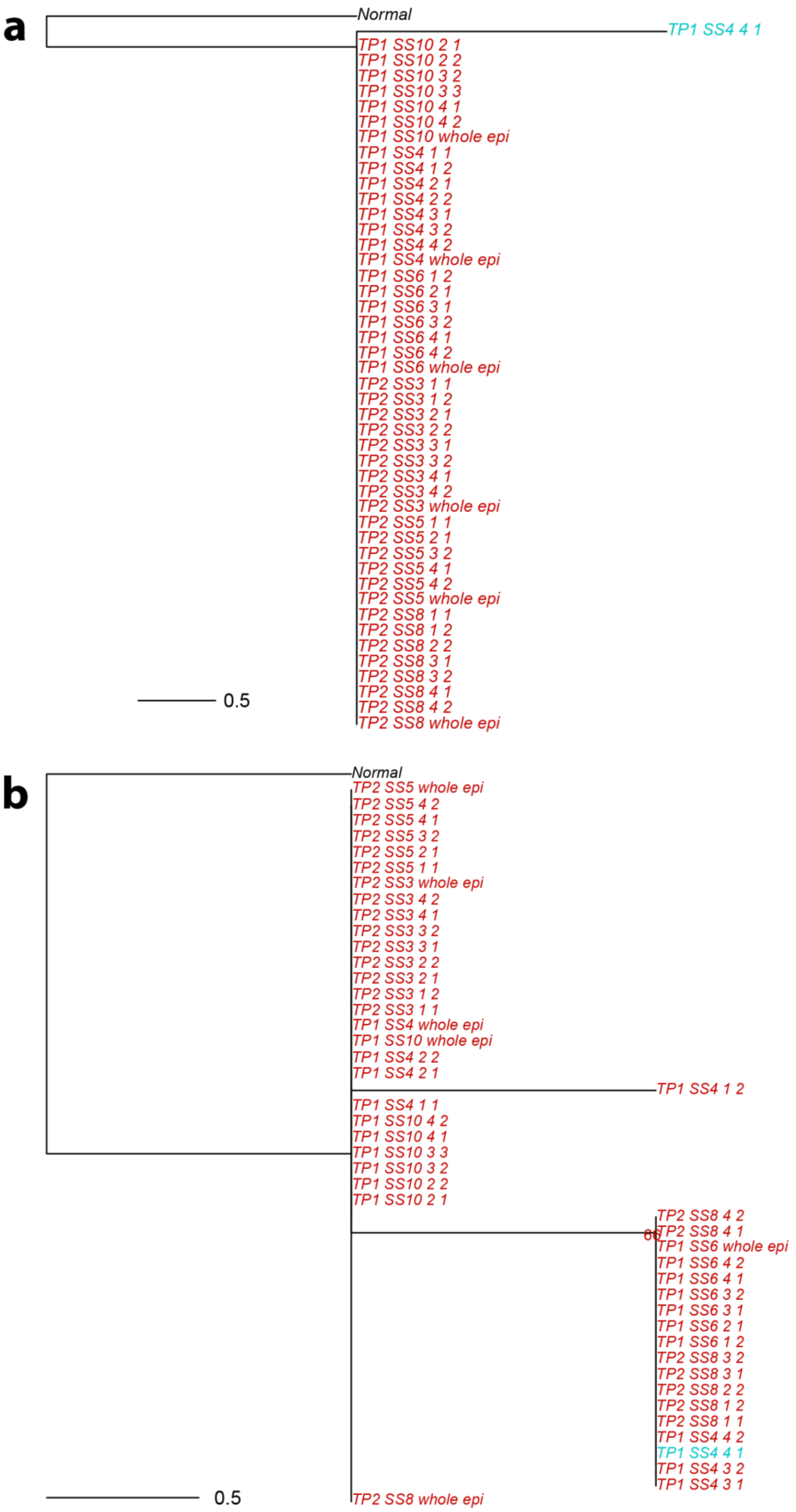

Supplementary Figure 42: Whole genome and fragile site trees for patient 911-NP. a) Whole genome tree. b) Fragile site tree. The fragile site tree was obtained similarly to the whole-genome tree based on fragile site breakpoints. Tip colours are the same as in the whole genome tree.

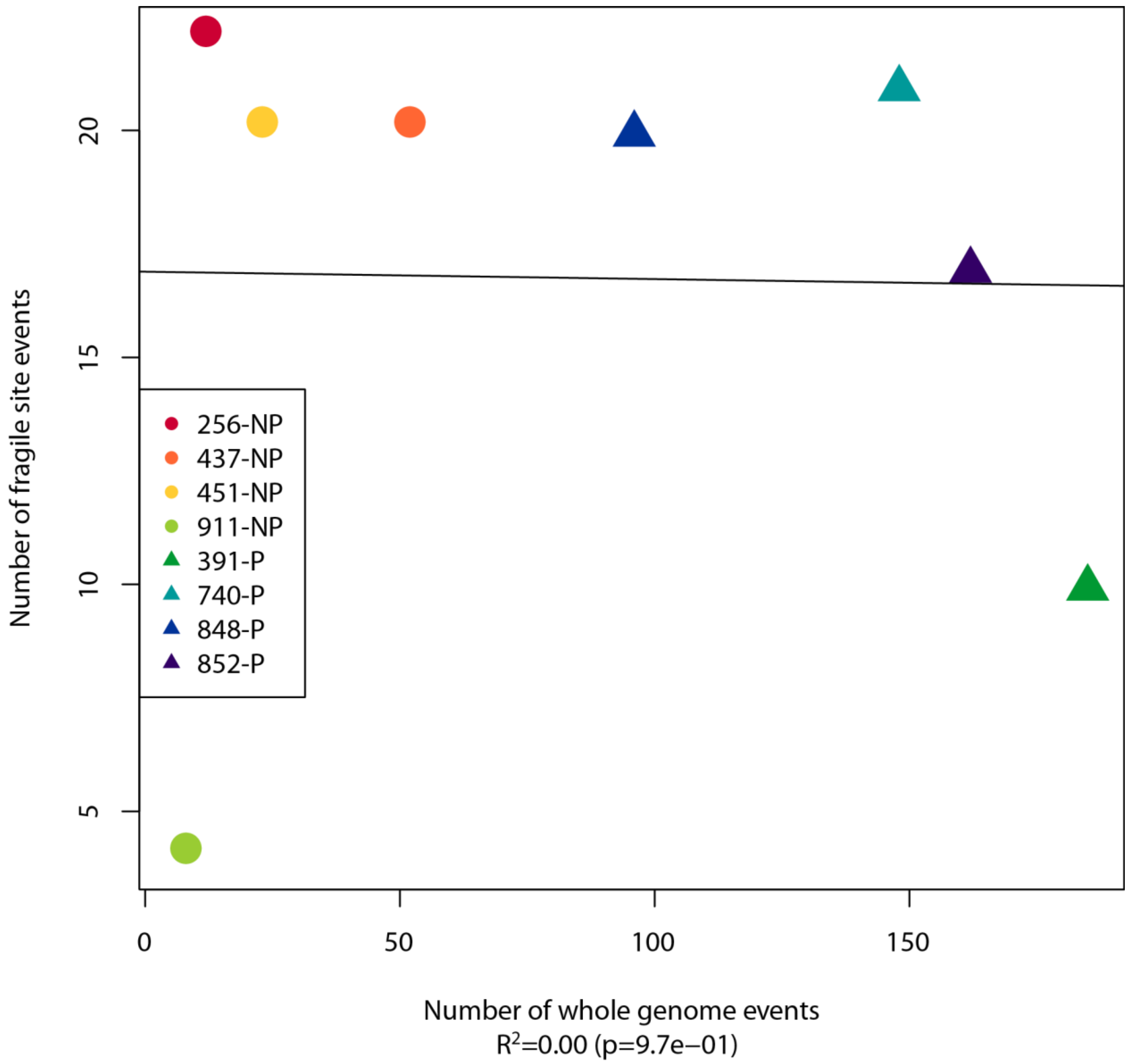

Supplementary Figure 43: Number of events in whole-genome and fragile site phylogenetic reconstructions in all 8 patients. Events used for phylogenetic reconstructions are breakpoints. No significant correlation was found. Circles highlight non-progressors and triangles highlight progressors. Each patient is identified by a colour. R^2 indicates the squared Pearson correlation coefficient used to test for significance.

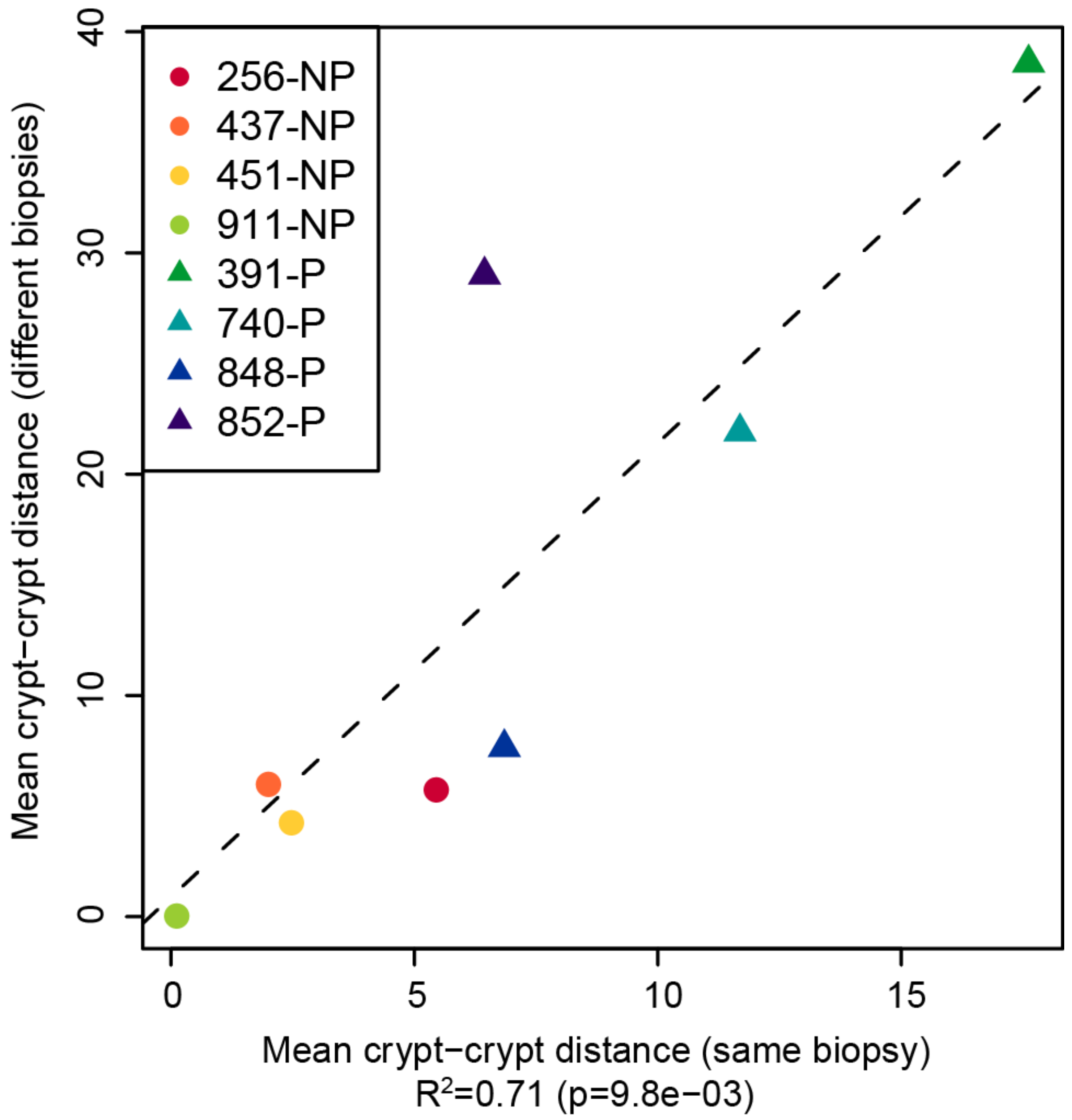

Supplementary Figure 44: Correlation between genetic distances at the micro and macro scales. Correlation of genetic distances between crypts from the same biopsy and crypts from different biopsies. All genetic distances in both groups (different crypts from the same biopsy; different crypts from different biopsies) are represented by their mean per patient. Progressors are displayed with triangles, non-progressors with circles and each patient is indicated by a different colour. R^2 indicates the squared Pearson correlation coefficient used to test for significance.

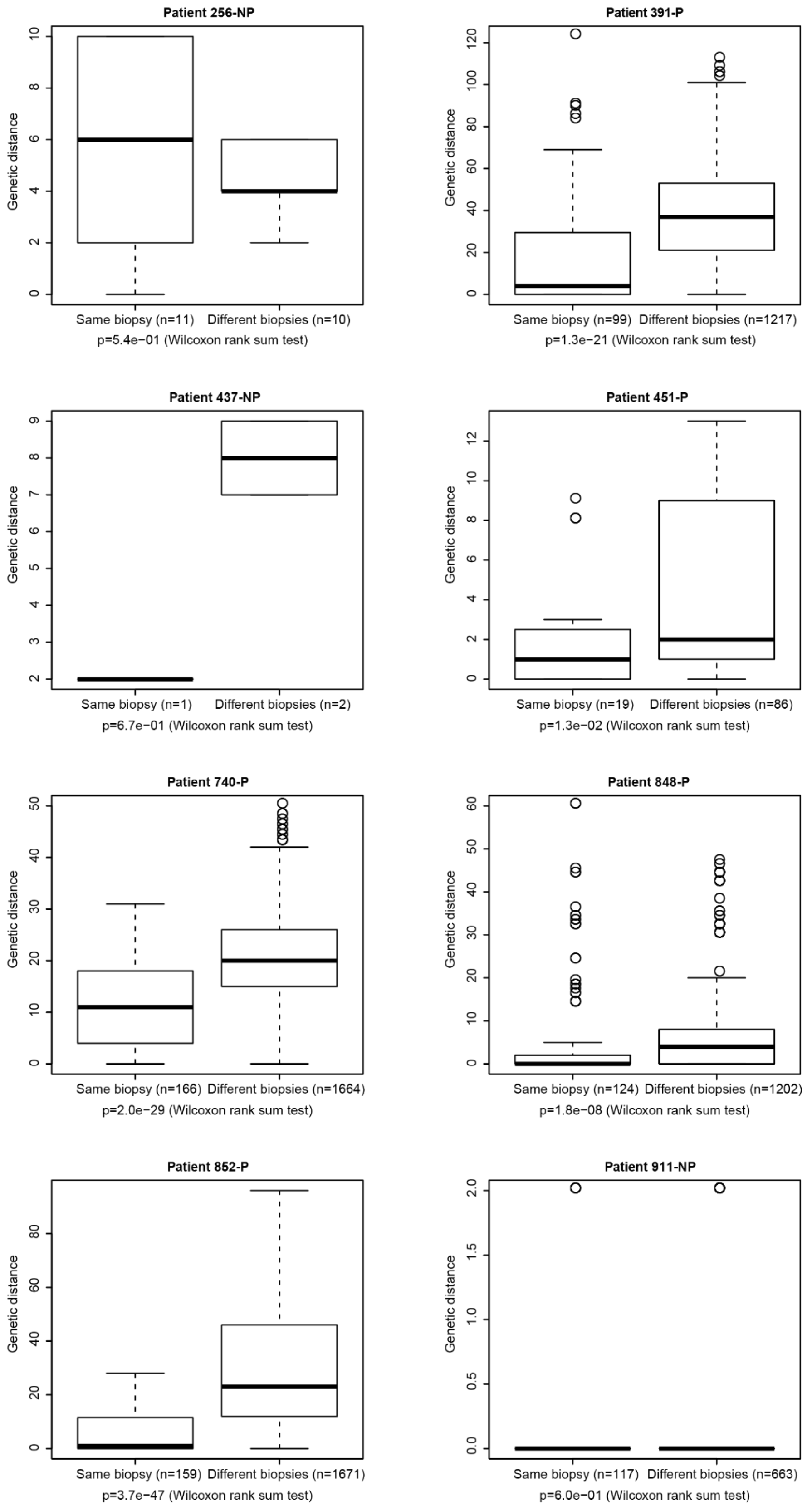

Supplementary Figure 45: Genetic distances between crypts from the same or different biopsies. Distributions of all pairwise crypt distances depending on whether the two crypts are from the same biopsy (left) or from different biopsies (right) in all 8 patients. Boxes indicate the middle quartiles, whiskers extend to 1.5 times the interquartile range, the horizontal bar indicates the median and dots represent outliers. All p values computed using Wilcoxon rank sum tests.

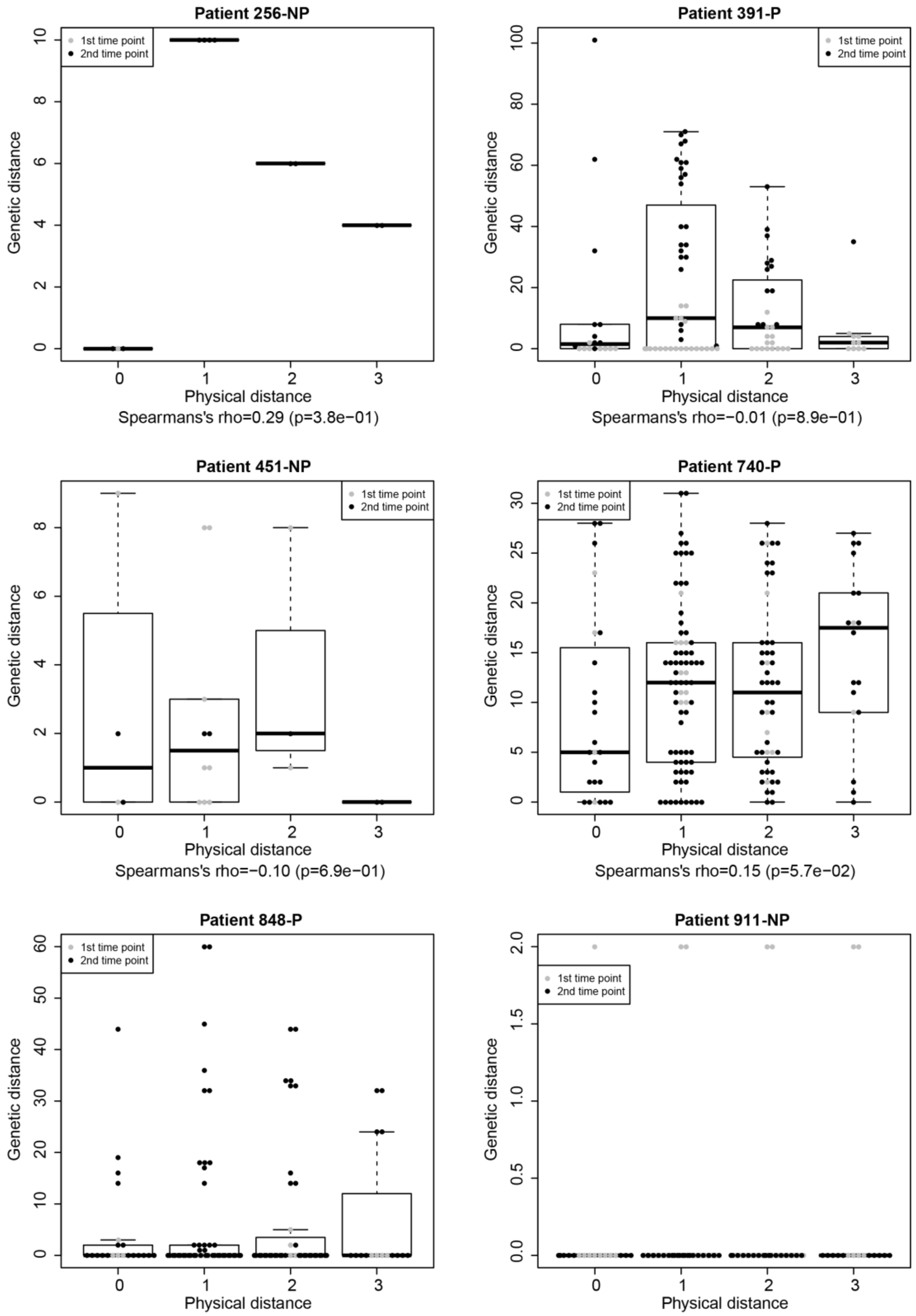

Supplementary Figure 46: Genetic distances across baguette sections with biopsies. Distributions of pairwise distances between crypts from the same biopsy according to the distance separating them in the baguette sections. The distance is 0 when crypts are in the same section, 1 if the sections are adjacent, 2 if there is one section in between them and 3 if there are 2 sections in between. Boxes indicate the middle quartiles, whiskers extend to 1.5 times the interquartile range and the horizontal bar indicates the median. Dots represent each individual observation, first time point observations are in grey, second time point ones are in black. Correlations were tested for significance using Spearman’s non-parametric rho coefficient.

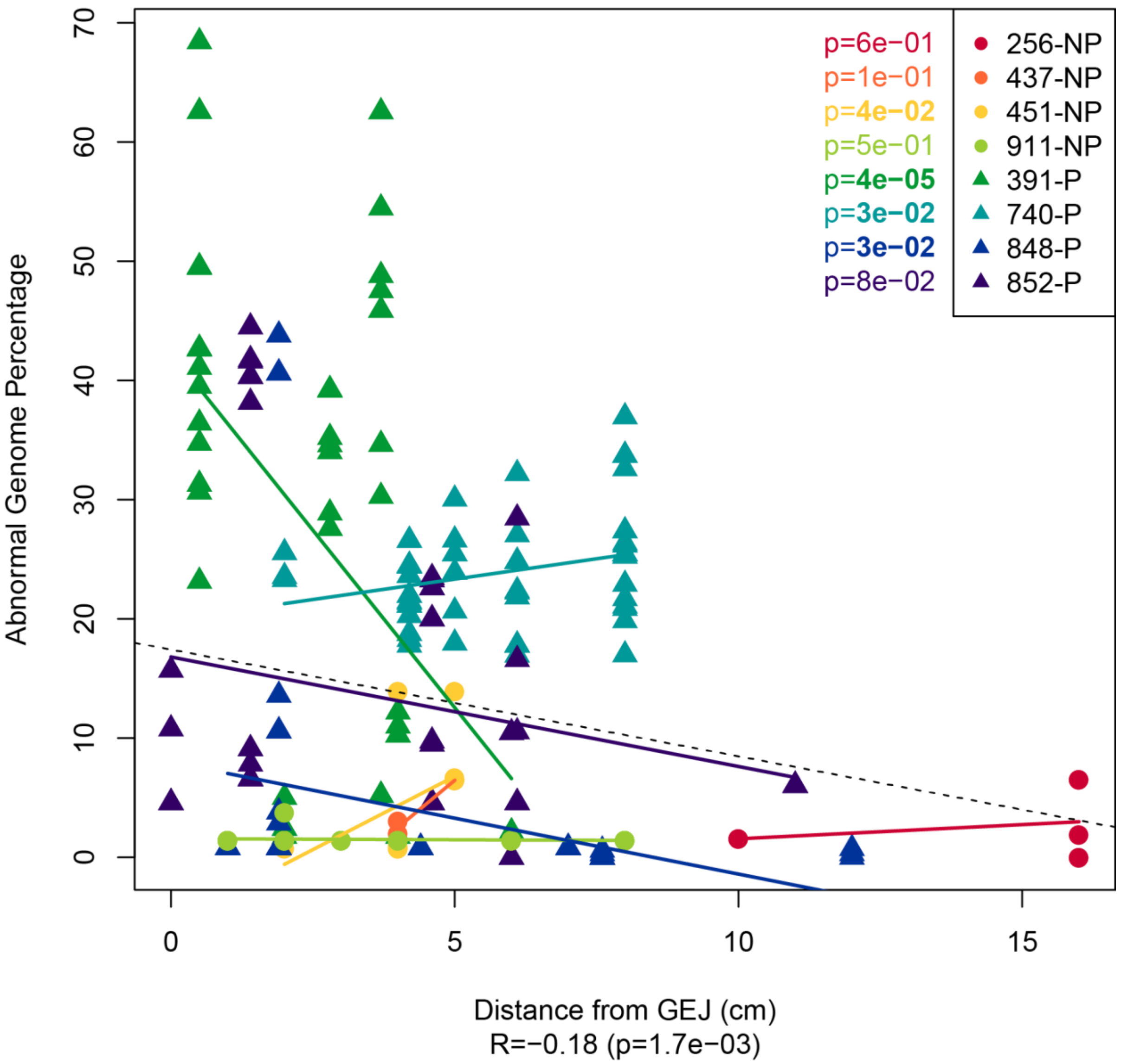

Supplementary Figure 47: Abnormal genome percentage per crypt according to the distance from the Gastro-Esophageal Junction (GEJ). Each crypt and its abnormal genome percentage is represented by a point, the colour of which indicates the patient it originates from (progressors as triangles, non-progressors as circles). Coloured lines indicate the linear fit for each patient, while the dotted black line indicates the overall fit. Individual p values for Pearson correlation coefficients per patient are indicated in the top right corner, bold font highlights statistical significance. The overall Pearson correlation coefficient R is indicated below the plot along with its p value.

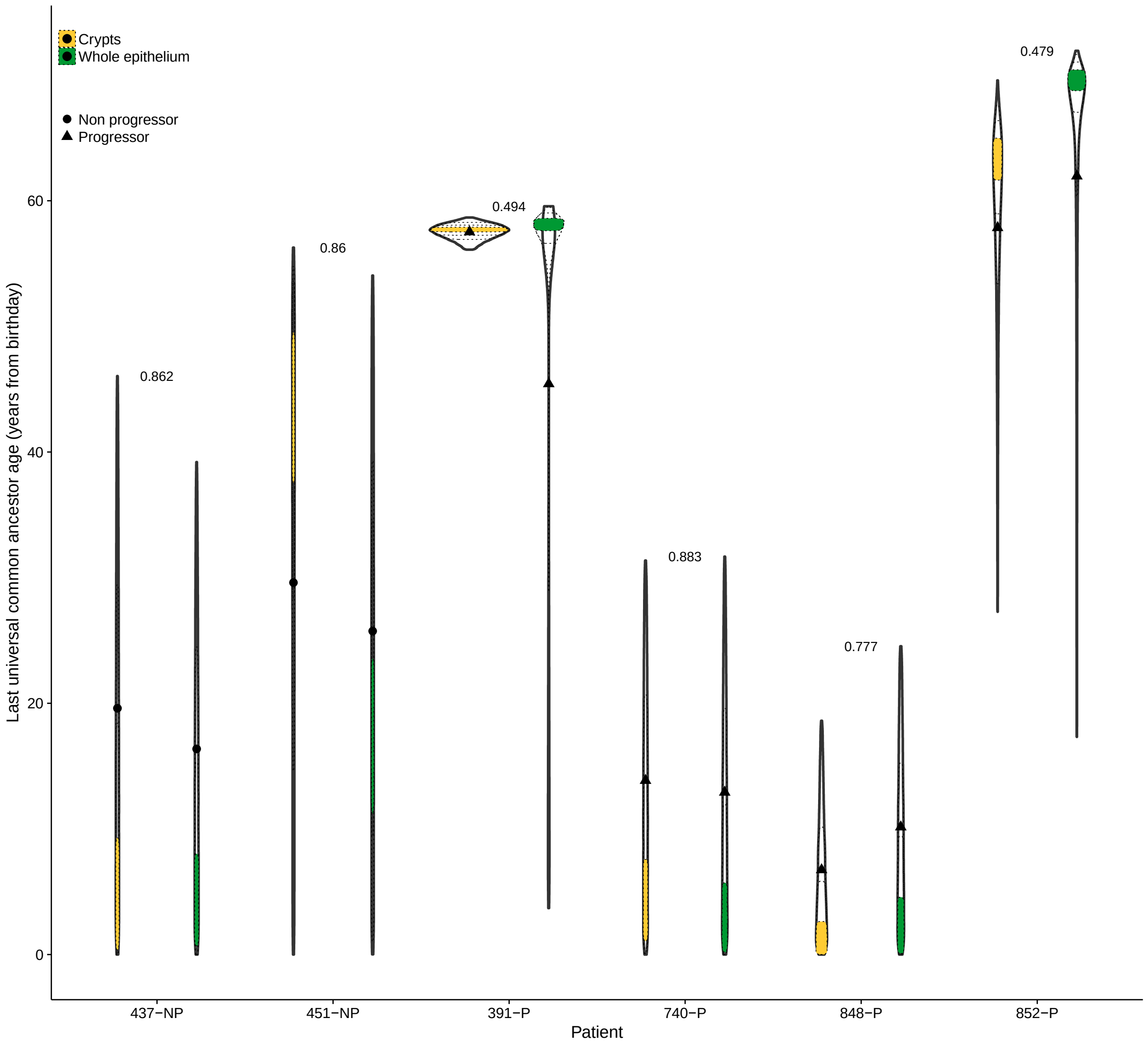

Supplementary Figure 48: Last universal common ancestor age estimation. Each violin plot corresponds to the posterior sample of the LUCA age for a given patient (x axis) and dataset (color, yellow=crypt, green=whole epithelium) included in the 95% highest posterior density (HPD) interval. The mean rate is indicated with a dot for non progressors and a triangle for progressors. Inscribed posterior samples with decreasing transparency correspond to the 75%, 50% and 25% of the HPD interval. The probability of the two posteriors distribution being the same is indicated per patient.

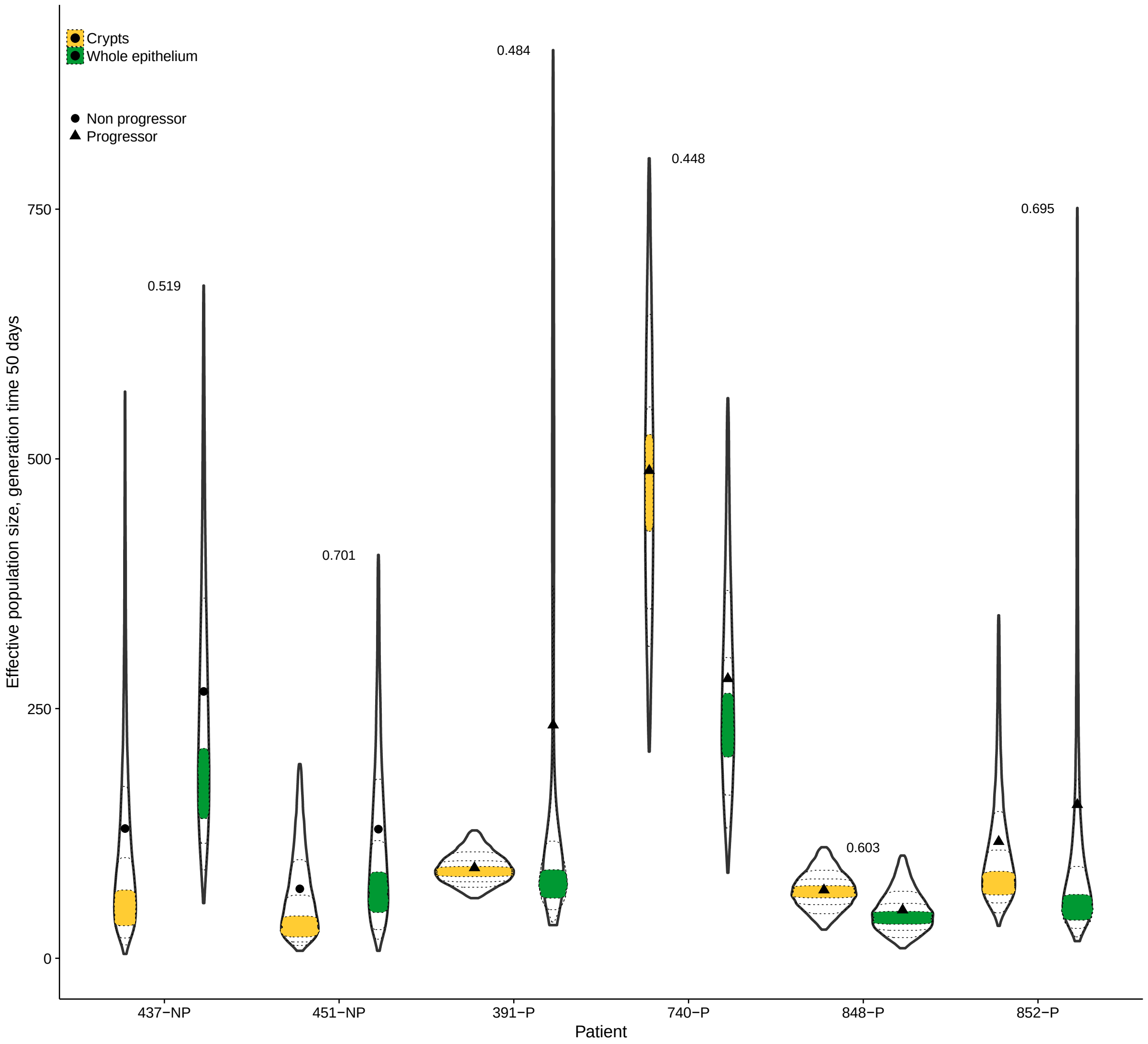

Supplementary Figure 49: Effective population size estimation assuming a 50-day generation time. Each violin plot corresponds to the posterior sample of the effective population size estimation for a given patient (x axis) and dataset (color, yellow=crypt, green=whole epithelium) included in the 95% highest posterior density (HPD) interval. The mean rate is indicated with a dot for non progressors and a triangle for progressors. Inscribed posterior samples with decreasing transparency correspond to the 75%, 50% and 25% of the HPD interval. The probability of the two posteriors distribution being the same is indicated per patient.

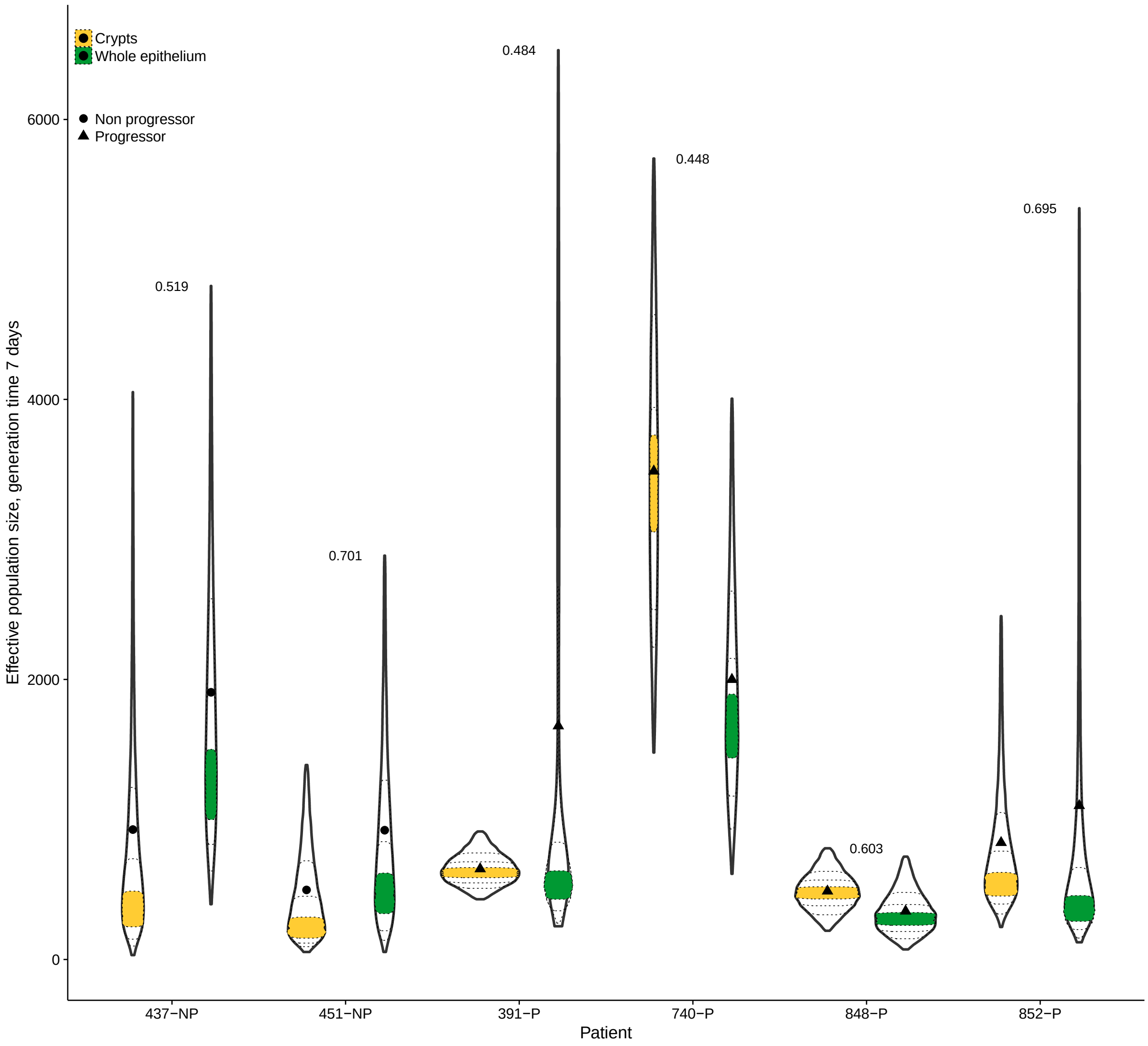

Supplementary Figure 50: Effective population size estimation assuming a 7-day generation time. Each violin plot corresponds to the posterior sample of the effective population size estimation for a given patient (x axis) and dataset (color, yellow=crypt, green=whole epithelium) included in the 95% highest posterior density (HPD) interval. The mean rate is indicated with a dot for non progressors and a triangle for progressors. Inscribed posterior samples with decreasing transparency correspond to the 75%, 50% and 25% of the HPD interval. The probability of the two posteriors distribution being the same is indicated per patient.

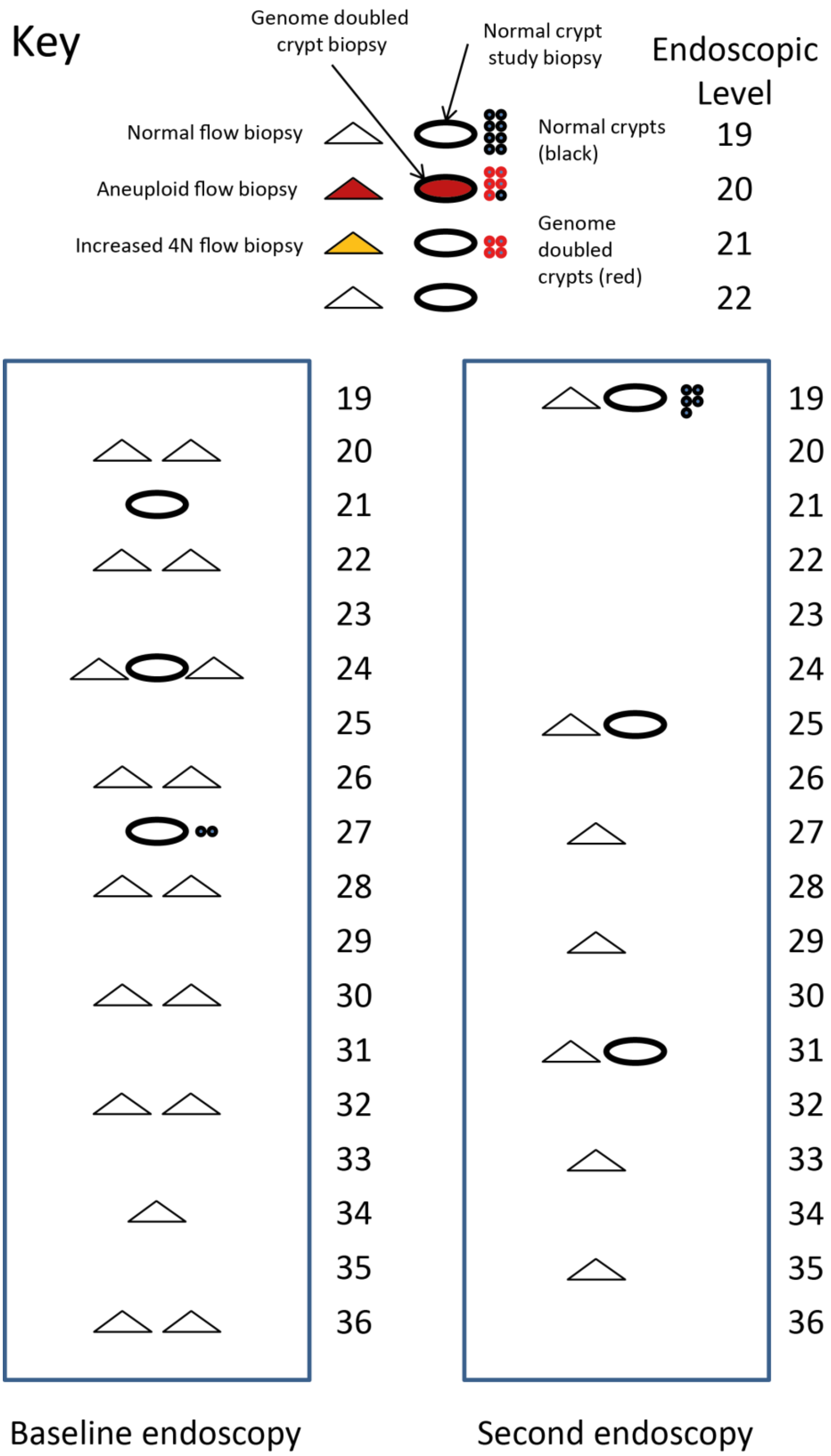

Supplementary Figure 51: Genome doubling in patient 256. Endoscopic and surgical maps indicating the spatial orientation of biopsies relative to each other in the from the endoscopic sampling (endoscopic level indicates distance from incisors as measured by endoscope) and from the surgical specimens. Increased 4N biopsies are those with a 4N DNA content of greater than 6%; aneuploid biopsies have a G1 peak of 2.2N or greater. Genome double crypts and biopsies are as defined in the manuscript.

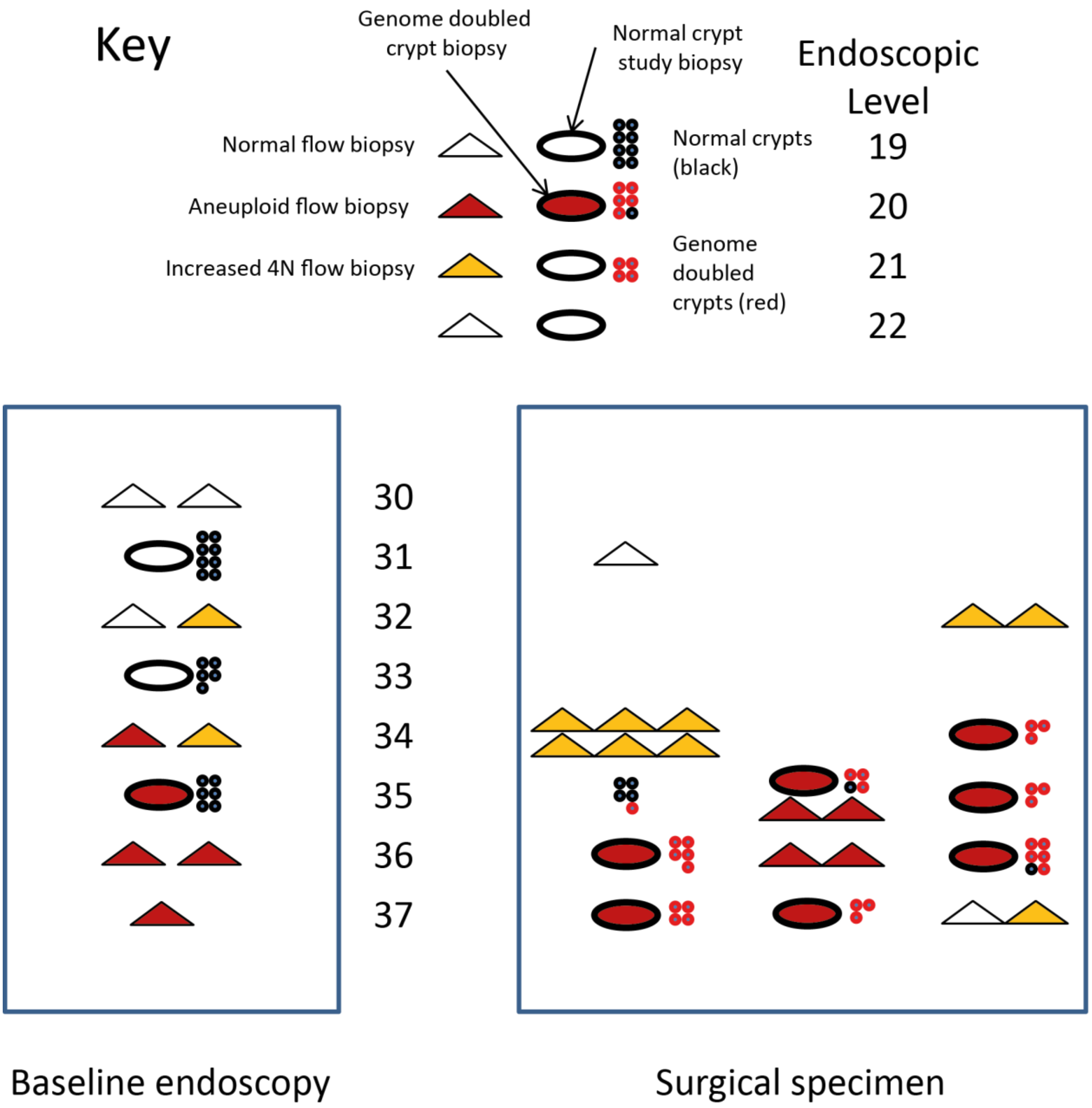

Supplementary Figure 52: Genome doubling in patient 391. Endoscopic and surgical maps indicating the spatial orientation of biopsies relative to each other in the from the endoscopic sampling (endoscopic level indicates distance from incisors as measured by endoscope) and from the surgical specimens. Increased 4N biopsies are those with a 4N DNA content of greater than 6%; aneuploid biopsies have a G1 peak of 2.2N or greater. Genome double crypts and biopsies are as defined in the manuscript.

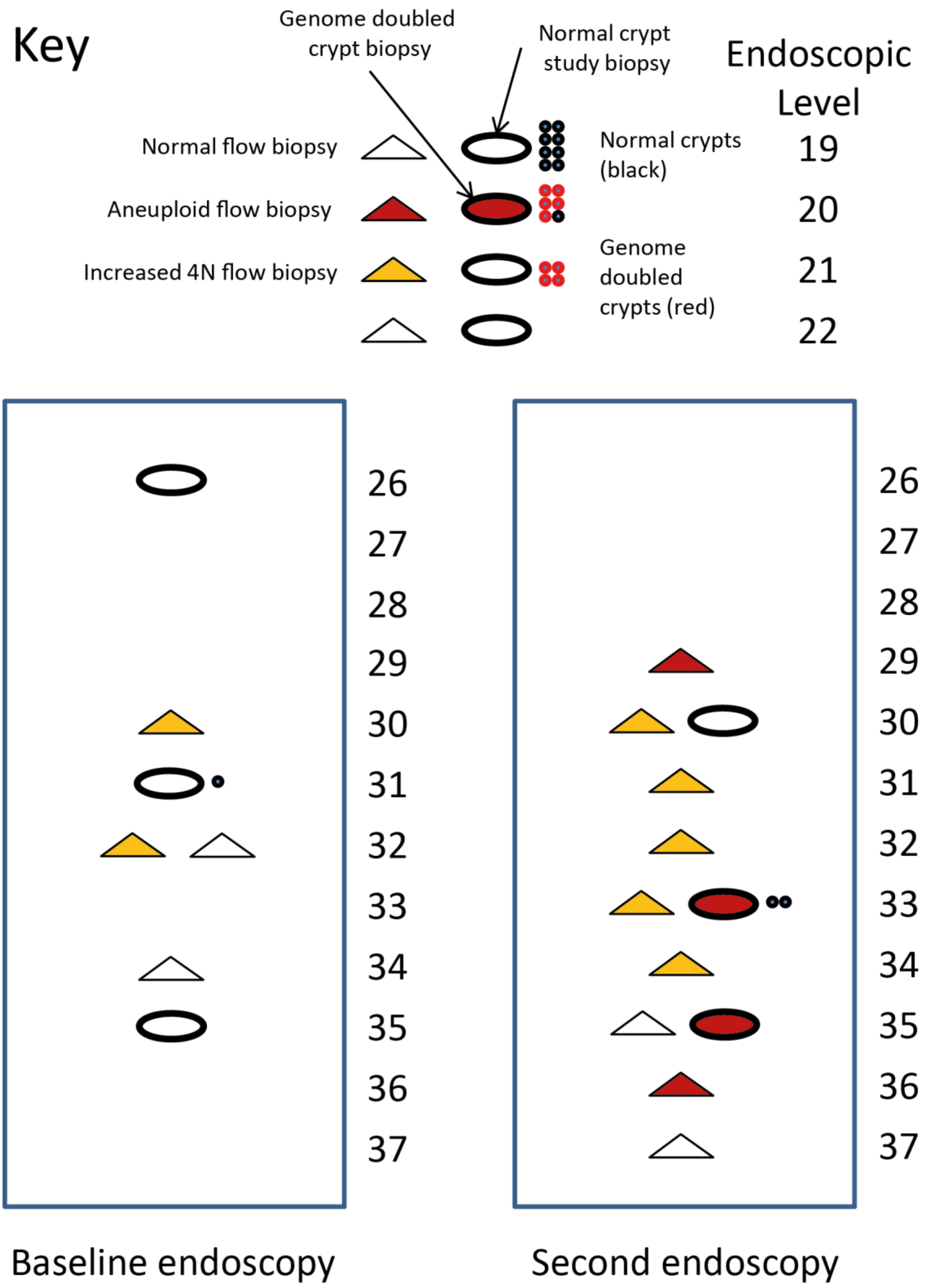

Supplementary Figure 53: Genome doubling in patient 437. Endoscopic and surgical maps indicating the spatial orientation of biopsies relative to each other in the from the endoscopic sampling (endoscopic level indicates distance from incisors as measured by endoscope) and from the surgical specimens. Increased 4N biopsies are those with a 4N DNA content of greater than 6%; aneuploid biopsies have a G1 peak of 2.2N or greater. Genome double crypts and biopsies are as defined in the manuscript.

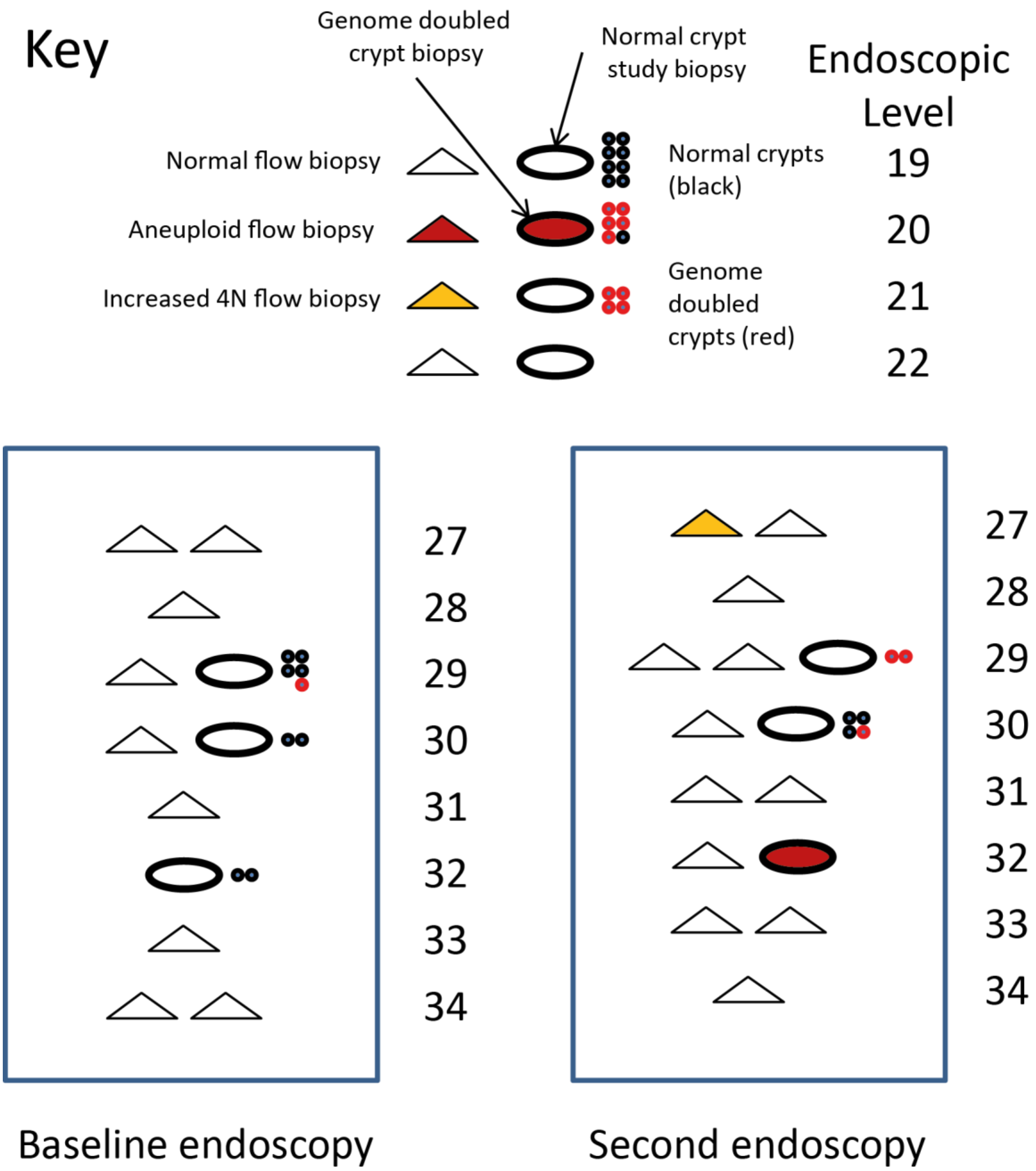

Supplementary Figure 54: Genome doubling in patient 451. Endoscopic and surgical maps indicating the spatial orientation of biopsies relative to each other in the from the endoscopic sampling (endoscopic level indicates distance from incisors as measured by endoscope) and from the surgical specimens. Increased 4N biopsies are those with a 4N DNA content of greater than 6%; aneuploid biopsies have a G1 peak of 2.2N or greater. Genome double crypts and biopsies are as defined in the manuscript.

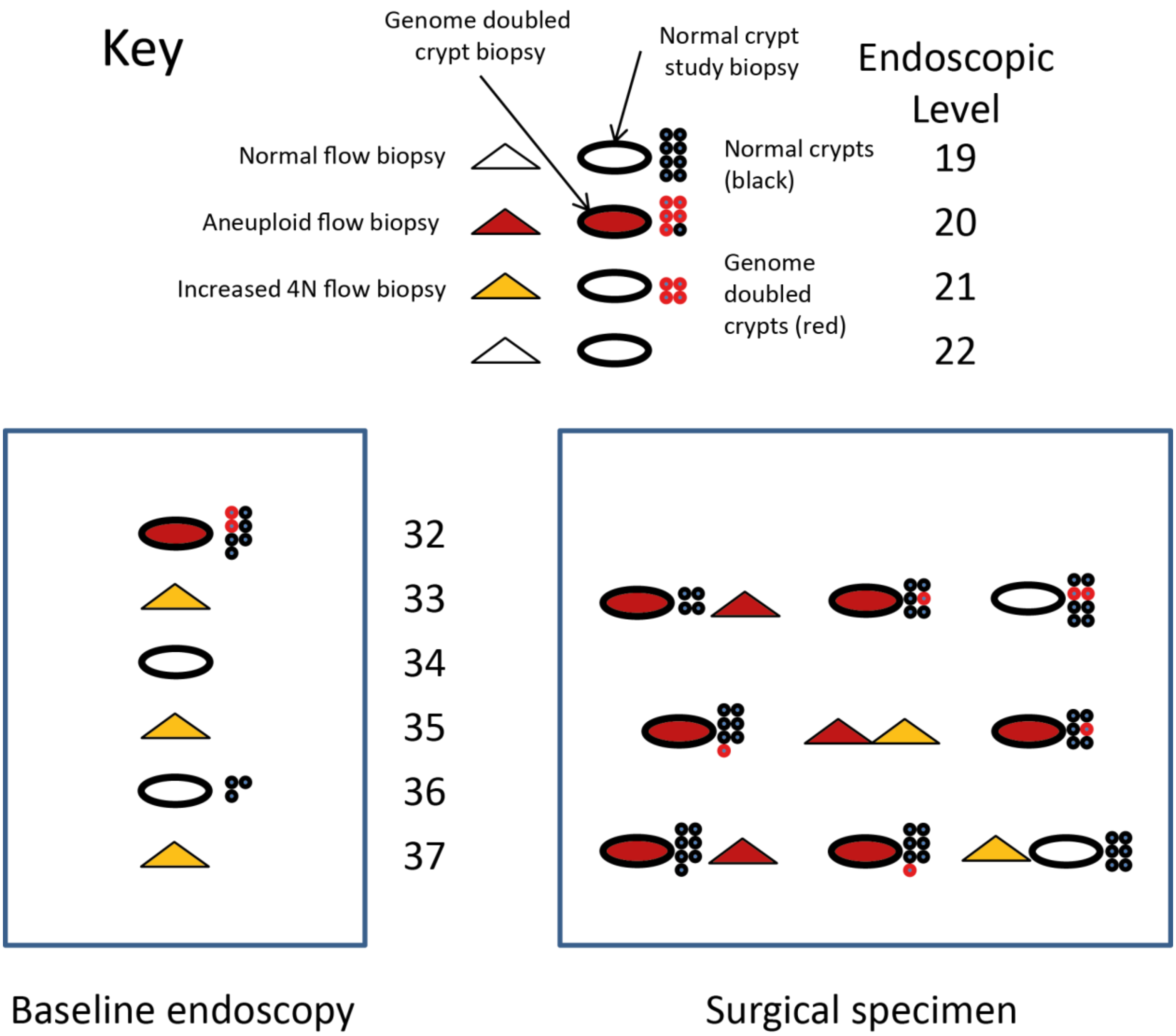

Supplementary Figure 55: Genome doubling in patient 740. Endoscopic and surgical maps indicating the spatial orientation of biopsies relative to each other in the from the endoscopic sampling (endoscopic level indicates distance from incisors as measured by endoscope) and from the surgical specimens. Increased 4N biopsies are those with a 4N DNA content of greater than 6%; aneuploid biopsies have a G1 peak of 2.2N or greater. Genome double crypts and biopsies are as defined in the manuscript.

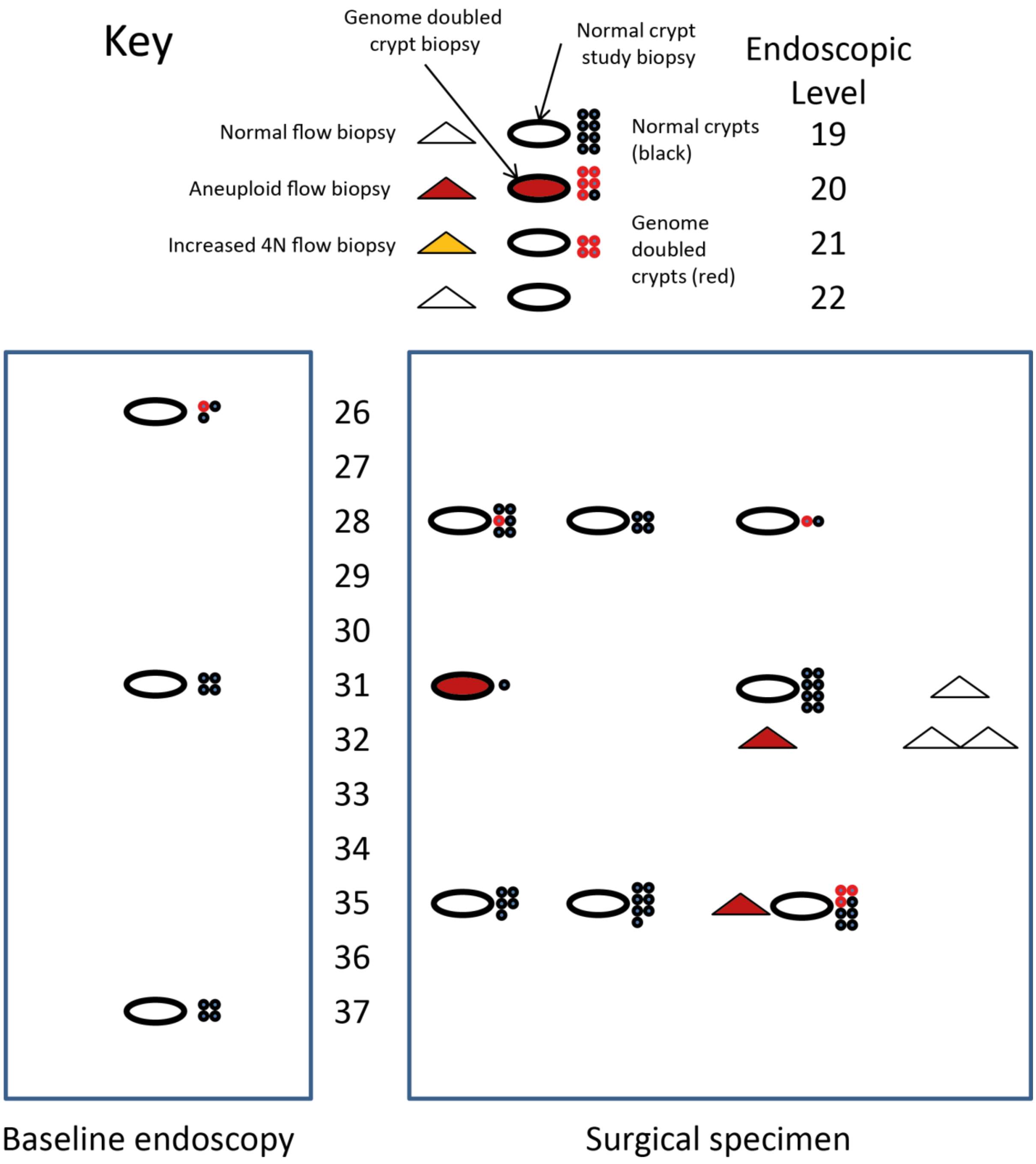

Supplementary Figure 56: Genome doubling in patient 848. Endoscopic and surgical maps indicating the spatial orientation of biopsies relative to each other in the from the endoscopic sampling (endoscopic level indicates distance from incisors as measured by endoscope) and from the surgical specimens. Increased 4N biopsies are those with a 4N DNA content of greater than 6%; aneuploid biopsies have a G1 peak of 2.2N or greater. Genome double crypts and biopsies are as defined in the manuscript.

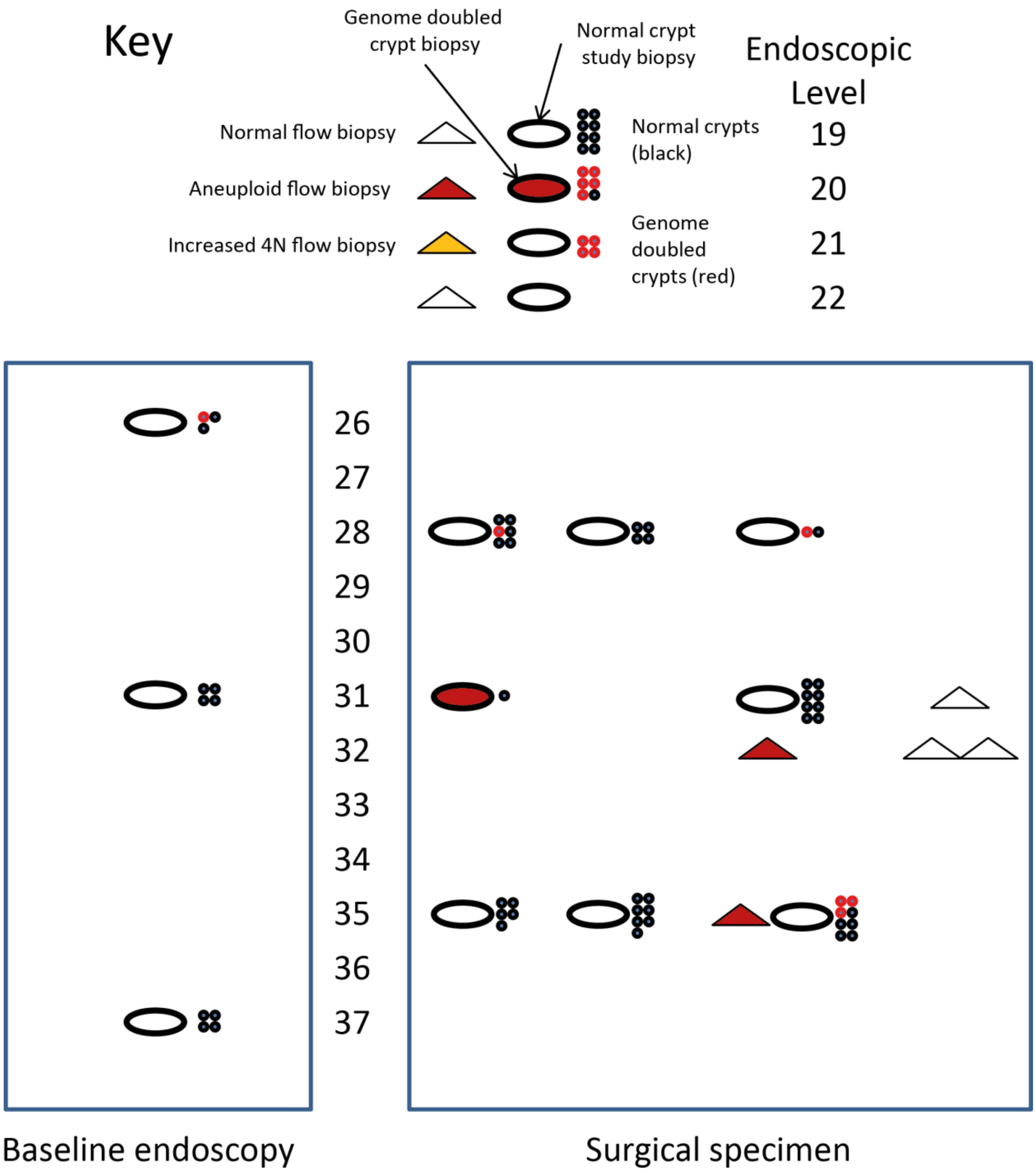

Supplementary Figure 57: Genome doubling in patient 852. Endoscopic and surgical maps indicating the spatial orientation of biopsies relative to each other in the from the endoscopic sampling (endoscopic level indicates distance from incisors as measured by endoscope) and from the surgical specimens. Increased 4N biopsies are those with a 4N DNA content of greater than 6%; aneuploid biopsies have a G1 peak of 2.2N or greater. Genome double crypts and biopsies are as defined in the manuscript.

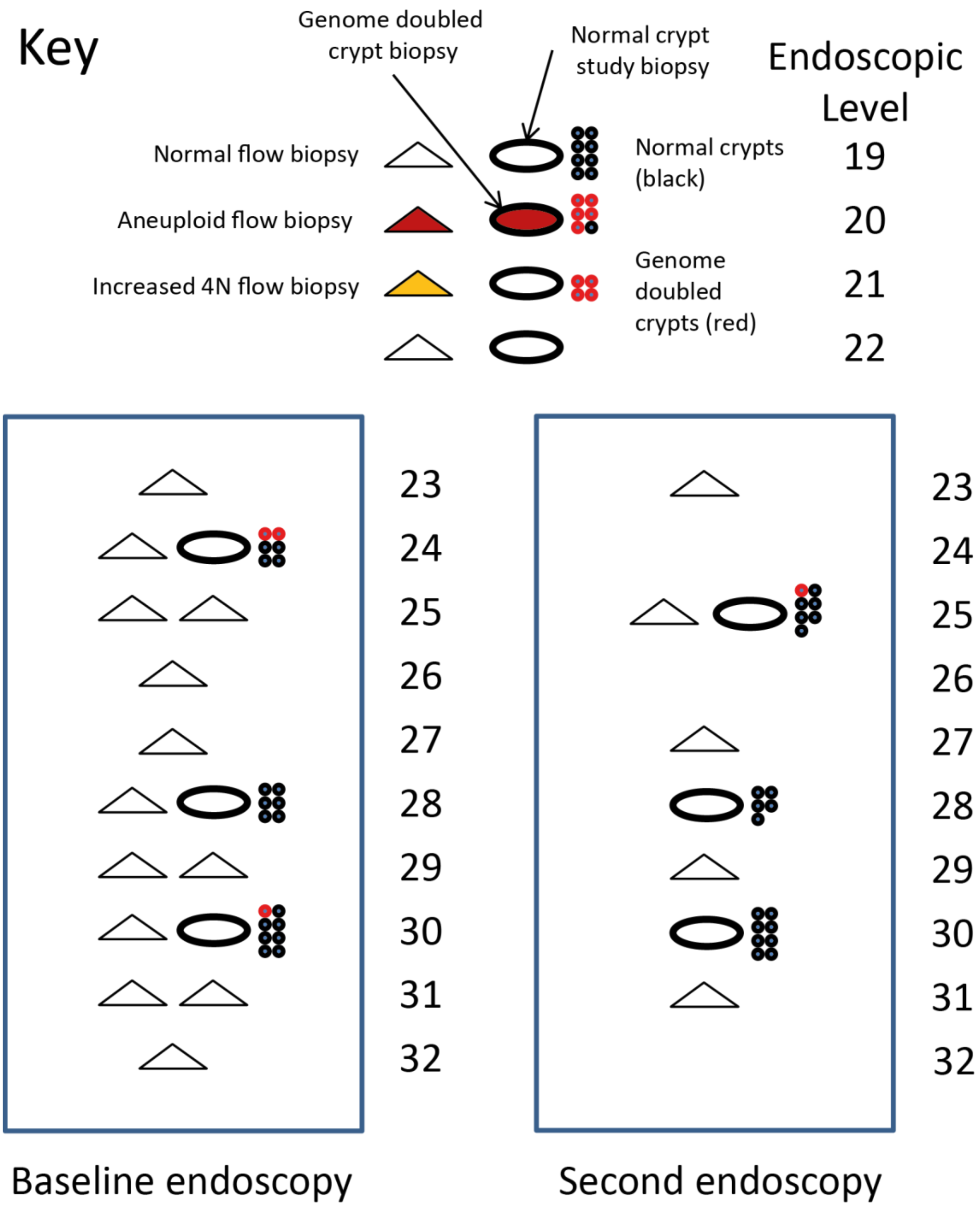

Supplementary Figure 58: Genome doubling in patient 911. Endoscopic and surgical maps indicating the spatial orientation of biopsies relative to each other in the from the endoscopic sampling (endoscopic level indicates distance from incisors as measured by endoscope) and from the surgical specimens. Increased 4N biopsies are those with a 4N DNA content of greater than 6%; aneuploid biopsies have a G1 peak of 2.2N or greater. Genome double crypts and biopsies are as defined in the manuscript.

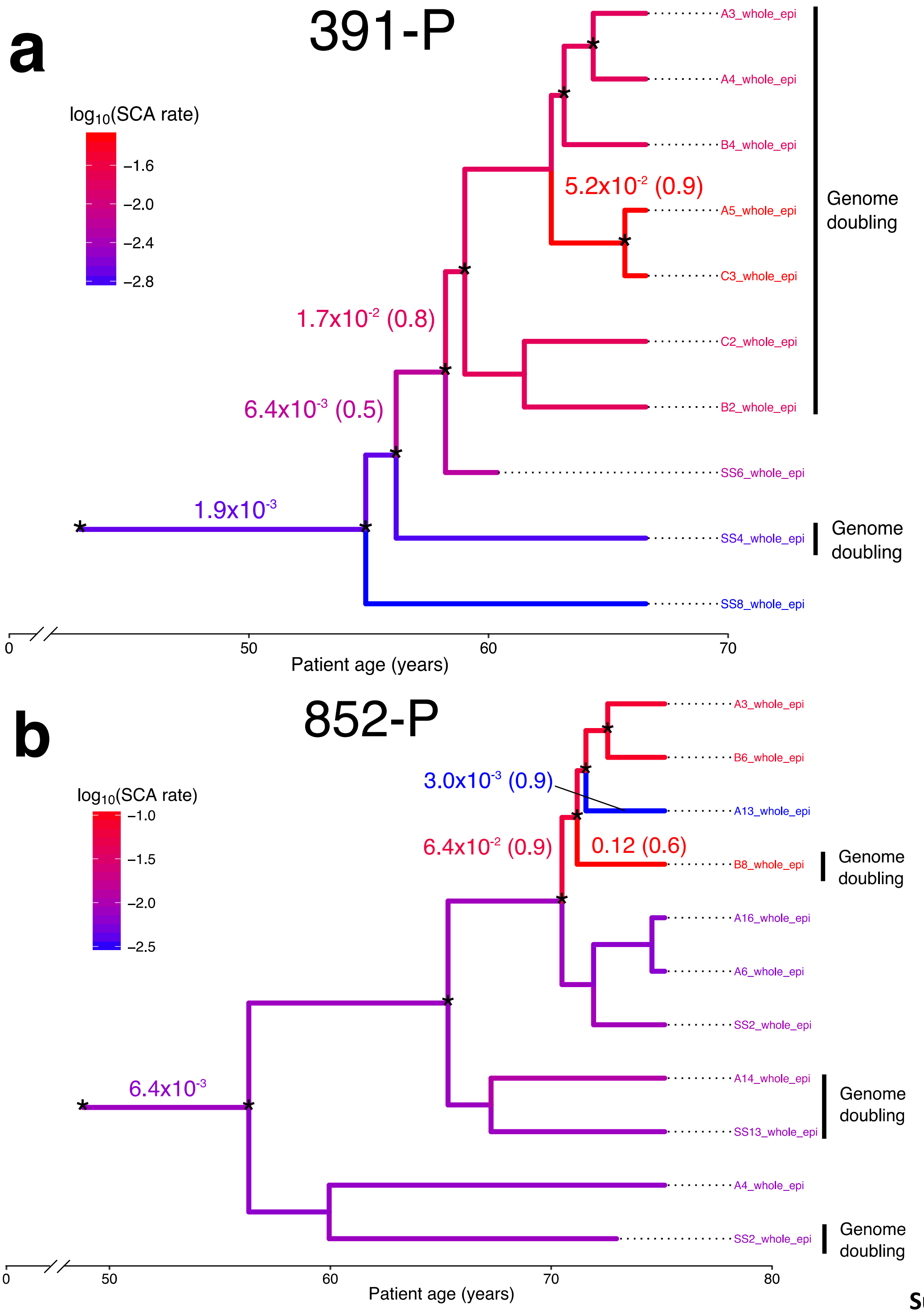

Supplementary Figure 59: Estimated SCA mutation rate changes in patients 852-P and 391-P using biopsy data. Phylogenetic trees of patients 391-P (a) and 852-P (b). Branch lengths indicate time measured in years, while branch colors indicate the log10 of the estimated SCA rate. SCA rate changes are indicated numerically with their estimated mean rate, accompanied by their posterior probability conditional to the clade defined by their branch (between parentheses). The original rate is indicated without posterior probability, since it does not constitute a rate change. Nodes with a posterior probability >0.80 are labelled with an asterisk. The x-axis indicates years from the birth of the patient. Samples with estimated genome doubling are also labelled.

SUPPLEMENTARY METHODS

Figures and tables specific to this section are embedded in the text.

Pre-processing and quality control

Standard quality control was performed using the Illumina GenomeStudio software. 216 samples did not pass the quality control (0/1 column in default GenomeStudio output) and were excluded from further analysis. logR values were corrected for GC content bias using the genomic wave correction tool of the pennCNV software suite[1].

Probe filtering

Because of the low initial DNA quantity extracted from single crypts, biases were introduced in the SNP array data for these samples in the form of downward 'spikes' in the logR data, similar to what would occur in double deletions, which were found to be recurrent across samples and patients (**Supplementary Figure 60**).

We therefore proceeded to filter these out to avoid it impacting both segmentation and copy number profiling. From the initial set of probes S0, a subset S25 of probes is selected by taking a probe every 25 probes, and compute *M* the median logR and *d* the median average deviation from 100 probes before and after. The median of these medians is calculated similarly, taking a probe *s* out of every 5 of probes from the S25 set (so 1 in every 5 * 25 = 125 probes overall). *Mm*, the median of medians is computed using 50 probes from the S25 set before and after the selected probe. For the region relative to *s*, all the probes from S0 that are included in the calculation (5 * 25 * 50 +100 probes each side). Any probe that deviates from *Mm* by more than 5 times m (initial median average deviation) is excluded (**Supplementary Figure 61**).

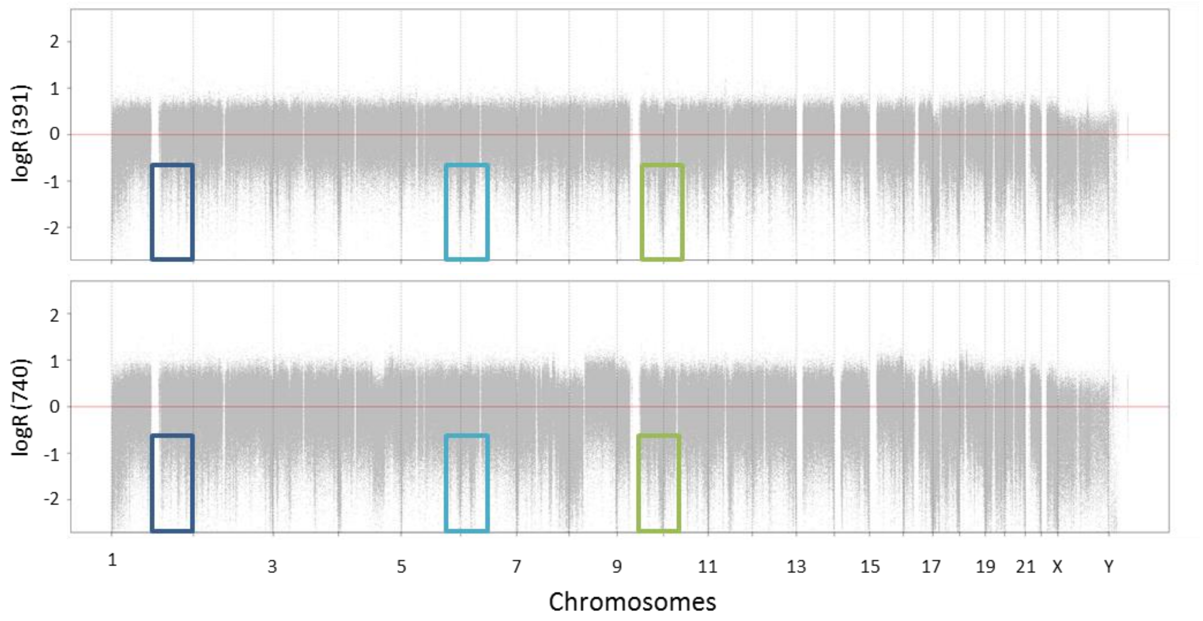

Supplementary Figure 60: logR artifacts across patients. Two unrelated single-gland samples from patient 391 (391_67K_SS8_1_2_22205, top) and patient 740 (740__B2_2_1_21963, bottom), processed in different batches display very similar loss patterns deemed to be artifacts due to low input DNA quantities. Some of the most striking recurrent patterns are highlighted by matching color rectangles.

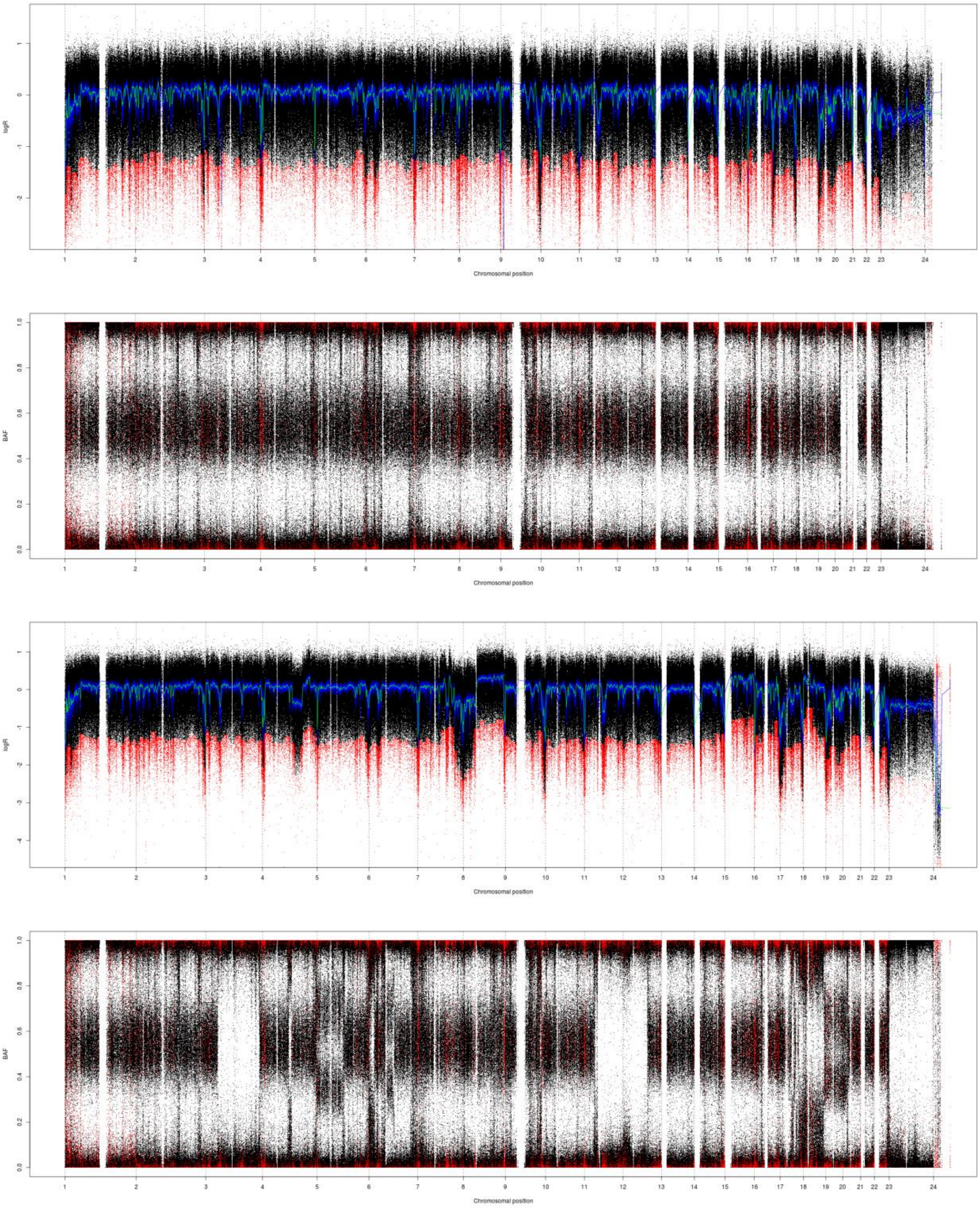

Supplementary Figure 61: Probe filtering. Illustration of probe filtering for samples 391_67K_SS8_1_2_22205 (top 2 plots) and 740__B2_2_1_21963 (bottom 2 plots). logRs are displayed on top and B allele frequencies at the bottom for each sample. Window-based median M values are shown in blue, the median of medians is shown in green. All probes that were filtered out are displayed in red, their logR and BAF values being set to ‘NA’ for this sample.

Segmentation and copy number profiling

In order to ensure that segmentation was compatible across all samples from the same patient, we performed patient-specific joint segmentation using the copynumber R package[2]. For each patient, the filtered logRs and BAFs were segmented independently using the winsorize and multipcf functions, using a high gamma value of 1000 (10000 for patient 256). Missing logR values were imputed using the imputeMissing function. Only heterozygous BAFs were considered, defined as those whose value in the corresponding normal samples were between 0.25 and 0.75 (excluded). BAF values were mirrored so as to stand between 0.5 and 1, and only segments of more than 10 BAF-informative probes were kept. The breakpoints obtained from logR and BAF segmentations were then merged and mean logR and mirrored BAF values were computed for each segment. These values were passed to the ASCAT software[3], by bypassing the ascat.aspcf function, to obtain allele-specific copy numbers.

Fragile site segmentation and breakpoint definition

The genomic coordinates of known fragile sites FHIT and WWOX were retrieved via the ensembl.org website. The logR of both loci were independently joint-segmented with the copynumber package, this time using a low gamma value of 25. For each patient *p*, total copy numbers were predicted for each segment *i* using the following formula:

(1) $CN_i = ((2^{mi} - (1 - P_p)) / P_p) \times 2$

where *mi* is the mean logR of segment *i*, and P_p is the purity of patient *p* as given by the tumor content estimate by ASCAT. Gains and losses were defined using empirical thresholds on the obtained copy number (CN) values obtained per segment: CN values < 0.66 were considered a double loss; 0.66 <= CN < 1.8 values were considered a loss; CN values > 2.3 were considered a gain.

Event matrices

Gains, losses, and cnLOH events were defined as follows. Allele-specific copy numbers from ASCAT were summed to determine total integer copy number for each segment. The overall median total copy number for the sample was then calculated by weighting segment copy numbers by segment size in base pairs. Each segment was then scored as normal (N), loss (L), gain (G), or cnLOH (O) relative to the median copy number. A segment was called as normal if it had the median copy number and both alleles were present, or as cnLOH if it had the median copy number but one allele was absent. It was scored as loss if it had lower than the median copy number, and gain if it had higher. This approach, which evaluates gains and losses relative to the genomic median copy number, was chosen to avoid calling 4N copy number as gain in a genome-doubled (GD) sample, which would produce large numbers of gains and cause spurious clustering of GD samples in the phylogeny.

Further segment filtering

All segments within 10 Mb of telomeres were removed. Segments with less than 100 BAF-informative probes (heterozygous in the normal reference) or spanning less than 10,000 kb were removed. We removed segments likely to correspond to biases due to the low input DNA quantity reproducible across patients (**Supplementary Figure 60**) by comparing the overlap between segments in each patient with segments in all the other patients. If an overlap between segments from two different patients corresponded to 80% or more of the average base pair length of both

segments and the average segment length was less than 10 Mb, the two segments were considered redundant. Segments that were redundant across at least 4 patients were removed. Finally, we filtered out segments in which discontinuous copy number alterations were found across different samples (thus likely to influence phylogeny reconstruction) while no signal difference was visible when manually inspecting BAF and logR values in the relevant samples. A list of all segments removed after manual inspection is given in **Supplementary Data 1**. In addition, the following 9 samples (from 5 patients) were deemed too noisy because of the presence of multiple copy number alterations that could not be identified when manually inspecting logR and BAF values and were also removed from further calculations: 256_42Y_SS18_3_1_22486, 391_67K_SS4_3_2_22223, 391_67K_SS4_3_1_22222, 391_175R_C4_whole_epi__22327, 451_76Z_SS4_3_1_22374, 451_115T_SS6_2_1_22667, 740_78M_SS7_4_2_21934, 740_78M_SS7_1_1_2192, 911_5U_SS6_2_2_22752. **Supplementary Figures 62-69** illustrate the copy number events reported at different stages of the filtering process, while **Supplementary Figure 70** gives an example of manually filtered data.

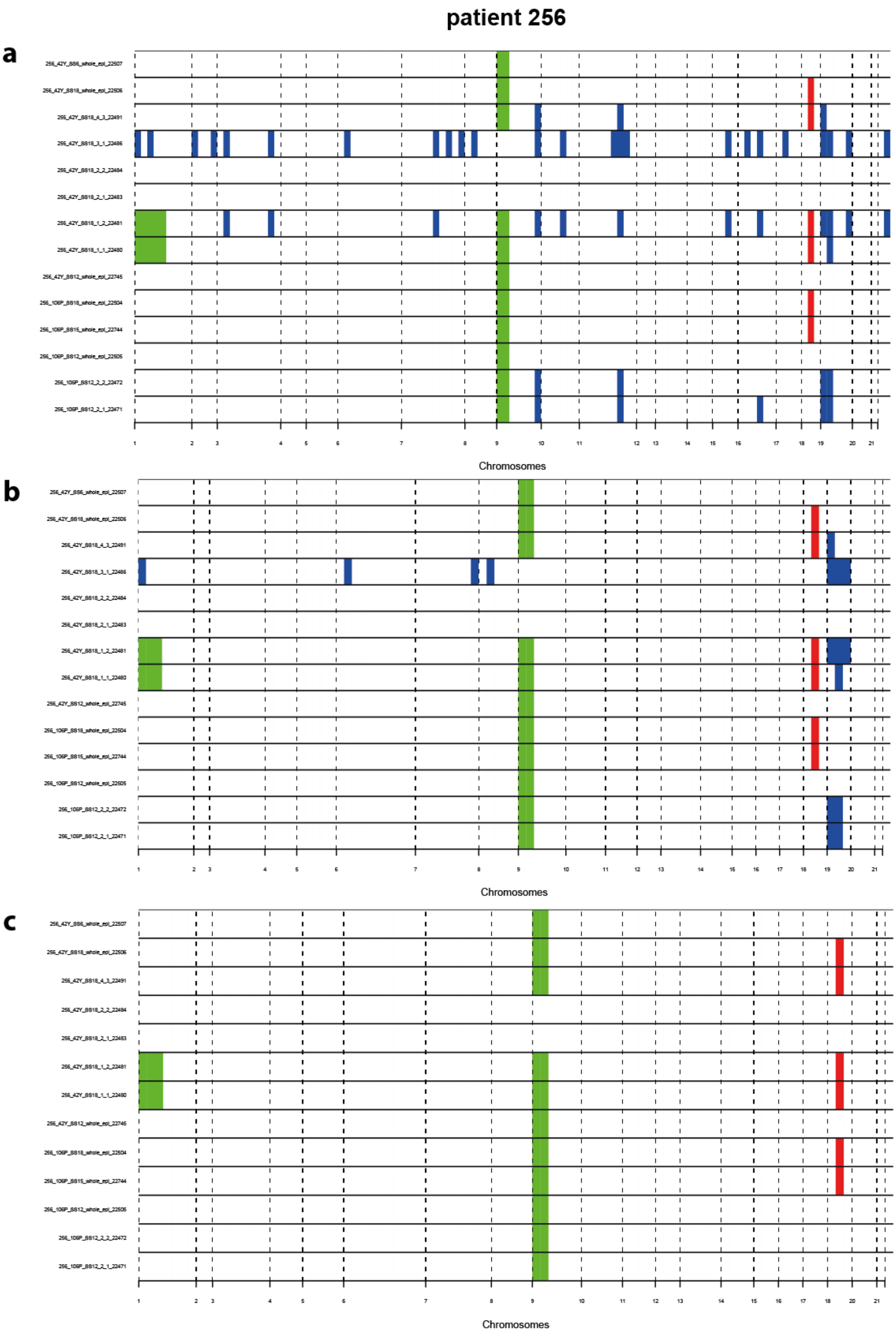

Supplementary Figure 62: Filtering steps (patient 256-P). Blue segments indicate a loss, red ones a gain, green ones a copy-neutral loss of heterozygosity, with white ones showing no alteration. For illustration purposes, all segments have the same size. a) Segments retained prior to filtering on alterations found to recur across patients. b) Segments retained after filtering on alterations found to recur across patients. c) Segments retained after manual inspection and filtering.

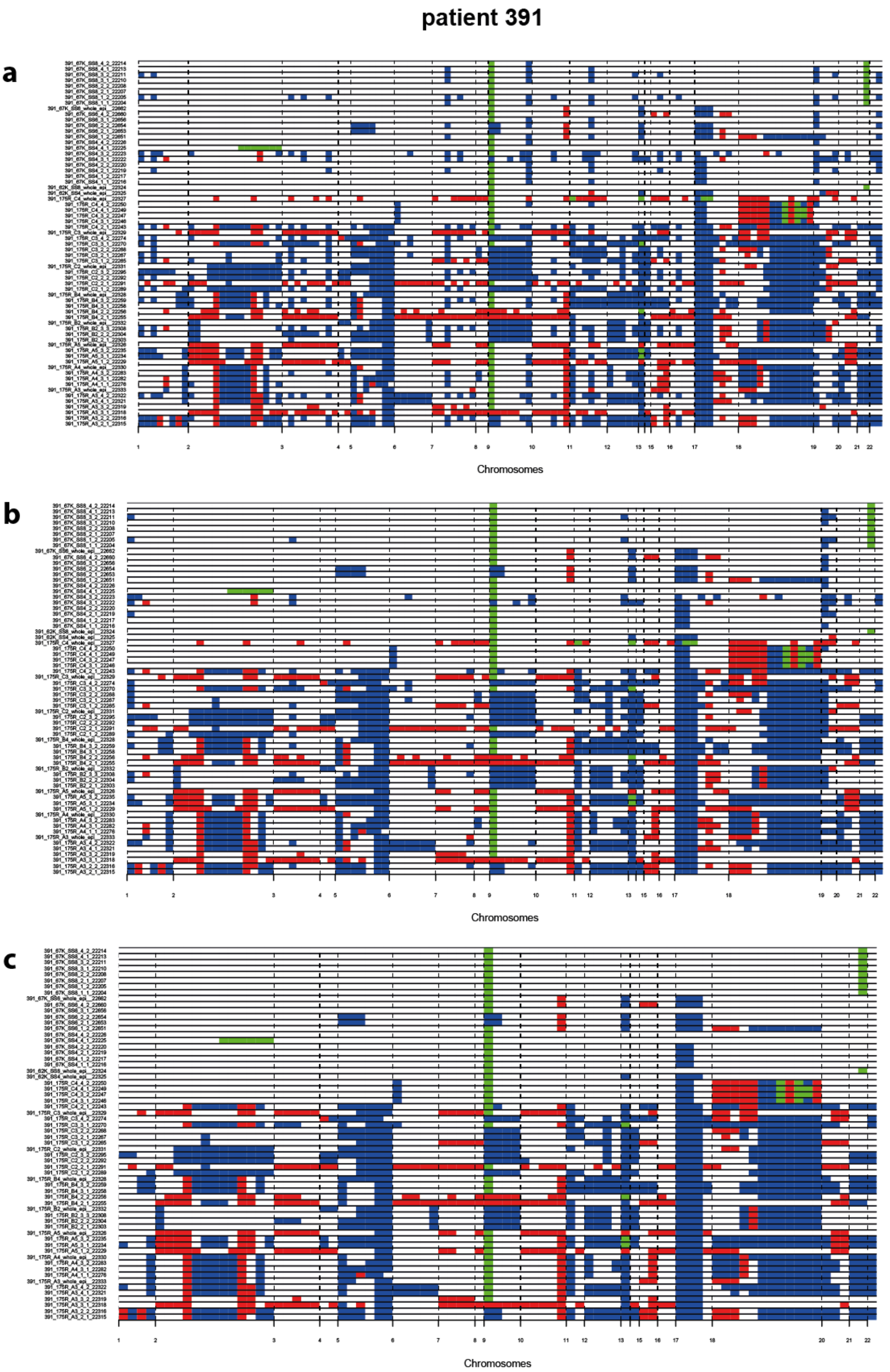

Supplementary Figure 63: Filtering steps (patient 391-P). Blue segments indicate a loss, red ones a gain, green ones a copy-neutral loss of heterozygosity, with white ones showing no alteration. For illustration purposes, all segments have the same size. a) Segments retained prior to filtering on alterations found to recur across patients. b) Segments retained after filtering on alterations found to recur across patients. c) Segments retained after manual inspection and filtering.

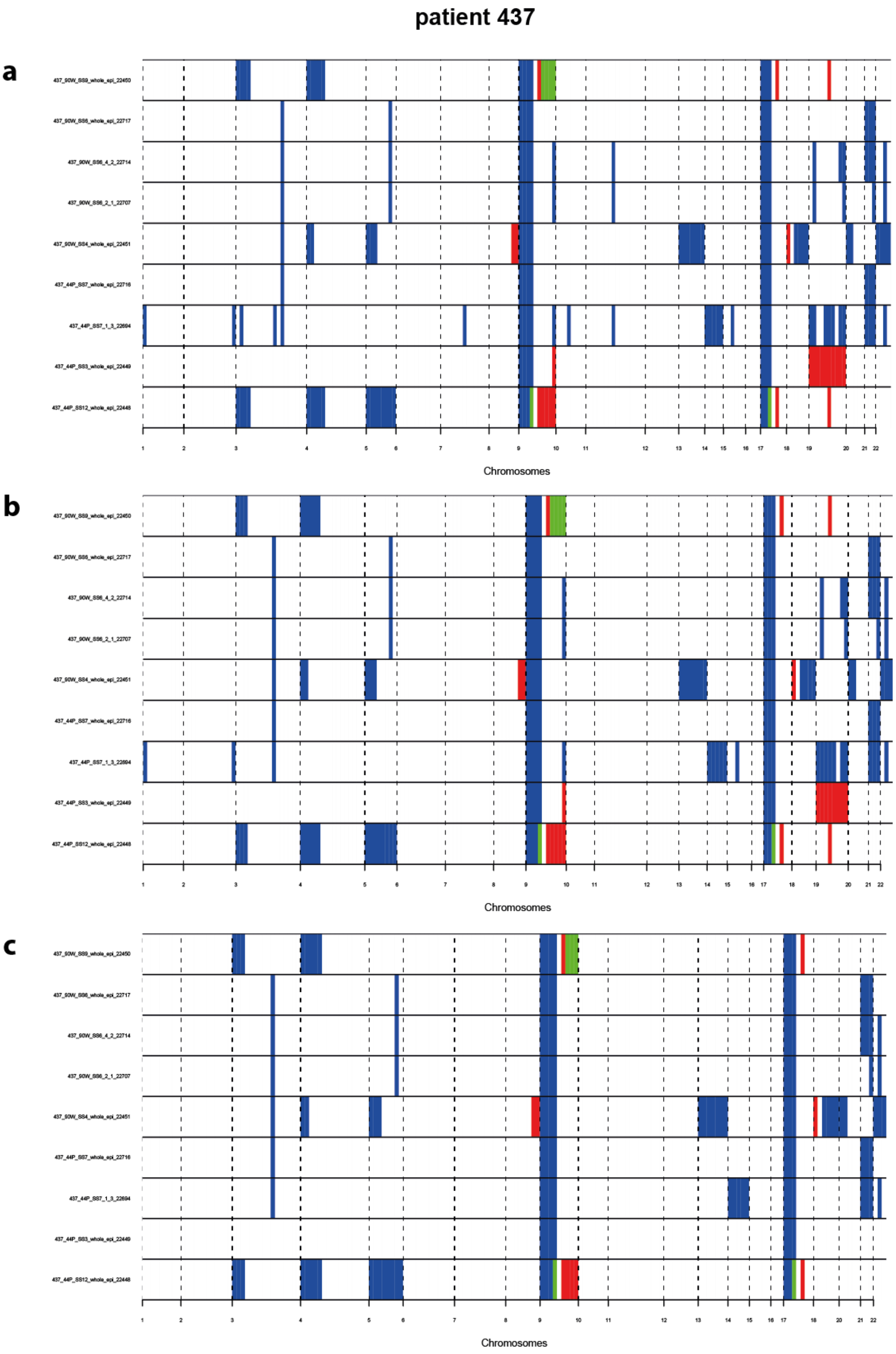

Supplementary Figure 64: Filtering steps (patient 437-NP). Blue segments indicate a loss, red ones a gain, green ones a copy-neutral loss of heterozygosity, with white ones showing no alteration. For illustration purposes, all segments have the same size. a) Segments retained prior to filtering on alterations found to recur across patients. b) Segments retained after filtering on alterations found to recur across patients. c) Segments retained after manual inspection and filtering.

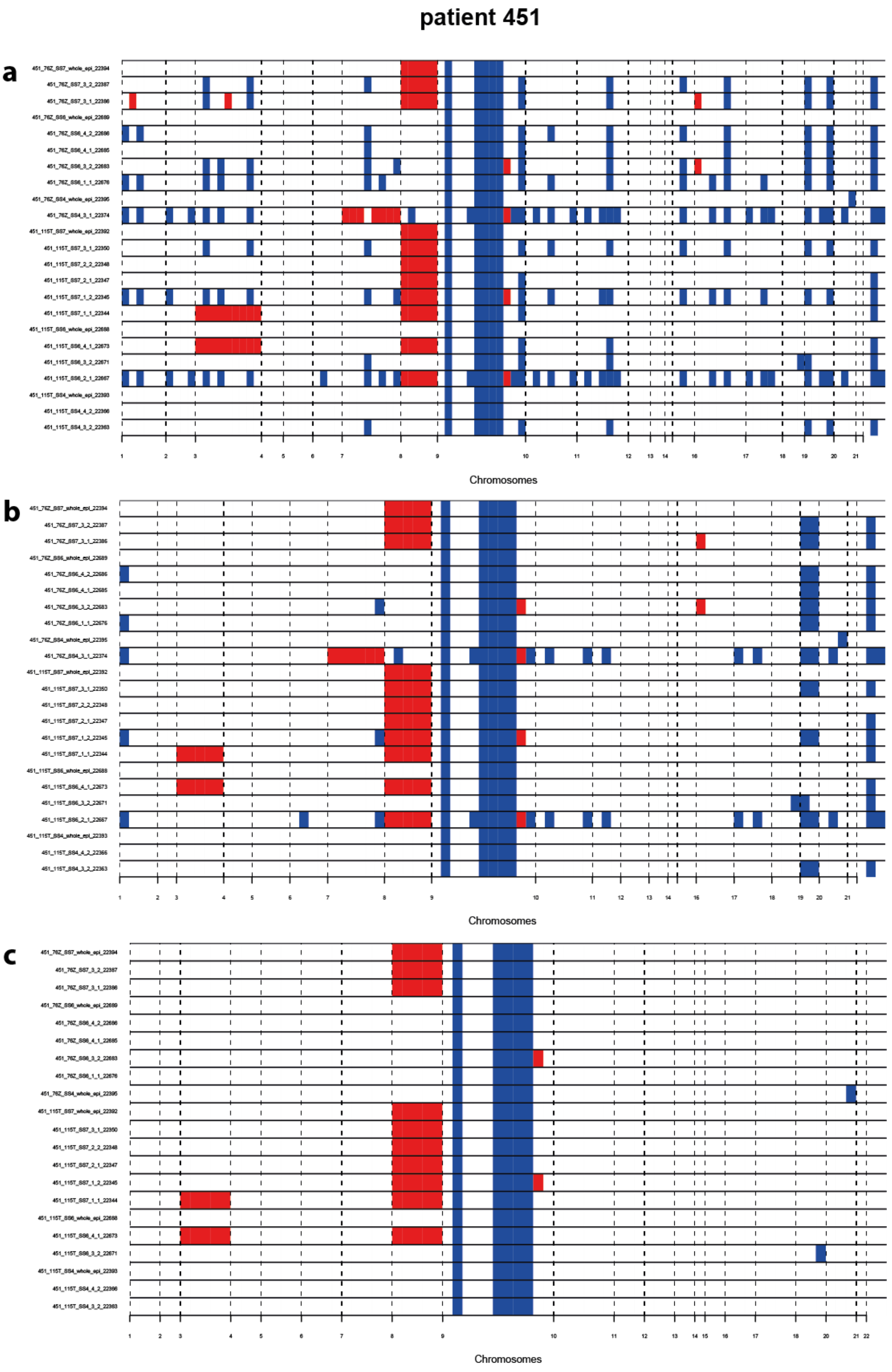

Supplementary Figure 65: Filtering steps (patient 451-NP). Blue segments indicate a loss, red ones a gain, green ones a copy-neutral loss of heterozygosity, with white ones showing no alteration. For illustration purposes, all segments have the same size. a) Segments retained prior to filtering on alterations found to recur across patients. b) Segments retained after filtering on alterations found to recur across patients. c) Segments retained after manual inspection and filtering.

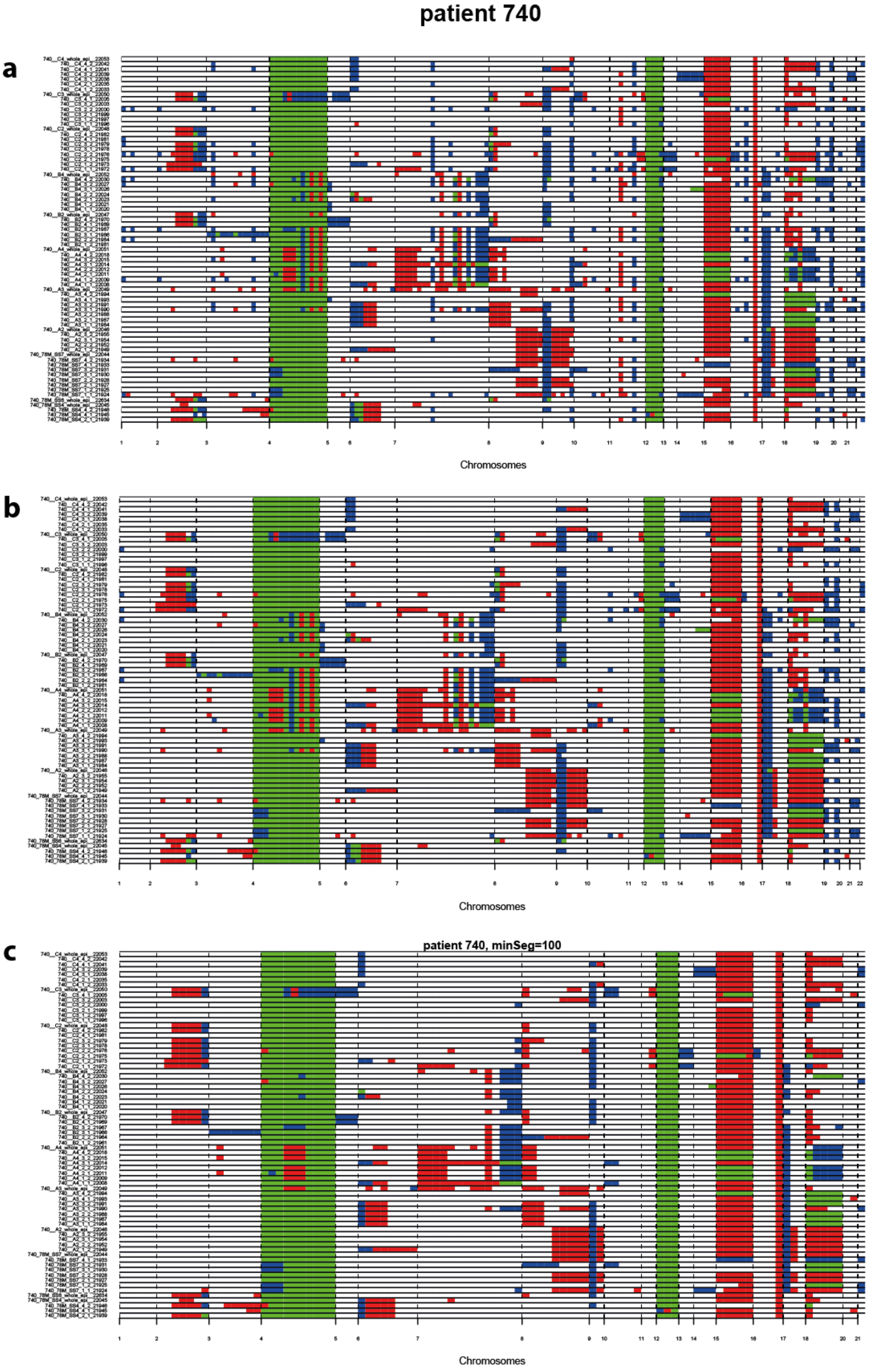

Supplementary Figure 66: Filtering steps (patient 740-P). Blue segments indicate a loss, red ones a gain, green ones a copy-neutral loss of heterozygosity, with white ones showing no alteration. For illustration purposes, all segments have the same size. a) Segments retained prior to filtering on alterations found to recur across patients. b) Segments retained after filtering on alterations found to recur across patients. c) Segments retained after manual inspection and filtering.

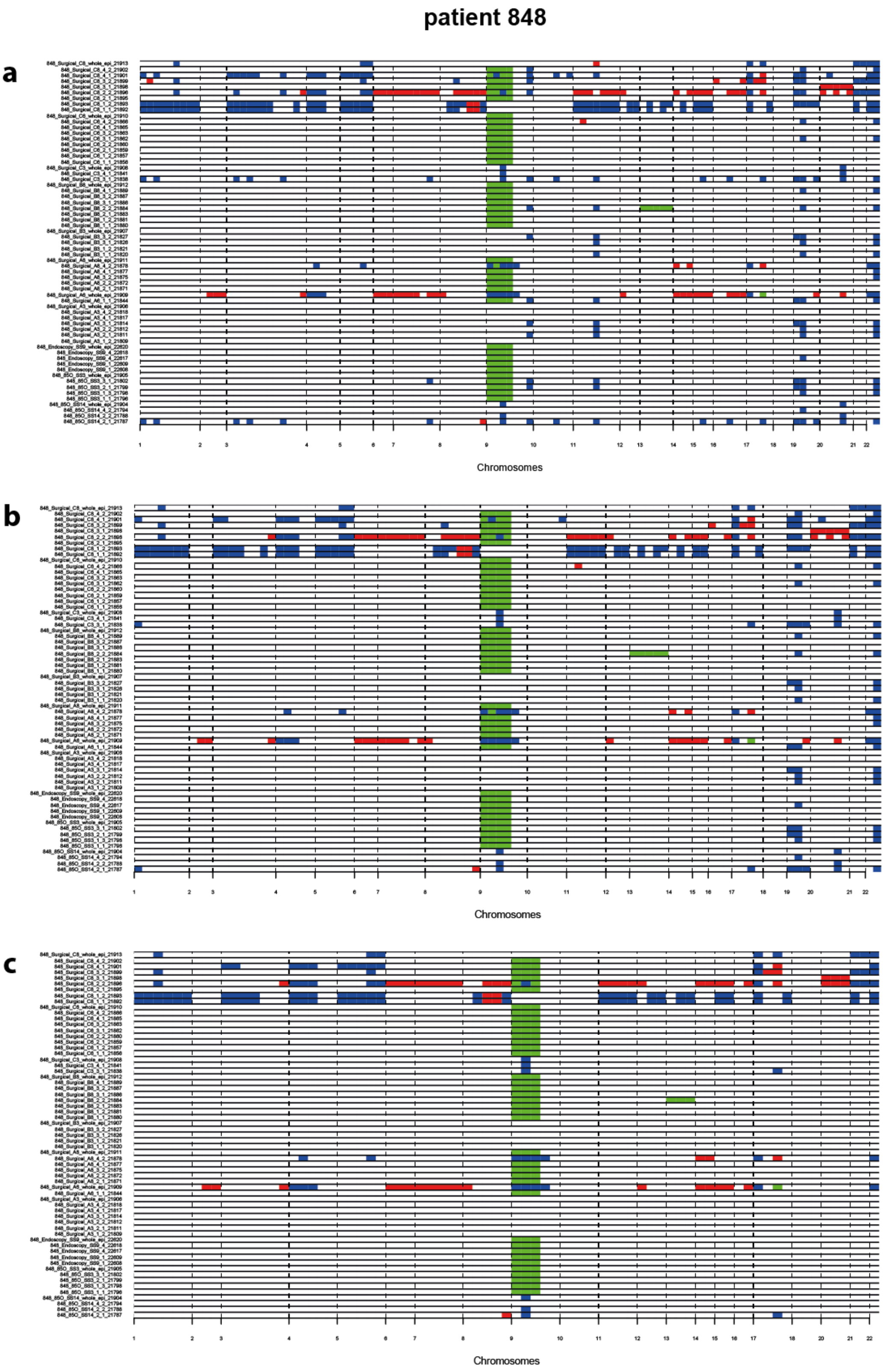

Supplementary Figure 67: Filtering steps (patient 848-P). Blue segments indicate a loss, red ones a gain, green ones a copy-neutral loss of heterozygosity, with white ones showing no alteration. For illustration purposes, all segments have the same size. a) Segments retained prior to filtering on alterations found to recur across patients. b) Segments retained after filtering on alterations found to recur across patients. c) Segments retained after manual inspection and filtering.

patient 848

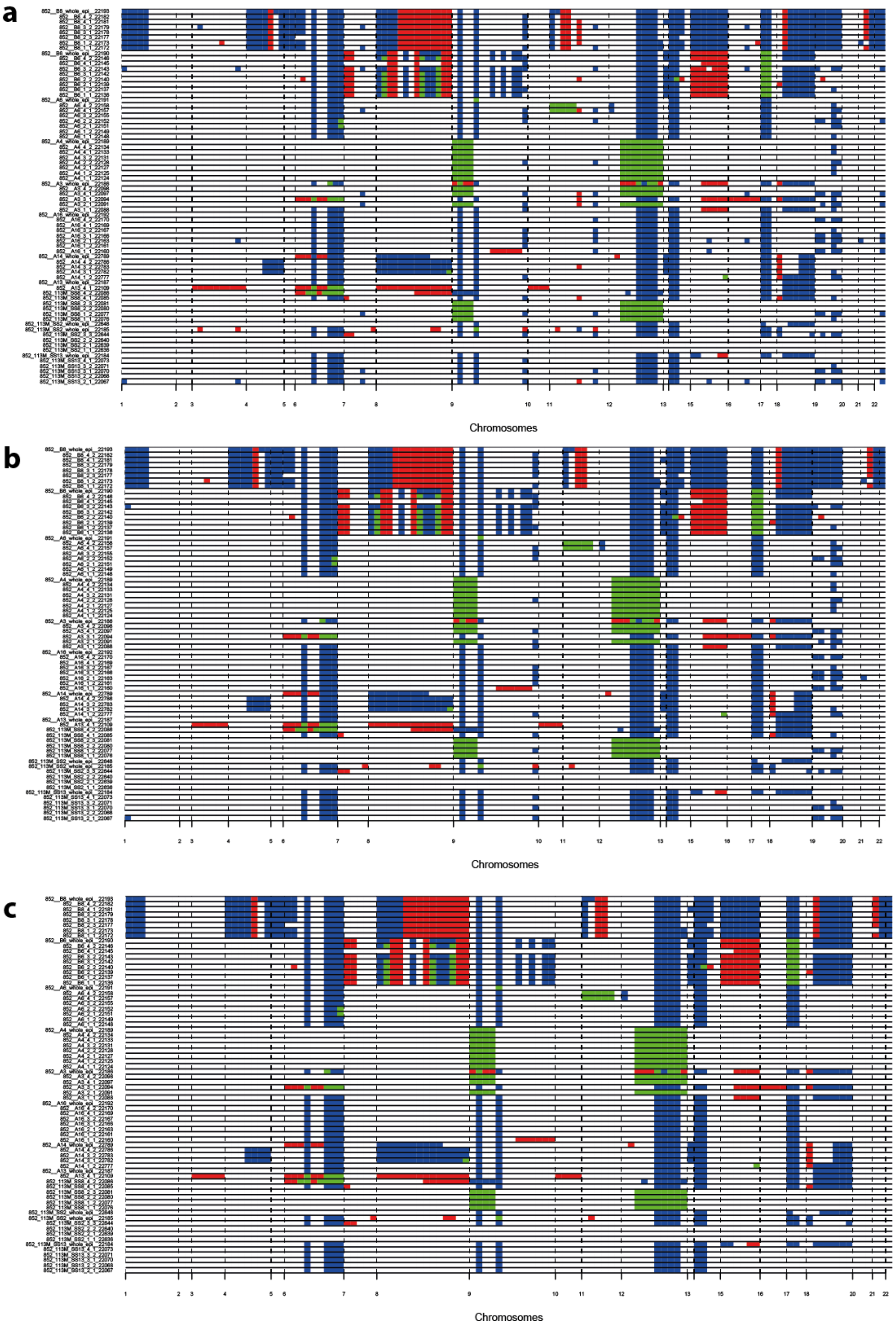

Supplementary Figure 68: Filtering steps (patient 852-P). Blue segments indicate a loss, red ones a gain, green ones a copy-neutral loss of heterozygosity, with white ones showing no alteration. For illustration purposes, all segments have the same size. a) Segments retained prior to filtering on alterations found to recur across patients. b) Segments retained after filtering on alterations found to recur across patients. c) Segments retained after manual inspection and filtering.

patient 911

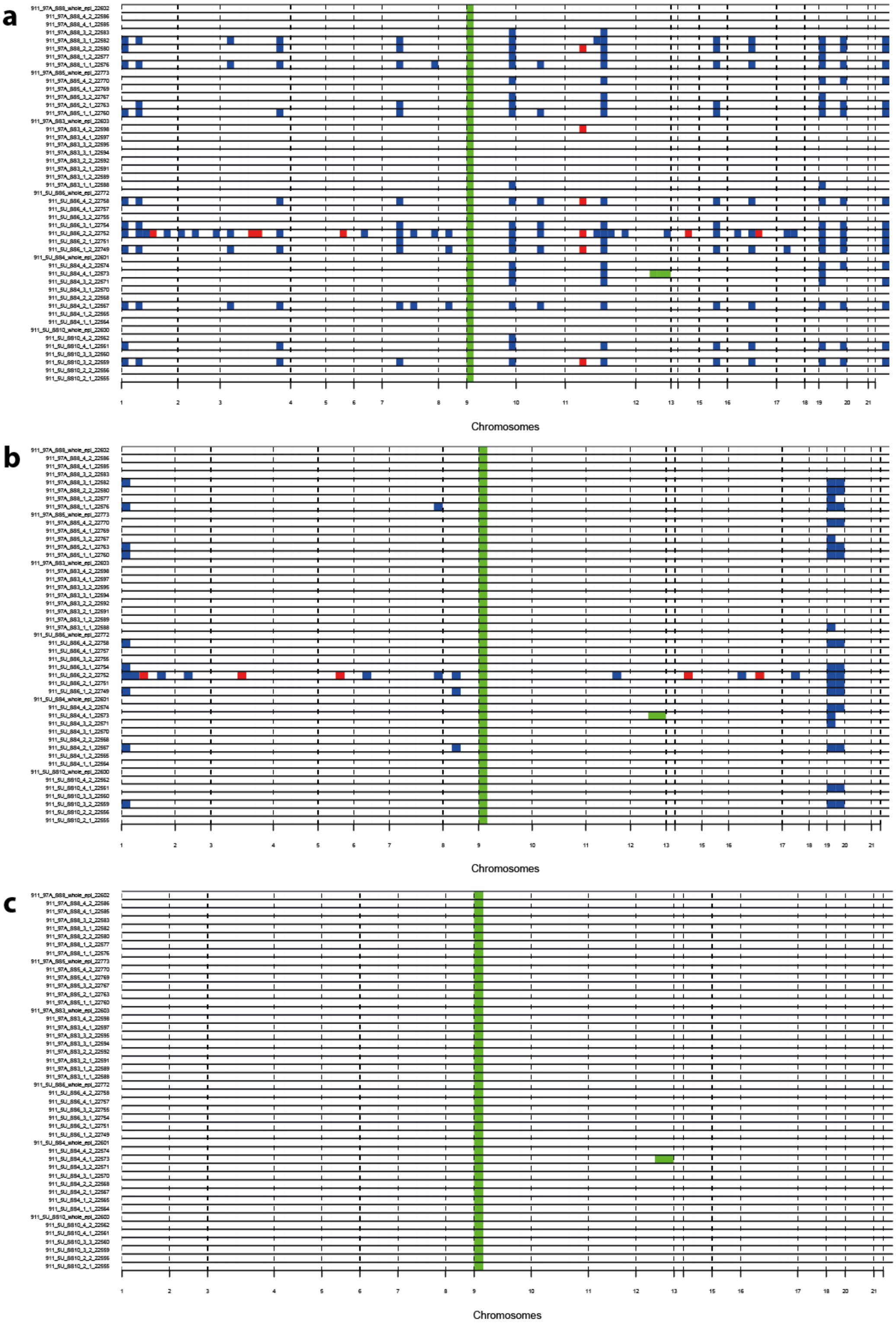

Supplementary Figure 69: Filtering steps (patient 911-NP). Blue segments indicate a loss, red ones a gain, green ones a copy-neutral loss of heterozygosity, with white ones showing no alteration. For illustration purposes, all segments have the same size. a) Segments retained prior to filtering on alterations found to recur across patients. b) Segments retained after filtering on alterations found to recur across patients. c) Segments retained after manual inspection and filtering.

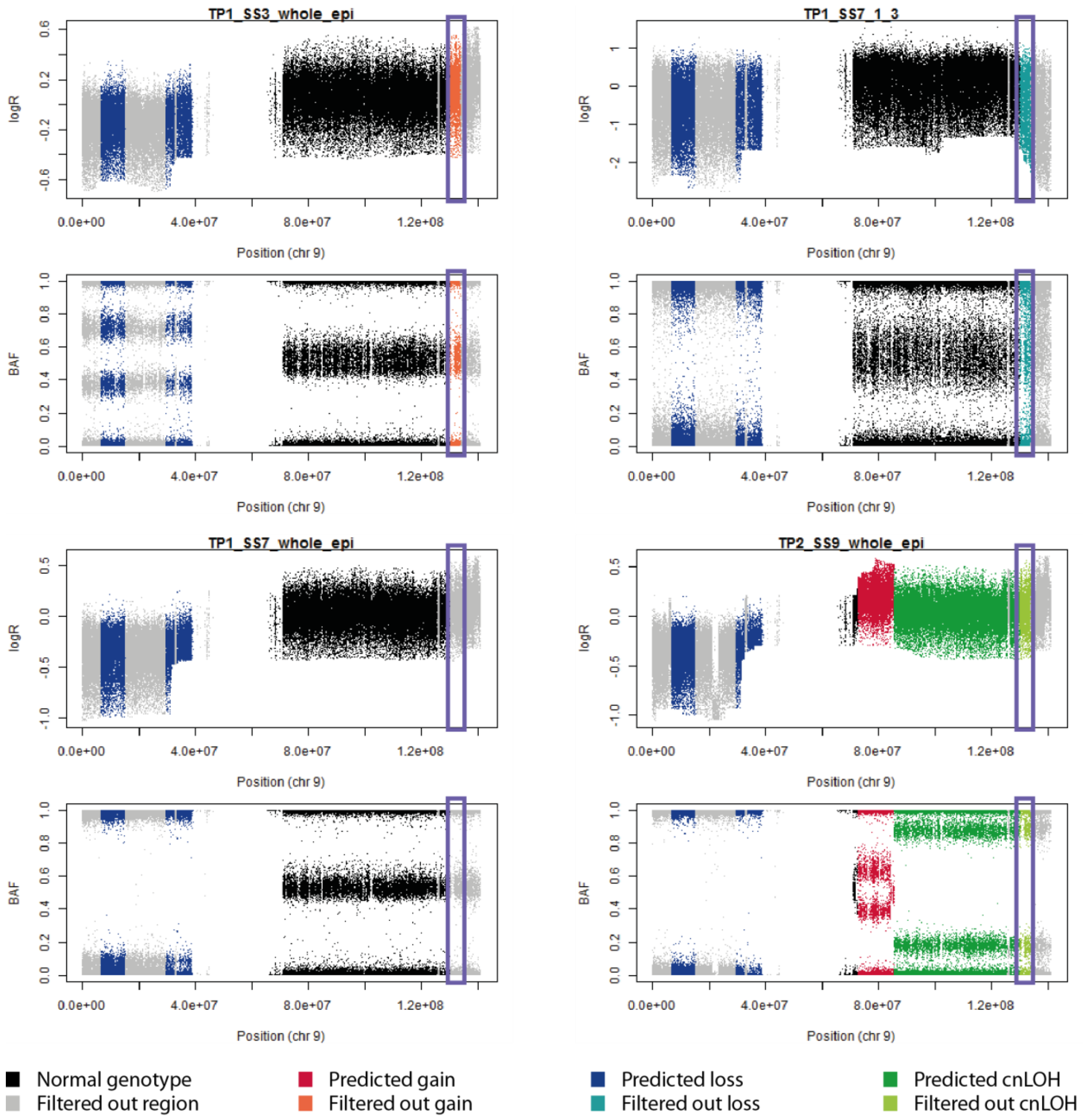

Supplementary Figure 70: Example of manual filtering on progressor patient 437-NP's chromosome 9. LogR and BAF values of 4 different regions are plotted along chromosome 9. Gray dots belong to segments that were filtered out of the analysis. One segment near the chromosome end (coordinates 130132392-133977901) was filtered out manually and is highlighted by rectangular boxes. A gain, highlighted in orange, was predicted in this segment for sample TP1_SS3_whole_epi (top left) and a loss, highlighted in cyan, was predicted in sample TP1_SS7_1_3 (top right), with no evidence of a shift in BAF values such as is observable in the loss pattern on the q arm (highlighted in blue). Both samples resemble TP1_SS7_whole_epi (bottom left), in which no alteration was predicted and the segment was filtered out in all samples for this patient. A genuine-looking copy-neutral loss of heterozygosity (cnLOH) is thus be filtered out in sample TP2_SS9_whole_epi. However, due to the breakpoint-oriented method we are using, this would not impair phylogenetic reconstruction**.**

Allele phasing

Starting with a matrix of allele-specific copy numbers per segment per sample, a copy number (CN) "event" was defined as an abnormal genotype in one segment in one sample. All events were computed relative to baseline ploidy so a total copy number of 2 would be considered a loss if the baseline ploidy was 4. All samples had paired normal information.

For phylogenetic analysis, we needed to determine how many copies of each original allele were present in each segment, and define these alleles in a consistent way across adjacent segments. Our strategy was to assume that, when two segments of identical copy number (CN) call were adjacent, they represented gain or loss of the same haplotype unless there was substantial evidence supporting gain or loss of different haplotypes (**Supplementary Fig. 71a**, leftmost panel). This assumption minimizes calling of spurious breakpoints.

We began by defining a “seed” segment from which to build haplotypes, preferring segments likely to be highly informative (**Supplementary Fig. 71a**, center left panel). The segment was chosen to maximize the following score: number of contiguous segments with the same CN call, times log10 of the number of heterozygous SNPs in the segment. We then determined, for each heterozygous SNP in the seed segment, whether it had increased or decreased BAF relative to normal (the patient's constitutive genotype). The sample with the greatest mean BAF difference between its SNPs and normal was chosen as the reference sample as it was presumed to offer the clearest signal.

We divided the heterozygous SNPs in the seed segment into those whose BAFs were increased in the reference sample and those whose BAFs were decreased; the decreased SNPs were arbitrarily designated as corresponding to haplotype A and the increased SNPs to haplotype B.

We then examined this segment in all other samples from the same patient. In segments showing a CN event opposite to the one in the reference sample (loss or copy-neutral LOH versus gain), the increased SNPs were considered as haplotype A and the decreased SNPs as haplotype B. We assumed that the increased haplotype in each sample displaying the same CN event was the same as in the reference sample, unless the informative SNPs supporting a reversed assignment were >1.5x greater than the number of SNPs supporting the reference-sample assignment, in which case the sample was designated as “swapped.”, i.e. affecting the other allele compared to the reference sample. The threshold of 1.5x was chosen empirically to reduce the frequency of apparently spurious transitions between, for example, loss of A and loss of B in noisy data.

We then counted, for each informative SNP, the number of samples in which that SNP was associated with the same haplotype (that is, varied in the same direction as in the reference sample for a non-swapped sample, or in the opposite direction for a swapped sample). The maximum of this number across SNPs defined the maximum phasing support (MPS). We based our phasing decisions only on SNPs which were associated with the same haplotype at least 0.75 * MPS proportion of the time. For example, if we examined 10 samples and the SNP with the highest consistency of haplotype assignment was associated with the same allele in 8 of them, the MPS was 8, and we considered all SNPs associated with the same haplotype in at least 0.75 * 8 = 6 samples to

be phased to that haplotype. Based on these SNPs, we assigned a phased genotype to each sample according to the reference sample phasing, the copy number event detected in each sample, and whether it was considered as swapped.

Once phase was determined for the seed segment, we extended phase inference in both directions down the chromosome until all contiguous segments with the same CN had been phased (**Supplementary Fig. 71a**, center right panel). It is not possible to infer phase relationships among non-contiguous segments from these data. For each new segment, we found heterozygous SNPs with increased or decreased BAFs as before. We defined the A haplotype as SNPs decreased in normal samples and increased in previously swapped samples, and the B haplotype as the reverse. An additional filtering step was added in which we computed the mirrored BAF frequencies of heterozygous probes in the previous and current segments. If the current segment's frequencies were significantly different from those in the previous segment (defined as $p<0.1$ in a t-test) we did not merge the current segment with the previous segment; the significant t-test was taken as evidence that these represented two different CN events, perhaps present in different fractions of the cells, rather than a single event.

The A and B probes were then defined as those with MPS * 0.75 phasing support as before. When no samples passed the t-test filtering, all samples sharing a CN event with the previous segment were used for phasing. This was done to favor consistency and avoid creating spurious breakpoints as much as possible. Finally, the "swapped" status of each sample was re-evaluated based on the chosen probes, and the phasing was propagated if there was at least one sample with an identical CN event in the current segment. When no further phasing propagation was possible, the process was restarted by defining a new seed segment on the remaining unphased segments (**Supplementary Fig. 71a**, rightmost panel).

Associations between all phased SNPs and alleles were recorded (**Supplementary Fig. 71b-c**) and used to produce phased allele-specific copy number matrices and phased event matrices. Copy number matrices corresponded, for each patient, to two matrices of copy number per chromosomal segment, one for the allele defined as 'A' by our phasing method, one for allele 'B'. In the phased event matrices, losses, gains and cnLOH events were associated with a specific allele. Final phased copy number profiles are displayed in **Supplementary Figures 72-79**.

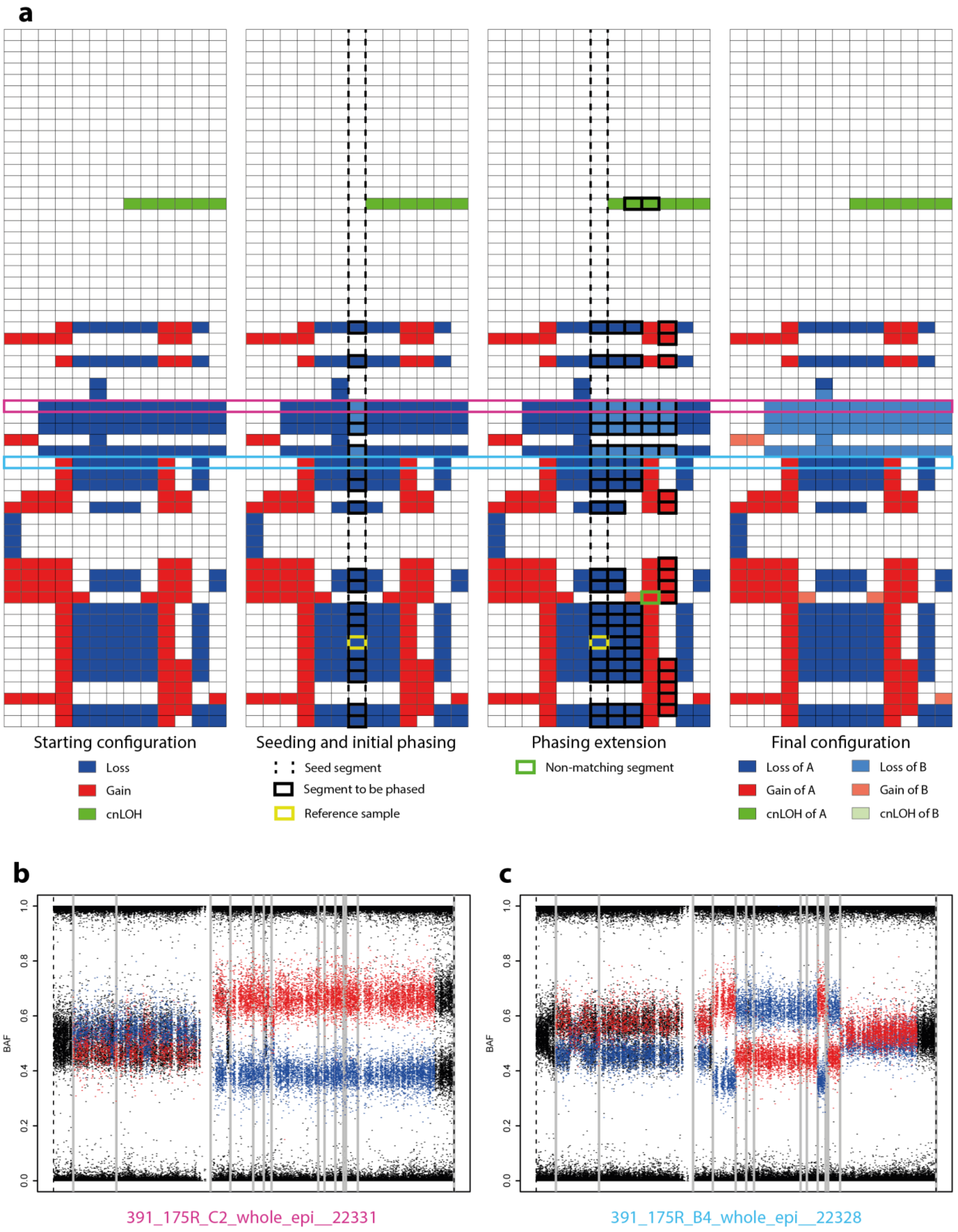

Supplementary Figure 71: Allele phasing example (patient 391-P, chromosome 2). a) Different steps of the phasing process in a chromosome made of 13 segments (columns) in 62 samples (rows). From the initially unphased CNV event matrix, the seed segment is selected as the one showing the best potential for reliable phasing propagation (here segment 7). The reference sample is be selected as the one with the largest split between alleles, the probes showing a decrease compared to normal will be defined as the reference A allele. Segments in which SNPs behave contrarily to the reference sample are "swapped" and the best matching SNPS are phased to each allele. The phasing is propagated in both directions in stepwise fashion. Segments that display the same event as the previous segment in a given sample are selected to define the SNPs associated to each alleles. If a mismatch is detected due to discrepancy in mirrored B allele frequencies with the previous segments or because the phasing is not in agreement with other samples being phased, the non-matching segment is excluded from the SNP to allele association phase (example given in segment 10 – 3rd step of the propagation process). Segments are then "swapped" if not matching the SNP to allele association and the best matching SNPS are phased to each allele. Finally, CNV events (and allele-specific copy numbers) are phased using the information regarding which SNPs are associated to which allele. b-c) Example of losses impacting different alleles in the same patient. Samples 391_175R_C2_whole_epi__22331 (panel b and sample highlighted in magenta in panel a) and 391_175R_B4_whole_epi__22328 (panel c and sample highlighted in cyan in panel a) appear to have been impacted by different events and the losses spanning segments 5 to 9 occur in different alleles, which could not be determined prior to phasing.

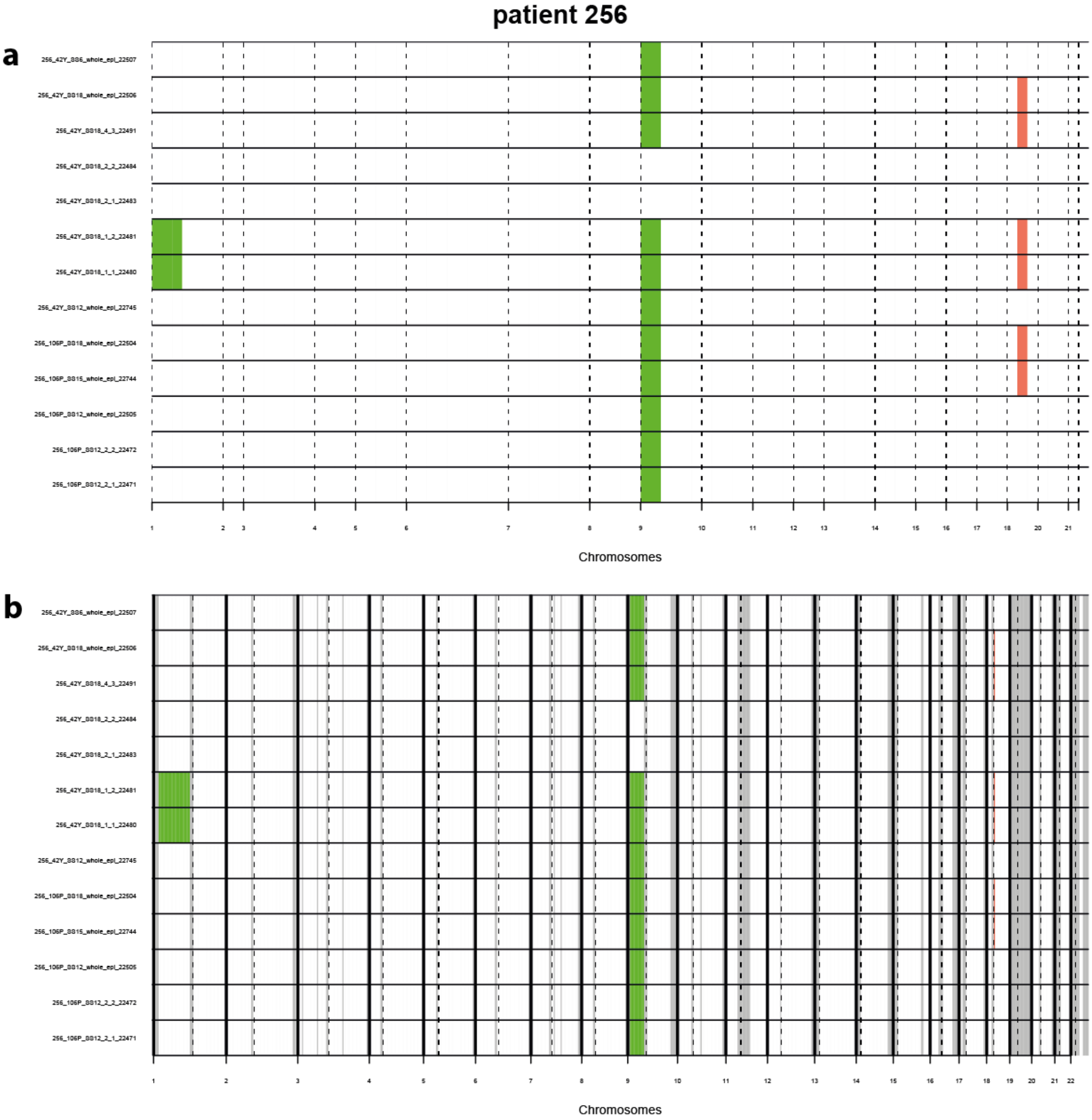

Supplementary Figure 72: Final phased profile (patient 256-NP). Blue segments indicate a loss, red ones a gain, green ones a copy-neutral loss of heterozygosity, with white ones showing no alteration. Darker colours indicate alterations on allele A, lighter ones indicate alterations on allele B (alleles defined arbitrarily). a) Segment-based profile: all segments are displayed as having the same size. b) Sized profile: Alteration are shown on a genome-scale using the dominant copy-number state for each minor cytoband. Grey areas indicate areas where profiling was not possible due to previous filtering steps.

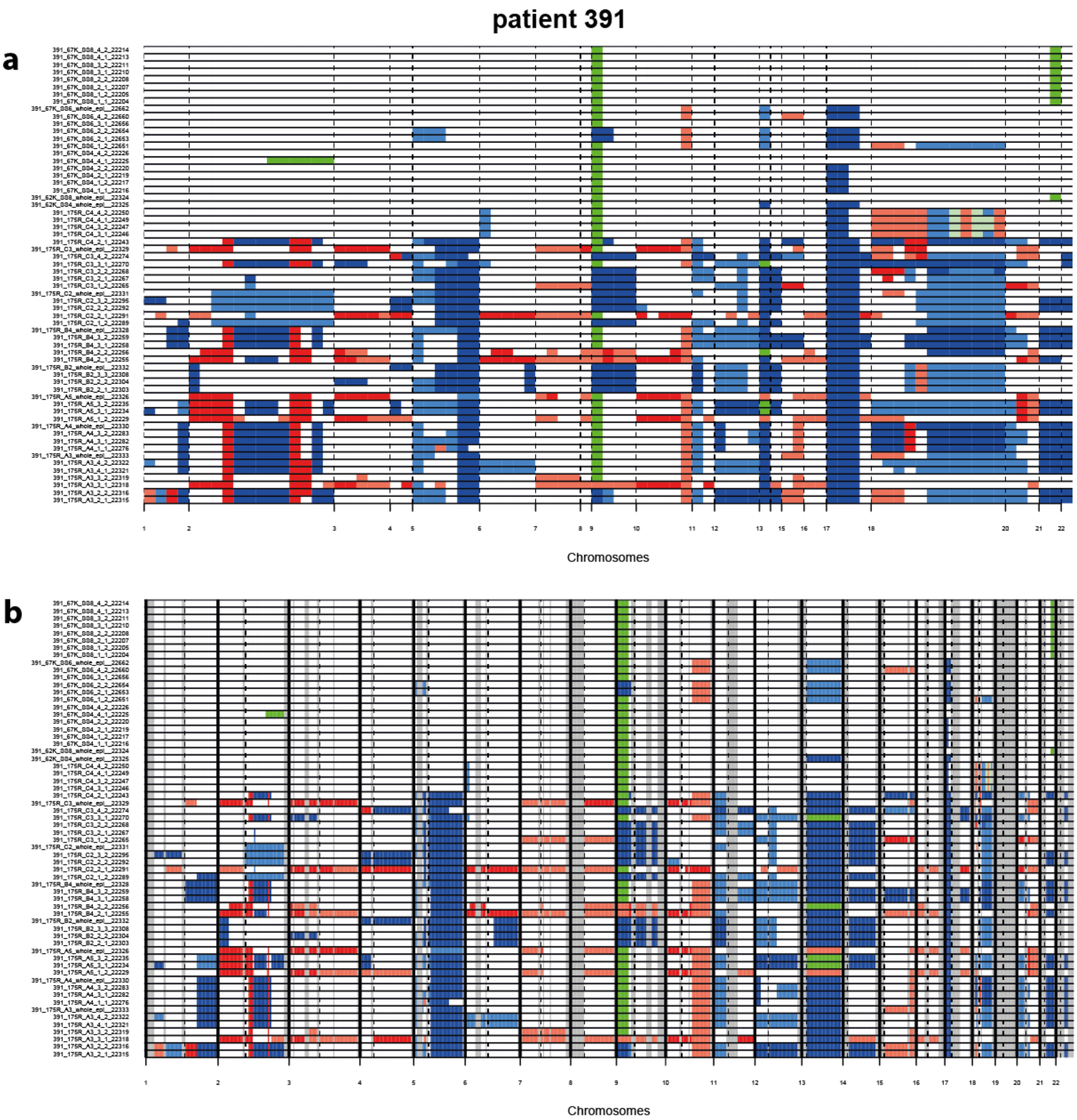

Supplementary Figure 73: Final phased profile (patient 391-P). Blue segments indicate a loss, red ones a gain, green ones a copy-neutral loss of heterozygosity, with white ones showing no alteration. Darker colours indicate alterations on allele A, lighter ones indicate alterations on allele B (alleles defined arbitrarily). a) Segment-based profile: all segments are displayed as having the same size. b) Sized profile: Alteration are shown on a genome-scale using the dominant copy-number state for each minor cytoband. Grey areas indicate areas where profiling was not possible due to previous filtering steps.

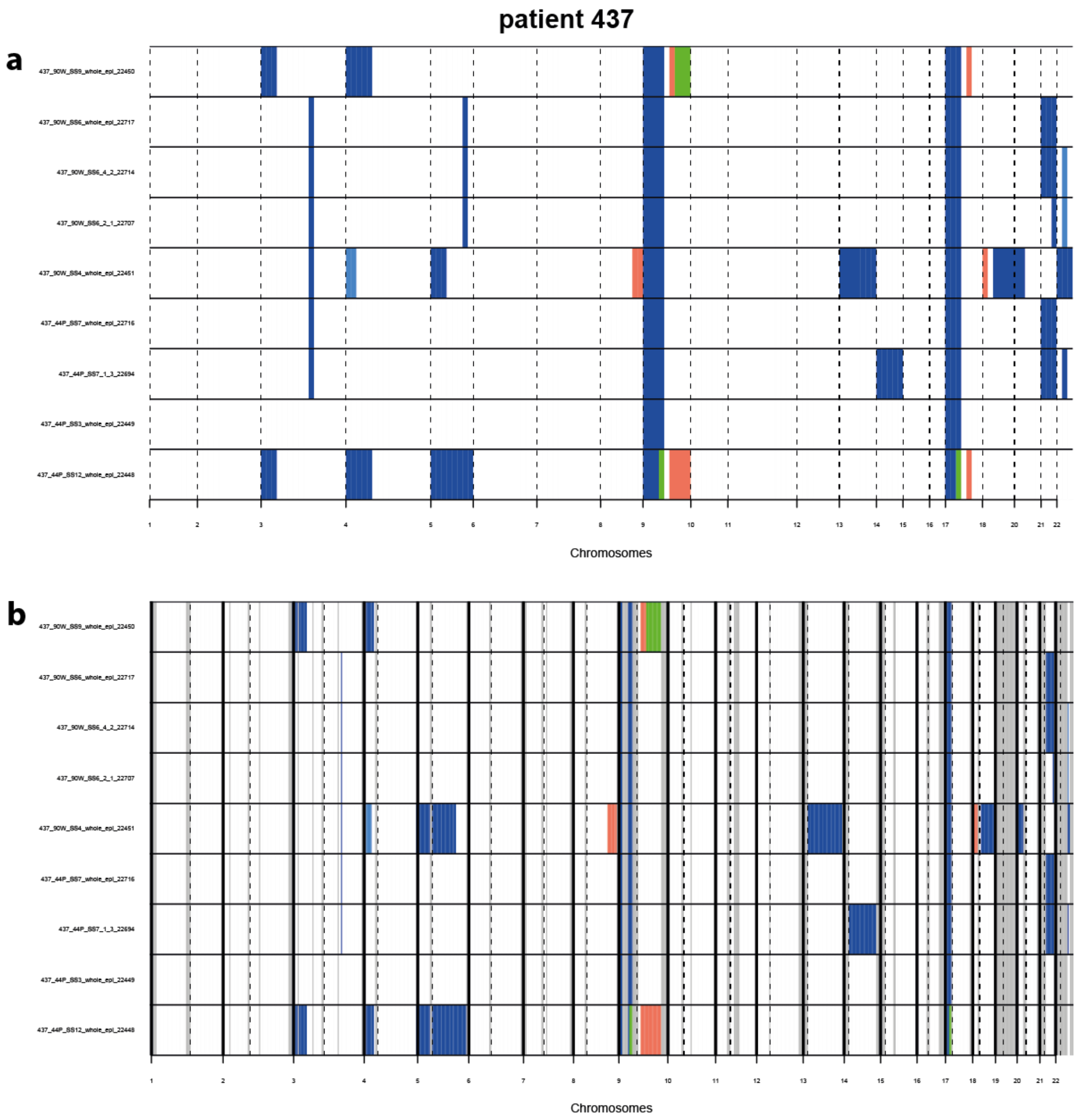

Supplementary Figure 74: Final phased profile (patient 437-NP). Blue segments indicate a loss, red ones a gain, green ones a copy-neutral loss of heterozygosity, with white ones showing no alteration. Darker colours indicate alterations on allele A, lighter ones indicate alterations on allele B (alleles defined arbitrarily). a) Segment-based profile: all segments are displayed as having the same size. b) Sized profile: Alteration are shown on a genome-scale using the dominant copy-number state for each minor cytoband. Grey areas indicate areas where profiling was not possible due to previous filtering steps.

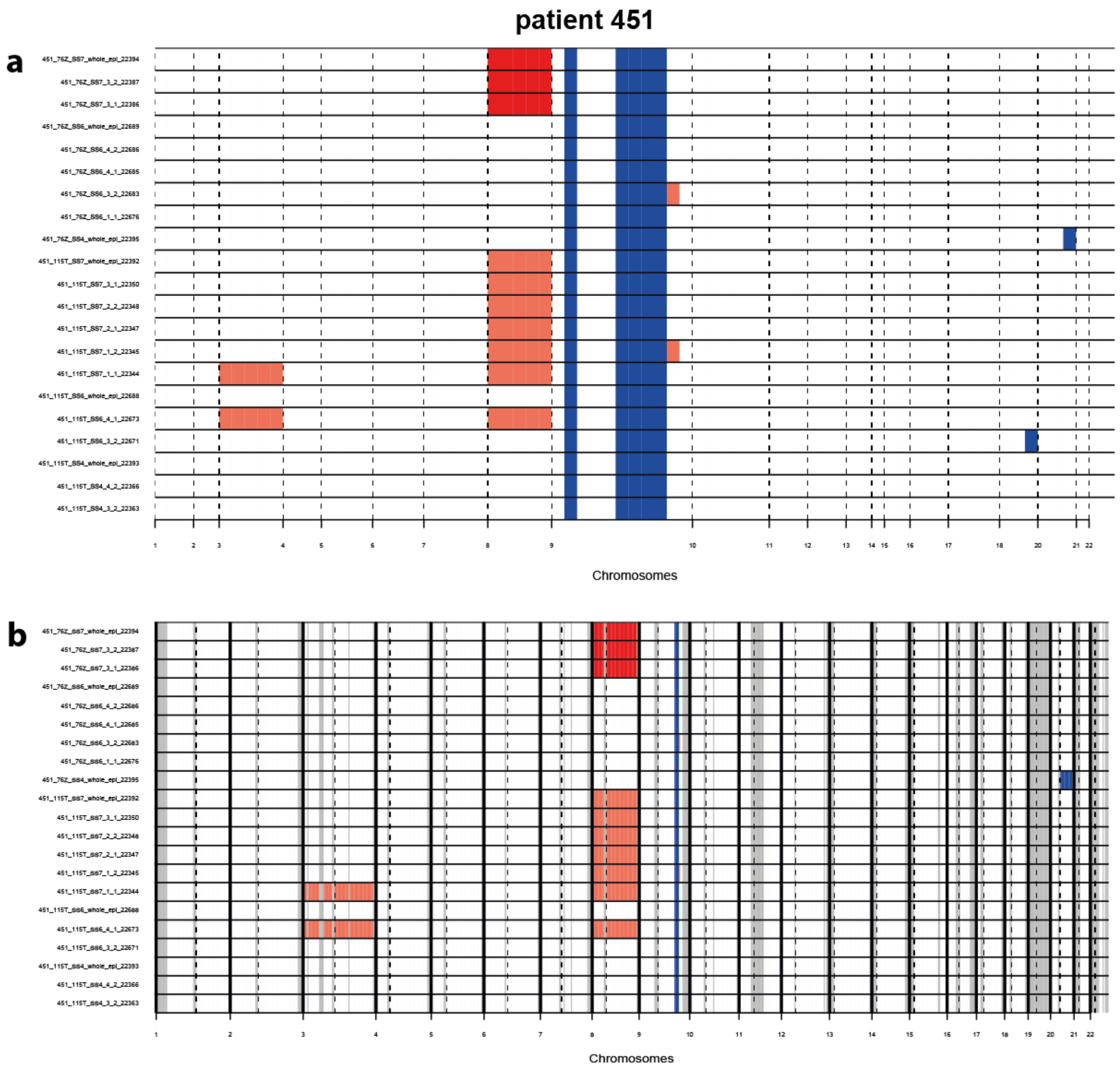

Supplementary Figure 75: Final phased profile (patient 451-NP). Blue segments indicate a loss, red ones a gain, green ones a copy-neutral loss of heterozygosity, with white ones showing no alteration. Darker colours indicate alterations on allele A, lighter ones indicate alterations on allele B (alleles defined arbitrarily). a) Segment-based profile: all segments are displayed as having the same size. b) Sized profile: Alteration are shown on a genome-scale using the dominant copy-number state for each minor cytoband. Grey areas indicate areas where profiling was not possible due to previous filtering steps.

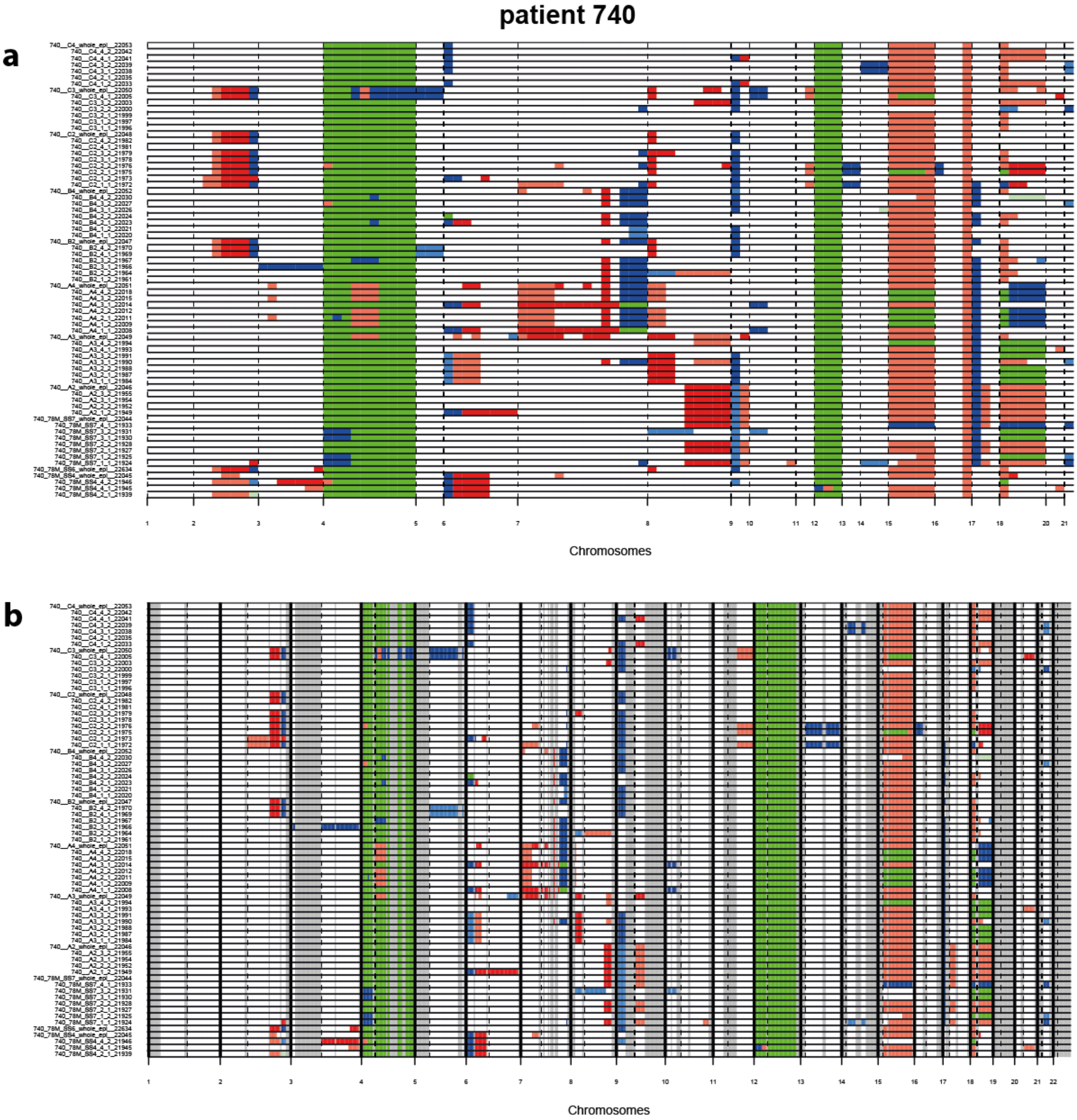

Supplementary Figure 76: Final phased profile (patient 740-P). Blue segments indicate a loss, red ones a gain, green ones a copy-neutral loss of heterozygosity, with white ones showing no alteration. Darker colours indicate alterations on allele A, lighter ones indicate alterations on allele B (alleles defined arbitrarily). a) Segment-based profile: all segments are displayed as having the same size. b) Sized profile: Alteration are shown on a genome-scale using the dominant copy-number state for each minor cytoband. Grey areas indicate areas where profiling was not possible due to previous filtering steps.

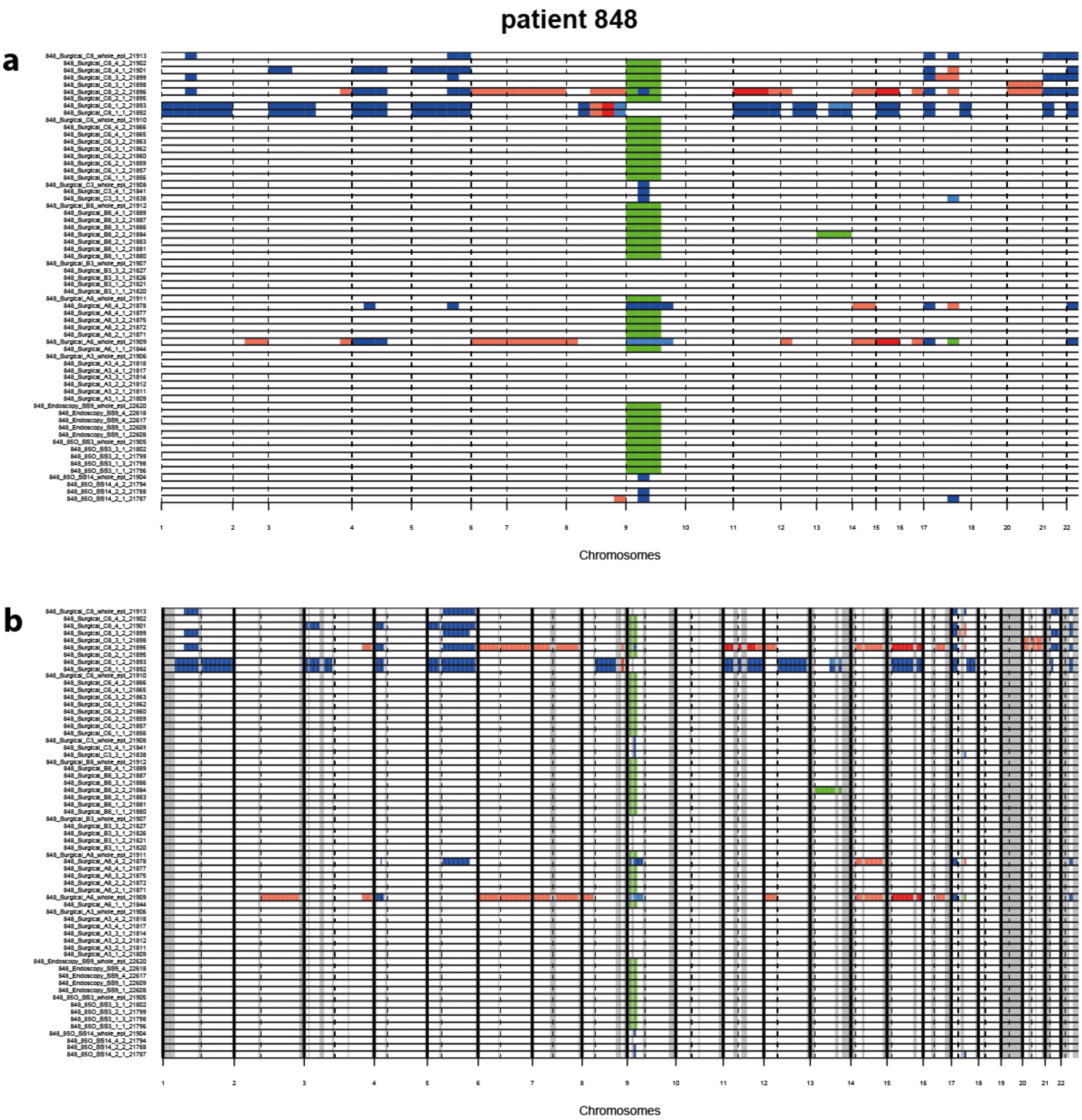

Supplementary Figure 77: Final phased profile (patient 848-P). Blue segments indicate a loss, red ones a gain, green ones a copy-neutral loss of heterozygosity, with white ones showing no alteration. Darker colours indicate alterations on allele A, lighter ones indicate alterations on allele B (alleles defined arbitrarily). a) Segment-based profile: all segments are displayed as having the same size. b) Sized profile: Alteration are shown on a genome-scale using the dominant copy-number state for each minor cytoband. Grey areas indicate areas where profiling was not possible due to previous filtering steps.

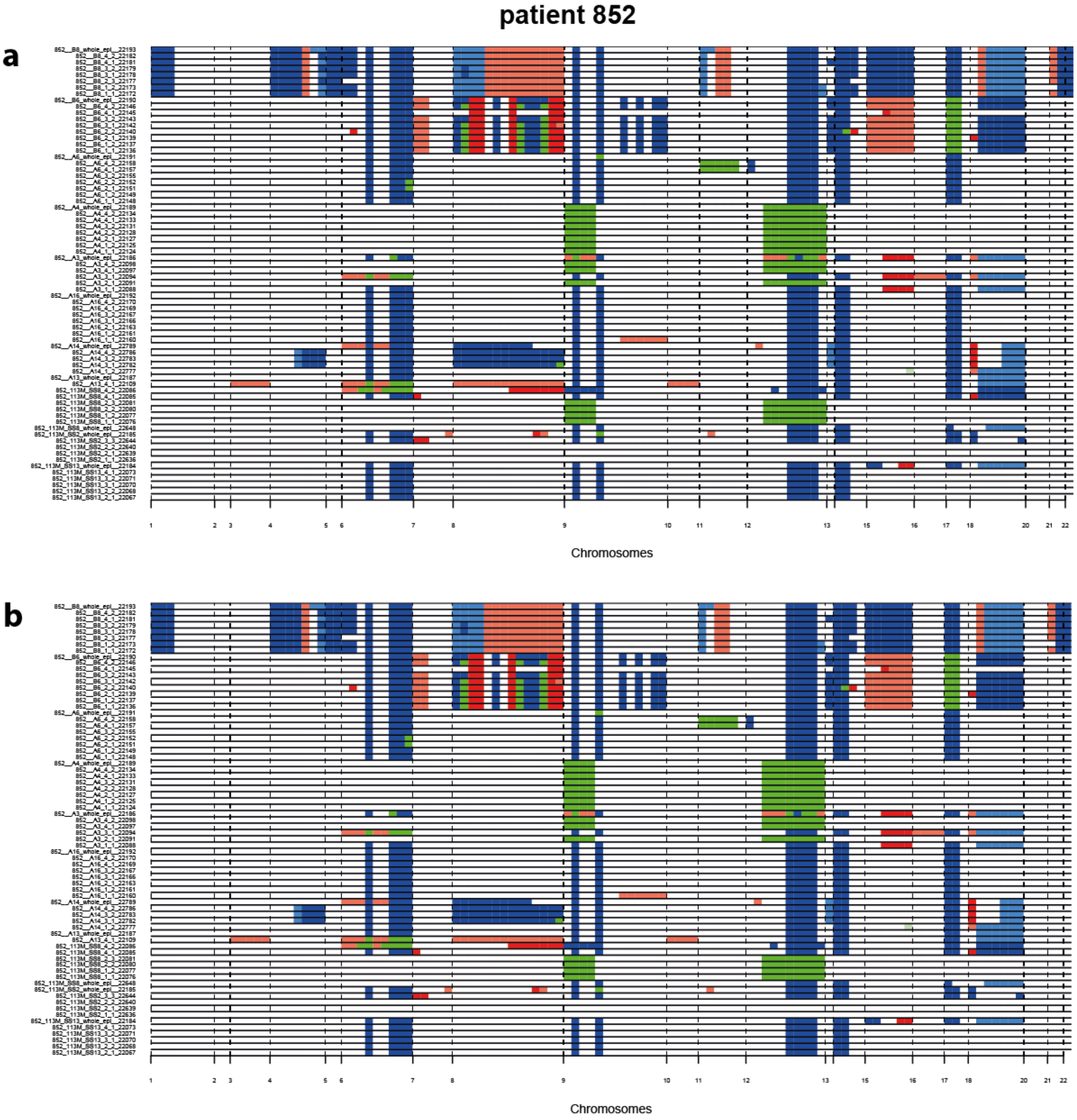

Supplementary Figure 78: Final phased profile (patient 852-P). Blue segments indicate a loss, red ones a gain, green ones a copy-neutral loss of heterozygosity, with white ones showing no alteration. Darker colours indicate alterations on allele A, lighter ones indicate alterations on allele B (alleles defined arbitrarily). a) Segment-based profile: all segments are displayed as having the same size. b) Sized profile: Alteration are shown on a genome-scale using the dominant copy-number state for each minor cytoband. Grey areas indicate areas where profiling was not possible due to previous filtering steps.

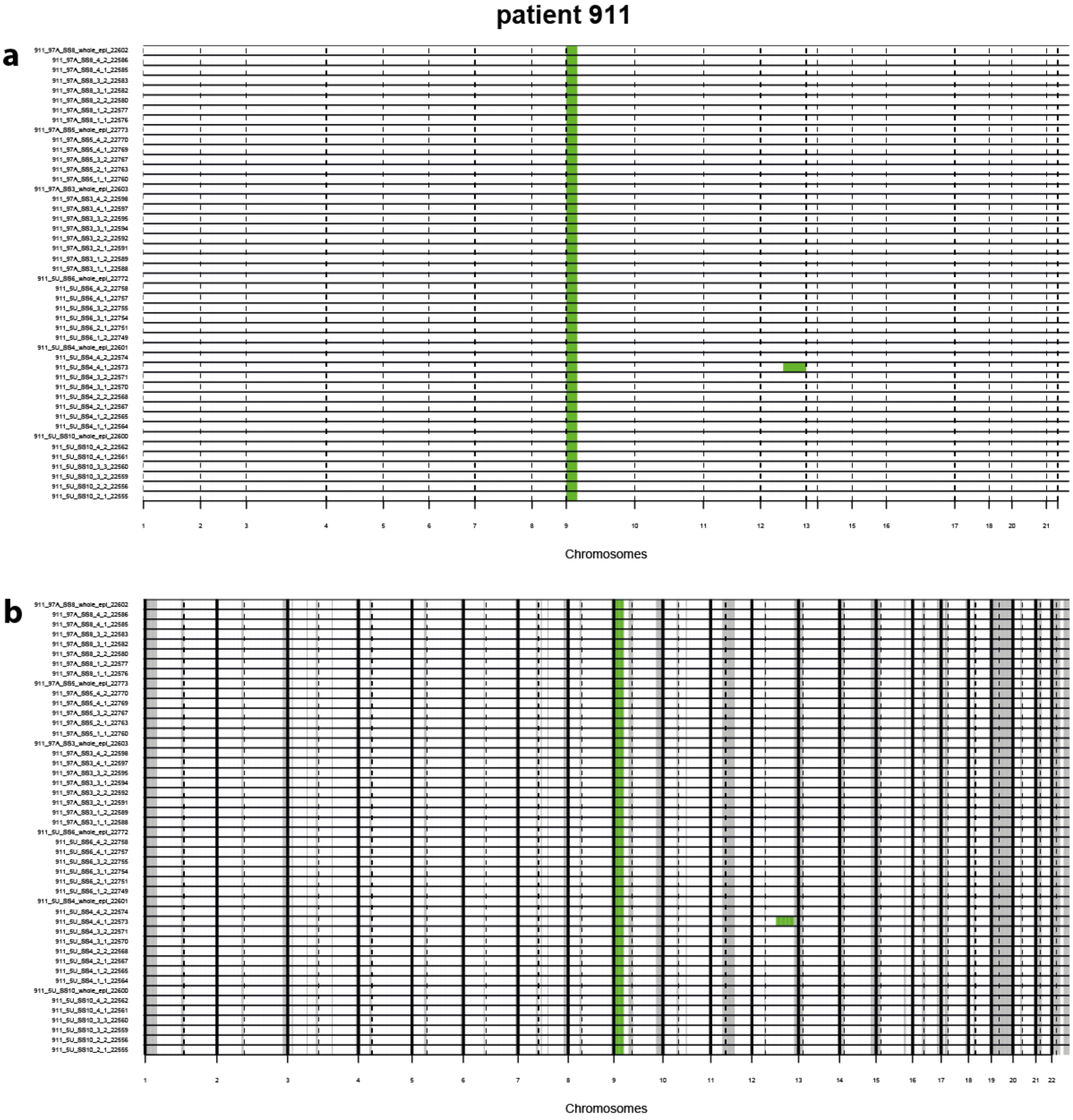

Supplementary Figure 79: Final phased profile (patient 911-NP). Blue segments indicate a loss, red ones a gain, green ones a copy-neutral loss of heterozygosity, with white ones showing no alteration. Darker colours indicate alterations on allele A, lighter ones indicate alterations on allele B (alleles defined arbitrarily). a) Segment-based profile: all segments are displayed as having the same size. b) Sized profile: Alteration are shown on a genome-scale using the dominant copy-number state for each minor cytoband. Grey areas indicate areas where profiling was not possible due to previous filtering steps.

Breakpoint definition

Genomes were defined as having undergone genome doubling if the ploidy reported by ASCAT was strictly greater than 3, diploid otherwise. Breakpoints were defined as the association between a chromosomal location, an allele as defined by prior phasing, and either a gain or a loss compared to the preceding segment. At the start of the chromosome where there was no previous segment, we substituted the inferred median copy number of the sample. Divergent breakpoints were defined as those that were observed in one sample but not the other. When calculating percentages, only "informative" breakpoints were considered, i.e. those demarcating a copy number alteration observed in at least one sample in the patient.

Maximum parsimony phylogenetic analyses

For each patient p with Ns samples, the list of all Np breakpoints observed at least once was used to create a *breakpoint matrix* of size Ns x Np. The presence and absence of each breakpoint in each sample was coded by '1' if a given sample presented a given breakpoint, '0' otherwise. This was done separately for whole genome breakpoints and fragile site breakpoints. Phylogenetic trees were produced using maximum parsimony and Fitch optimization via the *phangorn* R package[4], which was also used to calculate the Robinson-Foulds distances[5]. Evolutionary distances were defined as the sum of the branch lengths separating crypts and/or biopsies on the maximum parsimony *phangorn* phylogeny. Evolutionary diversity was defined as the mean evolutionary distance, for example among all biopsy-biopsy comparisons within a patient.

In order to determine whether the two data sets provided compatible phylogenetic information, we pooled together the Npwg whole genome breakpoints and the Npfs fragile site breakpoints. Breakpoints totaling Npwg were then taken at random to build a first tree, while the Npfs remaining were used to build a second tree; the topological-only Robinson-Foulds distance was then calculated between them. The process was repeated 250 times to create an expected distance distribution which was then compared to the observed distance between the two real trees, giving a one-tailed p-value.

Principal component analysis-based color schemes

The Ns x Np sample-specific breakpoint matrices were used to provide each sample with a color based on breakpoint presence/absence. A principal component analysis was performed on each matrix to extract the first three principal components, on which singular value decomposition (SVD) was performed for each sample. The principal components were normalized so that all three distributions ranged from 0 to 200 across samples. Each sample was therefore assigned 3 values between 0 and 200, which served as a basis for color on a 256-bit RGB scheme. The three principal components (in order) corresponded to red, green and blue. Values were limited to 200 to avoid obtaining colors too close to white (255 in all three component colors).

Bayesian phylogenetics: methodological development

Bayesian phylogenetic analyses were performed using a modified version of the BEAST 1.8 software[6] adapted to the usage of phased integer SCA state data. We implemented a novel substitution model that parametrizes the acquisition of SCAs based on three rates (gain, loss, and LOH), modifies the likelihood calculation by incorporating a branch connecting the most recent common ancestor of the

sample to the last common ancestor with an unaltered genome (LUCA), and incorporates an acquisition bias correction adapted from the one by Lewis[7]. Some of these modifications are built upon re-implementations of strategies developed by Kostadinov et al[8].

Each variable fragment constitutes an evolutionary character in our model and its state indicates the number of copies of the original two alleles present in the sample. This model assumes that each fragment evolves as an independent and identically distributed (i.i.d.) random variable.

SCA mutation matrix

We designed and implemented a continuous-time non-reversible Markov chain to model the SCA mutation process. Its rate matrix (**Supplementary Fig. 80**) is parameterized by three rates, each modeling one evolutionary event: gain, loss and conversion. *Gain* corresponds to the acquisition of an additional copy of one of the original alleles, *loss* to the loss of a pre-existing copy of one of the original alleles, and *conversion* to the substitution of one of the original alleles for the other. Rates in the diagonal are set so that the sum of transition rates leaving a given state and the rate of keeping the same state equals 0 for all states. Conceptually, our model has an infinite number of states; however, we implemented a version limited to 6 copies per locus (fragment), since we consider that our SCA calling pipeline cannot accurately call states with a larger number of copies. The non-reversibility of this process complicates its usage with clock models, since it cannot be normalized so that the total flux of states equals 1. In order to circumvent this issue, the matrix can be normalized by the total SCA rate per fragment and allele (i.e., the sum of the three parameters of the matrix). This is only necessary when using more than one clock in the tree (i.e., random local clock).

This model was implemented in BEAST as a new substitution model. Since it only has two free parameters, we estimated two rates relative to the third. We relied on the general data type of BEAST to input our SCA states, recoding the 28 biallelic states of our rate matrix as different alphanumerical characters. We did not obtain a simple closed-form of the transition probability matrix and therefore carried out the matrix exponentiation using the numerical approach already implemented in BEAST.

	0/0	0/1	1/0	1/1	0/2	1/2	2/0	2/1	2/2	0/3	1/3	2/3	3/0	3/1	3/2	3/3	0/4	1/4	2/4	4/0	4/1	4/2	0/5	1/5	5/0	5/1	0/6	6/0
**0/0**	0	0	0	0	0	0	0	0	0	0	0	0	0	0	0	0	0	0	0	0	0	0	0	0	0	0	0	0
**0/1**	d	$-(d+g)$	0	0	g	0	0	0	0	0	0	0	0	0	0	0	0	0	0	0	0	0	0	0	0	0	0	0
**1/0**	d	0	$-(d+g)$	0	0	0	g	0	0	0	0	0	0	0	0	0	0	0	0	0	0	0	0	0	0	0	0	0
**1/1**	0	d	d	$-2(d+g+c)$	c	g	c	g	0	0	0	0	0	0	0	0	0	0	0	0	0	0	0	0	0	0	0	0
**0/2**	0	$2d$	0	0	$-2(d+g)$	0	0	0	0	$2g$	0	0	0	0	0	0	0	0	0	0	0	0	0	0	0	0	0	0
**1/2**	0	0	0	$2d$	d	$-(3(d+g)+2c)$	0	$2\times\frac{1}{2}\times c$	g	c	$2g$	0	0	0	0	0	0	0	0	0	0	0	0	0	0	0	0	0
**2/0**	0	0	$2d$	0	0	0	$-2(d+g)$	0	0	0	0	0	$2g$	0	0	0	0	0	0	0	0	0	0	0	0	0	0	0
**2/1**	0	0	0	$2d$	0	$2\times\frac{1}{2}\times c$	d	$-(3(d+g)+2c)$	g	0	0	0	c	$2g$	0	0	0	0	0	0	0	0	0	0	0	0	0	0
**2/2**	0	0	0	0	0	$2d$	0	$2d$	$-4(d+g+\frac{2}{3}c)$	0	$2\times\frac{2}{3}\times c$	$2g$	0	$2\times\frac{2}{3}\times c$	$2g$	0	0	0	0	0	0	0	0	0	0	0	0	0
**0/3**	0	0	0	0	$3d$	0	0	0	0	$-3(d+g)$	0	0	0	0	0	0	$3g$	0	0	0	0	0	0	0	0	0	0	0
**1/3**	0	0	0	0	0	$3d$	0	0	$3\times\frac{1}{3}\times c$	d	$-2(2(d+g)+c)$	g	0	0	0	0	c	$3g$	0	0	0	0	0	0	0	0	0	0
**2/3**	0	0	0	0	0	0	0	0	$3d$	0	$2d$	$-(5(d+g)+3c)$	0	0	$3\times\frac{2}{4}\times c$	$2g$	0	$2\times\frac{3}{4}\times c$	$3g$	0	0	0	0	0	0	0	0	0
**3/0**	0	0	0	0	0	0	$3d$	0	0	0	0	0	$-3(d+g)$	0	0	0	0	0	0	$3g$	0	0	0	0	0	0	0	0
**3/1**	0	0	0	0	0	0	0	$3d$	$3\times\frac{1}{3}\times c$	0	0	0	d	$-2(2(d+g)+c)$	g	0	0	0	0	c	$3g$	0	0	0	0	0	0	0
**3/2**	0	0	0	0	0	0	0	0	$3d$	0	0	$3\times\frac{2}{4}\times c$	0	$2d$	$-(5(d+g)+3c)$	$2g$	0	0	0	0	$2\times\frac{3}{4}\times c$	$3g$	0	0	0	0	0	0
**3/3**	0	0	0	0	0	0	0	0	0	0	0	$3d$	0	0	$3d$	$-6(d+g+\frac{3}{5}c)$	0	0	$3\times\frac{3}{5}\times c$	0	0	$3\times\frac{3}{5}\times c$	0	0	0	0	0	0
**0/4**	0	0	0	0	0	0	0	0	0	$4d$	0	0	0	0	0	0	$-4(d+g)$	0	0	0	0	0	$4g$	0	0	0	0	0
**1/4**	0	0	0	0	0	0	0	0	0	0	$4d$	$4\times\frac{1}{4}\times c$	0	0	0	0	d	$-(5(d+g)+2c)$	g	0	0	0	c	$4g$	0	0	0	0
**2/4**	0	0	0	0	0	0	0	0	0	0	0	$4d$	0	0	0	$4\times\frac{2}{5}\times c$	0	$2d$	$-(6d+\frac{16}{5}c)$	0	0	0	0	$2\times\frac{4}{5}\times c$	0	0	0	0
**4/0**	0	0	0	0	0	0	0	0	0	0	0	0	$4d$	0	0	0	0	0	0	$-4(d+g)$	0	0	0	0	$4g$	0	0	0
**4/1**	0	0	0	0	0	0	0	0	0	0	0	0	0	$4d$	$4\times\frac{1}{4}\times c$	0	0	0	0	d	$-(5(d+g)+2c)$	g	0	0	c	$4g$	0	0
**4/2**	0	0	0	0	0	0	0	0	0	0	0	0	0	0	$4d$	$4\times\frac{2}{5}\times c$	0	0	0	0	$2d$	$-(6d+\frac{16}{5}c)$	0	0	0	$2\times\frac{4}{5}\times c$	0	0
**0/5**	0	0	0	0	0	0	0	0	0	0	0	0	0	0	0	0	$5d$	0	0	0	0	0	$-5(d+g)$	0	0	0	$5g$	0
**1/5**	0	0	0	0	0	0	0	0	0	0	0	0	0	0	0	0	0	$5d$	$5\times\frac{1}{5}\times c$	0	0	0	d	$-2(3d+c)$	0	0	c	0
**5/0**	0	0	0	0	0	0	0	0	0	0	0	0	0	0	0	0	0	0	0	$5d$	0	0	0	0	$-5(d+g)$	0	0	$5g$
**5/1**	0	0	0	0	0	0	0	0	0	0	0	0	0	0	0	0	0	0	0	0	$5d$	$5\times\frac{1}{5}\times c$	0	0	d	$-2(3d+c)$	0	c
**0/6**	0	0	0	0	0	0	0	0	0	0	0	0	0	0	0	0	0	0	0	0	0	0	$6d$	0	0	0	$-6d$	0
**6/0**	0	0	0	0	0	0	0	0	0	0	0	0	0	0	0	0	0	0	0	0	0	0	0	0	$6d$	0	0	$-6d$

g= gain rate, d= deletion rate, c= conversion rate

Supplementary Figure 80: SCA rate matrix implemented in BEAST. *g*=gain rate, *d*=loss rate, *c*=conversion rate.

Last common ancestor with unaltered genomic state

We implemented a last common ancestor with unaltered genomic state (LUCA) in both tree model and likelihood calculation, thus assuming that all samples shared a common ancestor with an unaltered genome. This allowed us to estimate the time in which the BE lesion started its development and also helped to make better use of the non-reversible SCA rate matrix. LUCA is implemented as an extra degree-1 node that becomes the root of the tree, which is connected to the most recent common ancestor (MRCA) by a new branch. Therefore, the BEAST implementation of the likelihood calculation had to be modified to carry out the Felsenstein pruning algorithm[9] in a degree-1 node. Moreover, the partial likelihood at the root is multiplied by the partial likelihood of the assumed root state (i.e., partial likelihood of one for the wild type state and zero for the rest).

Acquisition bias correction

The evolutionary characters used in our analysis were obtained by joint segmentation of the SNP array data (i.e., filtered logRs and BAFs), and therefore, by definition, all observed characters varied. Moreover, the number of invariant fragments is not measurable or easy to estimate, as it would correspond to the invariable proportion of the total potential fragments the BE tissue could eventually generate in a given patient. Nevertheless, considering only variable fragments constitutes a sampling bias, which we predict would strongly affect the estimation of absolute SCA mutation rates. This bias is well known in other kinds of data, such as restriction sites[10], single nucleotide polymorphisms[11] and morphological characters[7]. In order to mitigate this bias, we implemented the conditional likelihood correction[7] modified for the SCA characters of our model (see [12] for an equivalent implementation for DNA characters) repurposing BEAST code by Alekseyenko et al.[13]. Reconstituted approaches could not be used in our data due to the difficulty of estimating the number of invariant fragments.

Genome doubling

Our phylogenetic method does not model genome doubling (GD). However, polyploidy is common in cancer cells, and is present in our data. We predicted that the presence of genome doubling would systematically bias our evolutionary parameter estimates, such as SCA mutation rate, tree topology and branch lengths. We developed two alternative data pre-processing strategies intended to alleviate this model misspecification, and carried out a simulation study (Supplementary Note 1) in order to choose the best, which we used in our analyses. The chosen strategy consisted of re-coding the SCA states, making them relative to the estimated integer baseline. Thus, states were divided by the estimated baseline state. If the division remainder was not zero, the resulting state was rounded up or down at random for each position, in order to minimize the mutation rate estimation bias. The integer baseline was calculated as the rounded division of the mean copy number state per allele.

Bayesian phylogenetic analysis

In order to estimate SCA mutation rates we developed a new phylogenetic method (PISCA) implemented as a BEAST 1.8[6] plugin (available at https://github.com/adamallo/PISCA). PISCA analyzes sequences of phased integer SCA states using a new model with three main components: (1) a SCA substitution model, (2) an extra branch connecting the most recent common ancestor of

the sample to the last common ancestor with an unaltered genome (LUCA), and (3) acquisition bias correction.

For the 6 patients who had sufficient mutations to perform the analysis, we analyzed single-crypt and whole-biopsy SCA data separately to obtain estimates of the phylogenetic tree, SCA mutation rate (sum of gain, loss and conversion rate), LUCA age and effective population size. We then compared the estimates obtained at the crypt and whole-biopsy level. We used both a strict molecular clock model, which assumes that all branches of the phylogeny have the same (constant) SCA mutation rate, and a random local clock model, which allows for different branches of the phylogeny to have different SCA mutation rates[14]. This allowed us to estimate changes in SCA mutation rate in specific BE cell lineages during progression.

The two phased allele-specific copy number matrices per individual (one for each allele) were translated into SCA states, applying our pre-processing strategy intended to mitigate the GD bias. Fragments called as wild-type in all samples from a patient were discarded in order to appropriately use the acquisition bias correction. These molecular data, together with the patient date of birth, sequence dates (tip dates) and prior distributions (**Supplementary Tables 1-2**) were used to generate the BEAST input files. We performed two analyses, one using a strict molecular clock, and one using the random local clock model[14]. Patients 256-NP and 911-NP were not analyzed, since the number of variable sites for at least one of their data-sets was too low (3 and 1, respectively).

**Parameter**	**Prior distribution**
Tree	Coalescent model
Effective population size	1/x
SCA gain acquisition rate	1/x
Relative SCA loss rate	Exponential(1)
Relative SCA conversion rate	Exponential(1)
LUCA age (years from the present)	Uniform[lbe-fbe,lbe-dob]

Supplementary Table 1: List of Bayesian phylogenetic priors for the strict molecular clock analysis. *lbe*: epoch time of the last biopsy of the given patient, *fbe*: epoch time of the first biopsy of the given patient, *dob*: date of birth of the given patient.

Parameter	Prior distribution
Tree	Coalescent model
Effective population size	1/x
Mean clock rate	1/x
Number of rate changes	Poisson(0.693)
Relative rate multipliers	Gamma(rate=0.5,scale=2)
Relative SCA loss rate	Exponential(1)
Relative SCA conversion rate	Exponential(1)
LUCA age (years from the present)	Uniform[lbe-fbe,lbe-dob]

Supplementary Table 2: List of Bayesian phylogenetic priors for the random local clock model. *lbe*: epoch time of the last biopsy of the given patient, *fbe*: epoch time of the first biopsy of the given patient, *dob*: date of birth of the given patient.

Each data set was analyzed using three independent 250M-long MCMC chains. Parameters and trees were sampled each 25K generations. Intra- and inter-chain convergence for all logged parameters and trees were assessed using Tracer (Rambaut A et al. 2014. Available from http://beast.bio.ed.ac.uk/Tracer) and RWTY[15]. One extra chain sampling only from the priors was run per dataset in order to check that the results were not driven by the priors. Parameter and tree estimation were carried out on the combination of the 3 chains after discarding the first 10% of the samples as burnin (i.e., 27000 posterior samples). Three datasets required the usage of the slower Metropolis-coupled alternative with three chains with default heating parameters in order to mix properly and achieve convergence. We performed a simulation study (see the G*enome Doubling* section of the supplementary methods) using both algorithms to ensure that their results were comparable. With the strict molecular clock model, effective sample sizes (ESSs) were high for all parameters and samples, getting close to the maximum 9000 in most datasets and parameters, with a global minimum per replicate of 241 (data not shown). The random clock model analysis showed the same performance except for some rate-change parameters for the biggest crypt-based analyses. Parameter point estimates were obtained using the mean of the posterior sample, while maximum credibility trees with median heights were used as tree point estimates. The estimated population size parameter (*PNe*) is a composite parameter *PNe=Ne*tau*, where *Ne* is the effective population size and *tau* the generation time length. In order to obtain final *Ne* estimates we assumed 2 different *tau* values, 50 and 7 days. The probability of two posterior samples being equal was calculated as their overlap using the R package *birdring*[16].

SUPPLEMENTARY NOTE 1: SIMULATION STUDY

Introduction

The current implementation of PISCA does not model genome doubling (GD). However, polyploidy is common in cancer cells, and it is present in our data. We predict that the presence of genome doubling will systematically bias our evolutionary parameter estimates, like SCA acquisition rate, tree topology, and branch lengths. In order to characterize this systematic bias, we carried out a simulation study with different scenarios. Moreover, this allowed us to benchmark the two data pre-processing strategies we developed to alleviate this expected bias and to ensure that results obtained with the MCMC and MC3 BEAST algorithms were comparable.

GD-like genotypes can arise due to both real GD events or due to baseline estimation errors in the CNV calling procedure. In order to assess the relative performance of the different strategies tackling these two scenarios we developed a simulation strategy for the two of them and added a negative control with no doublings. We also predicted that acquisition rate strongly affects the accuracy of the different strategies and included different rates in our analyses accordingly.

Methods

Data pre-processing strategies

We developed two data pre-processing strategies intended to reduce the systematic bias induced by GD events. The inclusion of a third strategy in the form of negative control left us with the following three strategies:

> -*none*: CNV states are directly translated from the simulated number of allele copies (i.e., neglecting GD). For clarification, we refer to the two original copies (one per chromosome) as A and B alleles, independently of their identity. Thus, the state 2:1 would correspond to a gain of the copy A (2 copies) and the original copy number state of the allele B (1 copy).
>
> -*max2*: CNV states are restricted to deviate only in one unit from the estimated baseline for each of the alleles (i.e., we only assign either gain, baseline or loss for each of the alleles). Thus, we are considering a 9-state matrix, namely: 1:1, 2:1, 0:1, 1:2, 1:0, 2:2, 2:0, 2:2, 0:0 (A state : B state).
>
> -*baseline*: CNV states are calculated relative to the estimated integer baseline. Thus, simulated states are divided by the estimated baseline state. If the division remainder is not zero, the resulting state is randomly rounded. We added this strategy to avoid biasing the acquisition rate estimation. We estimated the integer baseline as the rounded division of the mean copy number state per allele.

SCA simulator

We developed and implemented in Python an in-house simulation software that simulates copy number states evolving down a user-specified tree according to our SCA substitution model. It also incorporates two genome doubling models, one parameterized by a constant GD rate and another by an exponentially growing GD rate. The implementation of the second is based on waiting-time scaling; a well-known strategy used to simulate exponential population growth in coalescent simulators. We included this strategy to reflect that GD is more common late in progression. This simulator also implements an option to duplicate a given number of leaves chosen at random, feature intended to simulate GD-like genotypes originated by baseline estimation errors. Finally, this

piece of software also considers the previously discussed pre-processing strategies and can directly generate BEAST input files. Importantly, it depends on *DendroPy 4.1.0* [17]

Simulation pipeline

We simulated the trees using CoalEvol7.3.5 [18] under the coalescent model with an exponentially expanding population and dated tips. We assayed two tree sizes (11 and 56 leaves) corresponding to the median number of taxa for the two groups respectively (progressors and nonprogressors) taking into consideration only the single-crypt samples. The other tree-simulation parameters were fixed and kept between biologically reasonable values (**Supplementary Table 3**). We generated ten replicates (trees) per condition.

**Parameter**	**Value**
Time between time points	5 years
Generation time	1 day
Haploid effective population size in the tips	90000 individuals
Effective population size exponential growth rate	1.563e-3

Supplementary Table 3: Comprehensive list of fixed tree-simulation parameters. The exponential growth rate was parameterized assuming a LUCA age of 20 years before the present. Note that we parameterized the effective population size in haploid fashion since we assume that cancer cells do not undergo recombination, and therefore must be modeled as haploid individuals.

These trees were used as input for our SCA simulator in order to generate genetic data under 54 different simulation scenarios; result of the combination of two tree sizes, three acquisition rates, three pre-processing strategies and three doubling models (**Supplementary Table 4**). Some CNV-simulation parameters were fixed and kept between biologically reasonable values (**Supplementary Table 5**).

**Parameter**	**Values**
Tree size (number of tips)	Nonprogressor (11), progressor (54)
Acquisition rate	0.0003, 0.003, 0.03 SCAs/year/site/allele
GD model	No GD, simulated GD, leaf doubling
Pre-processing strategy	None, max2, baseline

Supplementary Table 4. Comprehensive list of variable parameters and values across simulation scenarios. All 54 combinations were assayed.

Parameter	Value
Patient age	60 years
Number of loci	100 loci
Gain rate (relative to acquisition rate)	1
Loss rate (relative to acquisition rate)	1
Conversion rate (relative to acquisition rate)	1
GD rate *	0.001 GD per year
GD rate growth rate *	0.31
Number of tips to duplicate	2 tips

Supplementary Table 5. Comprehensive list of fixed SCA-simulation parameters. Parameters indicated with * are only applicable in certain simulation scenarios. The exponential growth rate of the GD rate has been parameterized to get to a final value of 0.5 at the tips.

Estimation

We performed the phylogenetic estimation on each dataset using three independent runs of 20M MCMC generations in PISCA. We sampled the posterior distribution every 2000 generations and used the priors shown in **Supplementary Table 6**. We checked for proper mixing and convergence using Tracer [19] and RWTY [15] in a random replicate of each combination. We discarded a 10% of the posterior samples in each estimation replicate as burnin, and combined the rest to perform the data analysis.

Parameter	Prior distribution
Tree	Coalescent model
Effective population size	1/x
SCA gain rate	LogNormal(-4,2.5)
Relative loss rate	Exponential(1)
Relative conversion rate	Exponential(1)
LUCA age (years from the present)	Uniform[5,60]

Supplementary Table 6. List of priors used for the phylogenetic estimation in BEAST.

We calculated point estimates of both tree topology and acquisition rate in order to compare them with the true values, thus calculating the accuracy of the different methods under various conditions. We used the maximum clade credibility tree and the mean of the posterior acquisition rates as point estimates of tree topology and acquisition rate respectively. We measured the tree error using the normalized topological RF distance (calculated using the R package *phangorn* (Schliep, 2011)) and the acquisition rate accuracy in terms of relative error. We replicated the study using the MC3 algorithm only for one estimation run per sample in order to save computational resources.

Statistical analyses

We compared the distribution topological and acquisition rate errors for the three pre-processing strategies in all the assayed combinations of acquisition rate, genome doubling, and tree size. We assigned groups based on Wilcoxon signed-rank tests (alpha=0.05) corrected for multiple testing (for each parameter combination) using the Bonferroni correction. We also compared the distribution of errors of the scenarios without genome doubling and post-processing against post-processed duplicated scenarios using the Wilcoxon rank-sum test. Finally, we calculated the overlap between the posterior distribution of the SCA rates estimated using the MCMC and the MC3 algorithms in order to assess their comparability.

Results and discussion

This simulation study was designed to understand the systematic bias introduced by genome doubling in PISCA and to select the best pre-processing strategy to handle it. In order to do so, we compared the distribution of tree topology and acquisition rate estimation errors for three post-processing strategies (*none*, *max2* and *baseline*), across different combinations of acquisition rate (0.0003, 0.003 and 0.03 chromosomal alterations per allele copy, per locus, per year), genome doubling model (genome doubling and leaf genome doubling) and tree sizes (11 and 54 leaves).

The analysis without post-processing strategies (i.e., *none*) allowed us to characterize the bias introduced by genome doubling. Without GD we observed a clear positive correlation between acquisition rate and estimation accuracy for the two simulated tree sizes (nonprogressors-like, **Supplementary Figure 81A,D**; progressors-like, **Supplementary Figure 82A,D**). With the highest assayed acquisition rate the mean accuracies were very high attending to the two parameters (Nonprogressors: RF=0.089 ± 0.022, acquisition rate relative error=0.13 ± 0.023; Progressors: RF=0.17 ± 0.018, acquisition rate relative error= 0.066 ± 0.010). However, GD reduced the accuracy of both errors for the two assayed tree sizes. The highest effect was shown in the tree accuracy for the smaller tree size, in which the doubling-induced error completely neutralized the increase in accuracy with the acquisition rate (**Supplementary Figure 81B-C**). These results confirm the need of a data pre-processing procedure in order to reduce these estimation biases.

Both data pre-processing strategies significantly reduced both errors in most conditions in the presence of genome doubling for 11-leaf (**Supplementary Figure 81B,C,E,F**) and 54-leaf trees (**Supplementary Figure 82B,C,E,F**). However, the ***max2*** strategy significantly **increased** the two **errors** in conditions with 54-leaf trees without genome doubling (**Supplementary Figure 82A,D**) and the acquisition rate in conditions with doubled leaves (**Supplementary Figure 82F**). The alternative strategy –*baseline*– was less aggressive and did not significantly increase the estimation error in any of the assayed conditions, being still as good as *max2* in most, except for one condition (with

genome doubling, the highest mutation rate for the 11-leaf tree, only in the estimation of the acquisition rate) (**Supplementary Figure 81E**). Moreover, *baseline* did effectively get rid of the error induced by leaf genome doubling in all scenarios and by genome doubling in most, being able to make the differences non-significant between the scenario without doubling and those with post-processing. The exceptions for the 54-leaf trees were the acquisition rate estimation with 0.003 rate (p=0.0089, 0.18 ± 0.037 vs 0.35 ± 0.044) and the tree topology estimation with 0.03 rate (p=0.0027, 0.17 ± 0.018 vs. 0.30 ± 0.030). For the 11-leaf trees, the exception was the acquisition rate with genome doubling and 0.03 rate (p=0.035, 0.13 ± 0.023 vs 0.26 ± 0.046).

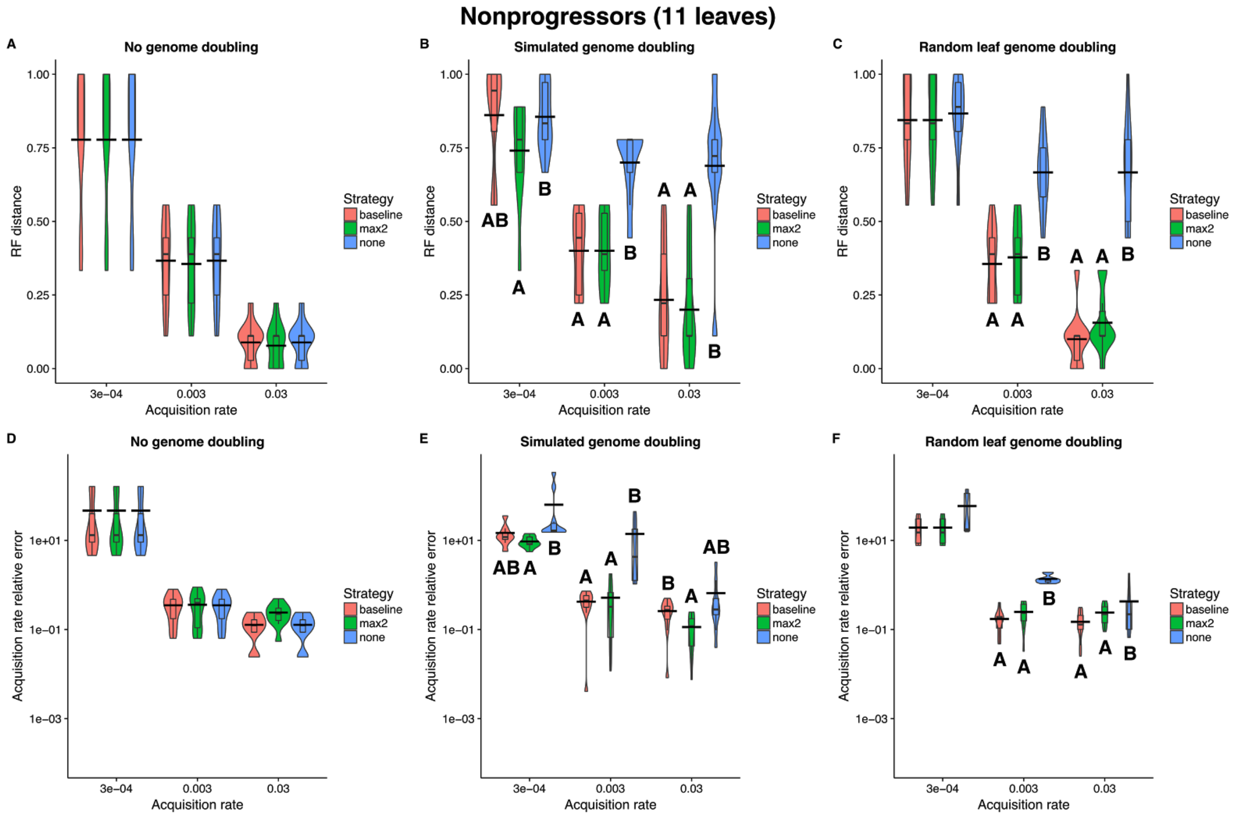

Supplementary Figure 81. Reconstruction error for 11-leaf simulations. Each violin plot corresponds to the distribution of errors of the ten replicates assayed for the indicated simulation condition. The first row (panels A-C) corresponds to tree topology estimation and the second to acquisition rate estimation errors. Different columns represent different GD models: the 1st column (A, D) corresponds to scenarios with no GD, the 2sd (B, E) to simulated GD and the 3rd (C, F) to 2 random tips artificially duplicated *a-posteriori*. In each plot, the y-axis indicates the error measure (RF distance for trees, relative error for acquisition rates) and the x-axis indicates different acquisition rates (in log-scale). Importantly, the **y-axis is given in log-scale for acquisition rate errors only**. Each strategy constitutes a data series identified with a different color (red=baseline, green=max2, blue=none). Capital letters close to the violins indicate groups of significantly different population mean errors for each combination of acquisition rate and genome doubling strategy (Wilcoxon signed-rank tests, alpha=0.05, Bonferroni corrected for three comparisons). Conditions without letters do not show any significant difference (i.e., all distributions pertain to the same group).

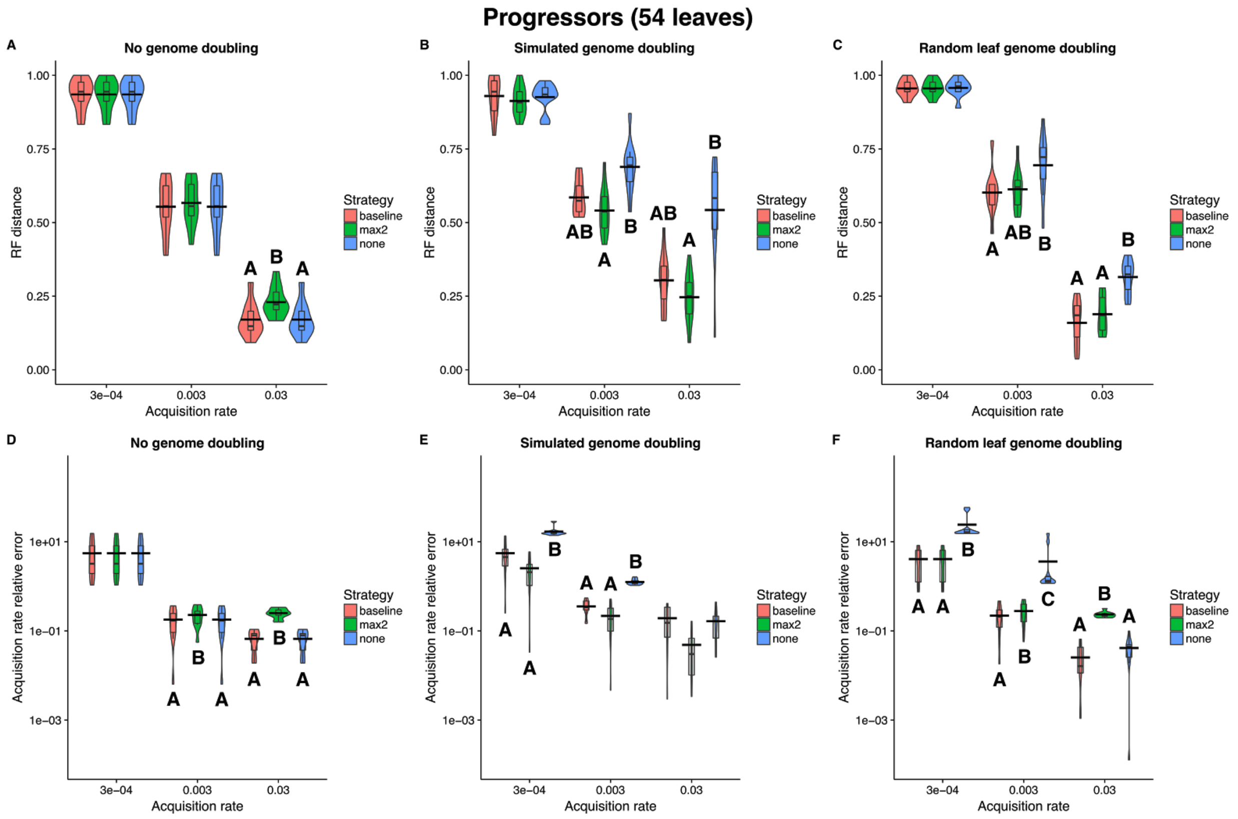

Supplementary Figure 82. Reconstruction error for 54-leaf simulations. Each violin plot corresponds to the distribution of errors of the ten replicates assayed for the indicated simulation condition. The first row (panels A-C) corresponds to tree topology estimation and the second to acquisition rate estimation errors. Different columns represent different GD models: the 1st column (A, D) corresponds to scenarios with no GD, the 2sd (B, E) to simulated GD and the 3rd (C, F) to 2 random tips artificially duplicated *a-posteriori*. In each plot, the y-axis indicates the error measure (RF distance for trees, relative error for acquisition rates) and the x-axis indicates different acquisition rates (in log-scale). Importantly, the **y-axis is given in log-scale for acquisition rate errors only**. Each strategy constitutes a data series identified with a different color (red=baseline, green=max2, blue=none). Capital letters close to the violins indicate groups of significantly different population mean errors for each combination of acquisition rate and genome doubling strategy (Wilcoxon signed-rank tests, alpha=0.05, Bonferroni corrected for three comparisons). Conditions without letters do not show any significant difference (i.e., all distributions pertain to the same group).

The results of the MCMC and MC3 algorithms were highly comparable, with a 93% of the simulation conditions showing a posterior probability of being the same >0.8 (**Supplementary Figure 83**).

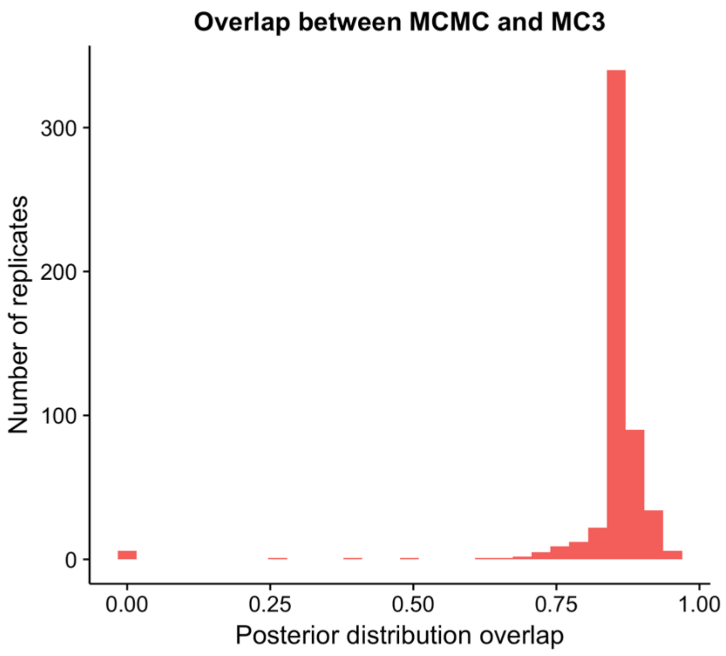

Supplementary Figure 83. Histogram of the overlap between posterior distributions obtained with the MCMC and MC3 algorithms for all simulation conditions. The posterior distribution corresponds to the SCA rate parameter.

Conclusions

This simulation study allowed us to ascertain that we needed a data pre-processing step in order to analyze genome-doubled data in PISCA and to select the most appropriate one. We chose the *baseline* strategy since it makes the error distribution of genome-doubled conditions indistinguishable from their non-doubled counterparts in most conditions and it does not significantly increase the estimation error in any of them. Moreover, the MCMC and MC3 algorithms generated very similar posterior distributions in a vast majority of conditions, and therefore we conclude that they are comparable.

REFERENCES

1. Wang, K. *et al.* PennCNV: an integrated hidden Markov model designed for high-resolution copy number variation detection in whole-genome SNP genotyping data. *Genome Res.* **17,** 1665–74 (2007).
2. Nilsen, G. *et al.* Copynumber: Efficient algorithms for single- and multi-track copy number segmentation. *BMC Genomics* **13,** 591 (2012).
3. Van Loo, P. *et al.* Allele-specific copy number analysis of tumors. *Proc. Natl. Acad. Sci. U. S. A.* **107,** 16910–5 (2010).
4. Schliep, K. P. phangorn: phylogenetic analysis in R. *Bioinformatics* **27,** 592–3 (2011).
5. Robinson, D. F. & Foulds, L. R. Comparison of phylogenetic trees. *Math. Biosci.* **53,** 131–147 (1981).
6. Drummond, A. J., Suchard, M. A., Xie, D. & Rambaut, A. Bayesian Phylogenetics with BEAUti and the BEAST 1.7. *Mol. Biol. Evol.* **29,** 1969–1973 (2012).
7. Lewis, P. O. A likelihood approach to estimating phylogeny from discrete morphological character data. *Syst. Biol.* **50,** 913–25
8. Kostadinov, R. L. *et al.* NSAIDs Modulate Clonal Evolution in Barrett's Esophagus. *PLoS Genet.* **9,** e1003553 (2013).
9. Felsenstein, J. Evolutionary trees from DNA sequences: a maximum likelihood approach. *J. Mol. Evol.* **17,** 368–76 (1981).
10. Felsenstein, J. PHYLOGENIES FROM RESTRICTION SITES: A MAXIMUM-LIKELIHOOD APPROACH. *Evolution (N. Y).* **46,** 159–173 (1992).
11. Kuhner, M. K., Beerli, P., Yamato, J. & Felsenstein, J. Usefulness of single nucleotide polymorphism data for estimating population parameters. *Genetics* **156,** 439–47 (2000).
12. Leaché, A. D., Banbury, B. L., Felsenstein, J., de Oca, A. N.-M. & Stamatakis, A. Short Tree, Long Tree, Right Tree, Wrong Tree: New Acquisition Bias Corrections for Inferring SNP Phylogenies. *Syst. Biol.* **64,** 1032–47 (2015).
13. Alekseyenko, A. V, Lee, C. J. & Suchard, M. A. Wagner and Dollo: a stochastic duet by composing two parsimonious solos. *Syst. Biol.* **57,** 772–84 (2008).
14. Curtius, K. *et al.* A Molecular Clock Infers Heterogeneous Tissue Age Among Patients with Barrett's Esophagus. *PLOS Comput. Biol.* **12,** e1004919 (2016).
15. Warren, D. L., Geneva, A. J. & Lanfear, R. RWTY (R We There Yet): An R Package for Examining Convergence of Bayesian Phylogenetic Analyses. *Mol. Biol. Evol.* **34,** 1016–1020 (2017).
16. Korner-Nievergelt, F. & Robinson, R. A. Introducing the R-package 'birdring'. *Ringing Migr.* **29,** 51–61 (2014).
17. Sukumaran, J. & Holder, M. T. DendroPy: a Python library for phylogenetic computing. *Bioinformatics* **26,** 1569–71 (2010).
18. Arenas, M. & Posada, D. Simulation of Genome-Wide Evolution under Heterogeneous Substitution Models and Complex Multispecies Coalescent Histories. *Mol. Biol. Evol.* **31,** 1295–1301 (2014).
19. Rambaut, A., Suchard, M., Xie, D. & Drummond, A. Tracer v1.6, Available from http://beast.bio.ed.ac.uk/Tracer. (2014).
