# Supplementary material for: Evolution of Barrett’s esophagus through space and time at single-crypt and whole-biopsy levels
Source: Nat Commun. 2018 Feb 23;9:794. doi: 10.1038/s41467-017-02621-x (PMC5824808; doi:10.1038/s41467-017-02621-x)
Supplement: Supplementary file 3 — Description of Additional Supplementary Files [file 41467_2017_2621_MOESM3_ESM.pdf]

Description of Supplementary Files

File Name: Supplementary Data 1
Description: **Segments removed after manual inspection.** Chr: chromosome; nLogR: number of logR-informative probes; nBAF: number of BAF-informative probes.
